# The complex set of internal repeats in SpTransformer protein sequences result in multiple but limited alternative alignments

**DOI:** 10.3389/fimmu.2022.1000177

**Published:** 2022-10-18

**Authors:** Megan A. Barela Hudgell, L. Courtney Smith

**Affiliations:** Department of Biological Sciences, George Washington University, Washington, DC, United States

**Keywords:** immune genes, *Strongylocentrotus purpuratus*, bioinformatics, multiple sequence alignment, sea urchin

## Abstract

The *SpTransformer* (*SpTrf*) gene family encodes a set of proteins that function in the sea urchin immune system. The gene sequences have a series of internal repeats in a mosaic pattern that is characteristic of this family. This mosaic pattern necessitates the insertion of large gaps, which has made alignments of the deduced protein sequences computationally difficult such that only manual alignments have been reported previously. Because manual alignments are time consuming for evaluating newly available SpTrf sequences, computational approaches were evaluated for the sequences reported previously. Furthermore, because two different manual alignments of the SpTrf sequences are feasible because of the multiple internal repeats, it is not known whether additional alternative alignments can be identified using different approaches. The bioinformatic program, PRANK, was used because it was designed to align sequences with large gaps and indels. The results from PRANK show that the alignments of the internal repeats are similar to those done manually, suggesting multiple feasible alignments for some regions. GUIDANCE based analysis of the alignments identified regions that were excellent and other regions that failed to align. This suggests that computational approaches have limits for aligning the SpTrf sequences that include multiple repeats and that require inserted gaps. Furthermore, it is unlikely that alternative alignments for the full-length SpTrf sequences will be identified.

## Introduction

### The *SpTrf* gene family

Pathogens exert pressure on organisms that often lead to the generation and maintenance of diverse immune gene families that are beneficial for resistance to infection (1). Consequently, large expanded immune gene families are common because of the evolutionary host-pathogen arms race that takes place over a range of evolutionary time scales and leads to the selection of duplicated and altered immune genes (2–5). Examples of expanded immune gene families can be found throughout the tree of life, from the human KIR gene families (6) to the resistance or *R* genes in higher plants [reviewed in (4)], in addition to many others (7–10). The purple sea urchin, *Strongylocentrotus purpuratus*, has a number of expanded gene families that are predicted to encode proteins with immune function (3, 11–15) such as the *Toll-Like-Receptor* (*TLR*) genes (16), the *NOD* and *NALP* genes (17, 18), *cysteine rich scavenger receptor* genes (17, 19, 20), *IL-17* genes (15), and the *SpTransformer* (*SpTrf*) genes (2, 21, 22). The *SpTrf* genes are upregulated upon immune challenge (11–14) and isolated native SpTrf proteins opsonize and augment phagocytosis for some species of bacteria (23). A recombinant SpTrf protein, rSpTrf-E1, has binding affinity for subsets of bacteria and several different types of PAMPs (24, 25). To date, 17 of these genes have been identified in the sequenced genome of *S. purpuratus* (2), however gene copy number is likely to vary among individuals based on the *SpTrf* genes amplified from the genomes of three sea urchins that show 120 different sequences (22).

The structure of the *SpTrf* gene family in the sequenced sea urchin genome shows tight clustering, sequence similarity, segmental duplications of the DNA that include entire genes, repeats in the second exon, and short tandem repeats in the flanking regions (2, 22, 26, 27). The genes in this family are short (< 2 kb) and are composed of two exons and a short intron. The exons encode a short leader and a highly variable mature protein (12, 13, 22). The variability in the second exon is derived from blocks of sequences, called elements (abbreviated Er to indicate elements in the repeat-based alignment), that form sequence mosaics called element patterns and are a unique aspect of the second exon that are not a product of alternative splicing (13, 22, 28). The element mosaicism is further complicated by the presence of six types of repeats (R) (Types 1 – 6), which are found in the coding sequence of the second exon (11, 13, 29). Two categories of genes have been identified and termed the long genes and the short genes (21, 27). These categories are based on the length of these genes as determined by the number of elements that they contain. The long genes have the maximum number of elements (25 of 27) and are the *A* and *G* element pattern *SpTrf* genes, whereas the short genes have less than 19 elements and are composed of all the other element patterns. In general, these two categories of genes therefore encode long (L) and short (S) proteins. These characteristics lead to interesting questions regarding the evolution of this gene family given that the appearance of these highly variable mosaic genes have been proposed to require extensive insertions, deletions, and perhaps partial gene conversions in the coding regions (26, 27). Previous work by Buckley et al. (29) have hypothesized an evolutionary history for Type 1 repeats that began with a single, last common ancestral sequence that subsequently underwent duplication, recombination, and deletion events to generate up to four, tandem Type 1 repeats of slightly different sequences in the extant long genes. Likewise, the repeats in the 3′ end of the second exon are composed of a complex, interspersed pattern of repeat Types 2 – 6 that have more conserved sequences than the Type 1 repeats, but show increased variation based on the interspersed pattern of these repeats. For example, the deduced L proteins contain a repeat pattern of (6-2-4-2-3-5)_2_-4, while the deduced S proteins have repeat variations such as (6-2-3-4-3-2-3-5-4), (6-2-3-5-4), and (4-2-3-5-4), among others (30). Furthermore, the encoded proteins have a number of simple short repeats in the glycine rich region at the N terminal end and in the histidine rich region at the C terminal end. All of these sequence characteristics, which make the SpTrf proteins an interesting family, also make them difficult to align and evaluate because the repeats enable different potential alignments (12, 13, 30).

The first alignment of the deduced proteins from the *SpTrf* cDNAs was done manually and reported as the ‘cDNA-based alignment’ that described the mosaic pattern of elements (13). However, this alignment had a large gap of 414 nucleotides in the second exon for the short genes and did not take into account the internal repeats (12). A second manual alignment explored a possible alternative and correlated the edges of the elements with the edges of as many of the internal repeats as possible, which resulted in the ‘repeat-based alignment’ that was deemed equally optimal to the cDNA-based alignment (22). To date, all SpTrf analyses have been done by employing one or the other of these alignments because the multiple sequence alignment (MSA) algorithms available at the time, PAUP (31) or ClustalV (32), did not optimize the alignments of the repeats or the elements. However, the problem of continuing to employ manual alignments is that they are both time consuming to construct and may inadvertently incorporate human error resulting in sub-optimally aligned regions.

In general, alignments of sequenes with multiple and duplicated internal repeats are not typically investigated and reported. Consequently, it is noteworthy that two different manual alignments for the SpTrf protein sequences are feasible leading to the question of i) whether manual alignments are biased in any way, ii) whether there are errors introduced from manual alignments, and iii) whether there are other possible alignments that may be identified computationally. New alignment tools may have the potential to produce different alignments of the SpTrf sequences that would avoid biases and errors that might be present in the manual alignments. This effort has the additional goal of identifying an alignment tool that is capable of streamlining the alignment of these genes for further downstream studies including evolutionary analysis and identification of potential genomic events that led to the generation of the unusual structure of the *SpTrf* genes that are composed of elements (2, 22, 27). Here we explore the ability of the MSA tool, phylogenetically-aware algorithm (PRANK) [http://wasabiapp.org/software/prank/ (33)], to align the protein sequences deduced from the *SpTrf* genes that have been identified in the sea urchin genome (27, 34). Although we used the DNA sequences as the input dataset, PRANK translates the sequences into deduced amino acid sequences, or aligns the DNA sequences based on codons, and reports the alignments as amino acid sequences. Hence, we report the results as alignments of the deduced proteins. PRANK was used both with and without a provided guide tree, and the alignments were compared to the manual repeat-based alignment and to alignments from the more widely used ClustalW alignment tool (35). We further used GUIDANCE to score each individual column (each position in the alignment) from PRANK and compared the scores to the GUIDANCE scores obtained by ClustalW alignments with the aim of identifying regions of low alignment confidence. We show that employing PRANK as a strictly computational alignment approach results in alignments that show regions of excellent alignment of matching elements but also poorly aligned or mismatched regions. Furthermore, the use of these computational approaches does not identify any additional optimal alignments for full-length SpTrf sequences but does suggest multiple possible alignments for specific repeats in regions of the sequences.

## Materials and methods

### SpTrf sequence data

*SpTrf* gene sequences (n = 134) from the genomes of four sea urchins were compiled from the *SpTrf* genes identified in the sea urchin BAC library (animal G (34, 36)), and from three animals (animals 2, 4, 10) as reported previously (22). This dataset was composed of 53 genes from animal 2, 27 genes from animal 4, 37 genes from animal 10, and 17 genes from animal G. A subset of gene sequences (n = 49) from each sea urchin spanning each *SpTrf* element pattern was selected from the 134 genes and used to generate representative sequence alignments (15 sequences from animal 2, 10 sequences from animal 4, 15 sequences from animal 10, and 9 sequences from animal G). The first exon and the intron were removed to focus the analysis on the second exon that encodes the mature protein (**Figure S1**).

### Repeat-based alignment

The *SpTrf* gene sequences were translated to the deduced protein sequences in BioEdit [ver 7.2.5 (37)] and aligned manually using the approach reported previously (22, 27).

### Phylogenetic trees

MEGAX [http://www.megasoftware.net/ (38)] was used to generate phylogenetic trees from the repeat-based alignments of the second exon with the maximum likelihood method under pre-set parameters and bootstrap iterations set to 500. This tree was provided as a guide tree to PRANK for alignment analysis. Neighbor joining trees were produced by webPRANK (https://www.ebi.ac.uk/goldman-srv/webPRANK/) as a by-product of the PRANK alignment process. Additional neighbor joining trees were produced from the PRANK alignments in GUIDANCE2 (http://guidance.tau.ac.il) using MEGAX.

### PRANK alignments

PRANK [http://wasabiapp.org/software/prank/ (33)] alignments were generated using webPRANK with and without a guide tree (**Figures S2**; **S3**). The guide tree was a maximum likelihood tree based on the repeat-based alignment (see above) in MEGAX. Standard parameters were used in webPRANK (gap rate, 0.05; gap length, 5; K, 2.0) and the sequences were set to align by translated codons and to trust insertions (+F). Substitution scoring was set to relaxed and, when a guide tree was not provided, the guide tree generation was done using ClustalW2 (39) to produce a neighbor joining tree. DNA alignment anchoring allowed the use of CHAOS anchors (40). An additional set of PRANK alignments were evaluated using GUIDANCE2 [http://guidance.tau.ac.il/ver2/ (41–43)], which employed standard PRANK parameters as described above and was set to trust insertions (+F). Bootstrap guide-trees of 100 iterations were generated, which resulted in 400 alternative alignments before the GUIDANCE2 score was calculated.

### ClustalW multiple sequence alignment

ClustalW (35) in BioEdit (37) was used to generate an alignment for comparison to the repeat-based and PRANK alignments. The deduced protein sequences were aligned using standard parameters with full multiple alignment and the bootstrap iterations set to 1000 for a neighbor joining guide tree. An additional ClustalW alignment was done through GUIDANCE2 using 100 bootstrap iterations (maximum), which resulted in 400 alternative alignments to calculate the GUIDANCE scores.

## Results

### Different phylogenetic guide trees employed in PRANK result in a variety of alignments

**Figure 1:**
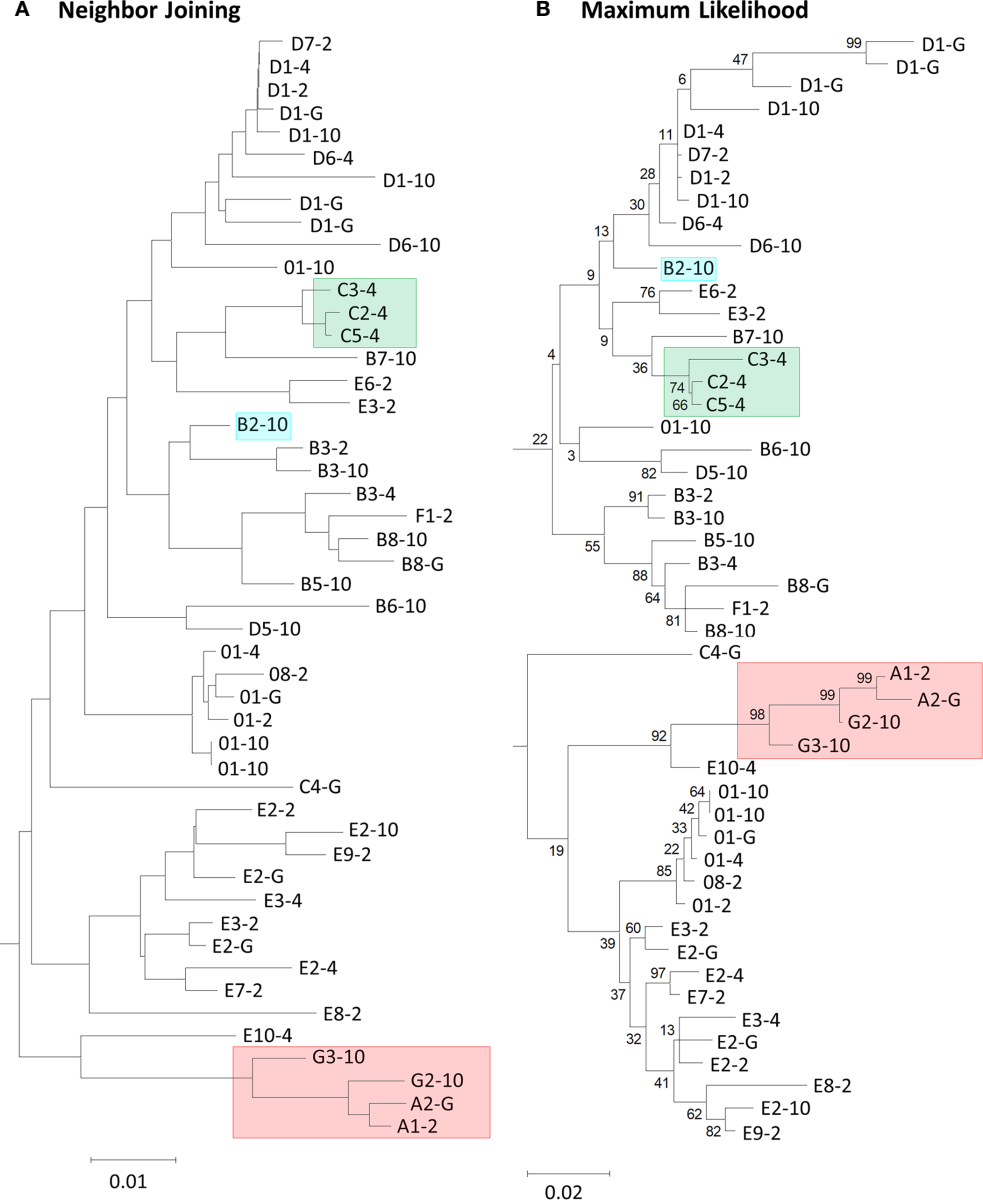
Neighbor joining and maximum likelihood phylogenetic trees show variation in the placement of deduced SpTrf protein sequences and in the branch lengths. **(A)** The neighbor joining tree is a by-product of, and is used for a PRANK alignment in webPRANK. **(B)** The maximum likelihood tree was generated from the manual repeat-based alignment using BioEdit (37) in MEGAX with the deduced amino acid sequences of 49 SpTrf proteins. Highlighted boxes in the trees indicate proteins of interest (see text). Bootstrap values generated from 500 iterations are indicated for each tree. Scale bars at the bottom of the trees indicate branch lengths based on percent nucleotide substitutions per site.

PRANK is a sequence alignment tool that allows the placement of large insertions and deletions (indels) into an MSA rather than only point mutations (33). PRANK is reported to be ideal for short gene sequences from closely related species because it requires the construction of evolutionary homology, or phylogenetic tree, in its alignment program. Employing PRANK was thought to be ideal for aligning the *SpTrf* gene sequences with extensive indels because they are short, closely related members of a gene family, and have been sequenced from four *S. purpuratus* animals. The second exon of the *SpTrf* genes is composed of a mosaic of repeats and elements that require large gap insertions in alignments and have only been reported as manual alignments of the deduced protein sequences (11–13, 22). Preliminary analyses of all 134 sequences resulted in non-informative phylogenetic trees with short branch lengths and low bootstrap scores (data not shown). Consequently, 49 representative sequences were chosen based on unique sequence structure from among the 134 sequences so that each unique sequence was only represented once per individual. Two PRANK alignments were constructed from the translated codons of 49 *SpTrf* gene sequences. One alignment employed a neighbor joining guide tree (NJGT) produced in webPRANK (NJGT-PRANK) (**Figure 1A**). However, PRANK alignments are known to be particularly sensitive to errors in trees, based on comments by the PRANK designers (http://wasabiapp.org/software/prank/prank_differences/), and are due to low confidence in branch placement and short branch lengths. Errors in tree structure may lead to errors in the translated codon alignments. Therefore, a maximum likelihood tree, which is considered a more robust method for phylogeny construction of highly variable sequences (44), was produced from the manual repeat-based alignment of the deduced protein sequences and provided as a guide tree (MLGT) for webPRANK (MLGT-PRANK) (**Figure 1B**). These PRANK alignments were subsequently compared to the repeat-based alignment as a means to judge reliability of the PRANK alignments. However, because the repeat-based alignment was done manually and did not include multiple iterations to identify an optimal alignment, the accuracy cannot be determined computationally for comparison to the computer based alignment algorithms such as PRANK to determine which alignment may appear optimal. Consequently, it cannot be determined which of the alignments is truly optimal for this gene family.

#### The Type 1 repeats result in variable alignments

**Figure 2:**
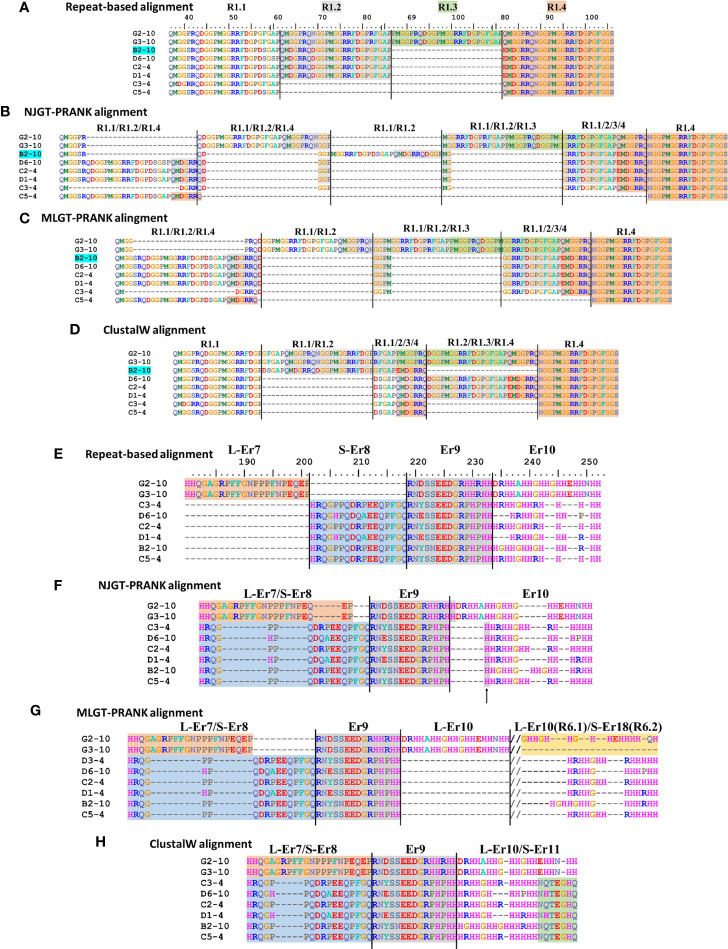
Four different alignment methods show variations in the final MSAs. SpTrf protein sequence alignments are shown based on a selected regions from the full-length alignments in **Figures S1-S4**. **(A–D)** Various alignments of Type 1 repeats are the outcome of the various approaches that are used. **(E–H)** Er7 – Er10 are located in about the middle of the proteins and show variation in the alignment based on the approach that is used. Alignments are **(A, E)** a repeat-based manual alignment done in BioEdit (ver. 7.2.5), **(B, F)** an alignment generated in webPRANK using a neighbor joining guide tree (NJGT-PRANK), **(C, G)** an alignment generated in webPRANK using a maximum likelihood guide tree (MLGT-PRANK), and **(D, H)** an alignment done in ClustalW in BioEdit (ver. 7.2.5) without manual correction. The color highlights of regions in **(A)** indicate elements Er3 to Er5 as identified in the repeat-based alignment and are applied to the alignments in **(B–D)**. Er11 is immediately to the right of the alignments in **(E, F)** and is not shown. The deduced protein names are located to the left and reflect the element patterns of the short (S) proteins (B to D element patterns), the long (L) proteins (G element pattern), and the sea urchin (4 and 10) from which the sequences were obtained. The ruler above the repeat-based alignment indicates the amino acid location. The dashes (–) indicate the insertion of artificial gaps in all alignments where the sequences do not match. The//in **(G)** indicates sequence in the alignment that is not shown. Type 6 repeats are included for L-Er10 and S-Er18 in **(G)**. Element boarders are indicated by black vertical lines. The alignments in **(E–H)** are edited relative to the alignments in **Figures S2-S4** to remove irrelevant gaps for the reduced number of sequences in this illustration. These edits do not change the alignment.

**Figure 3:**
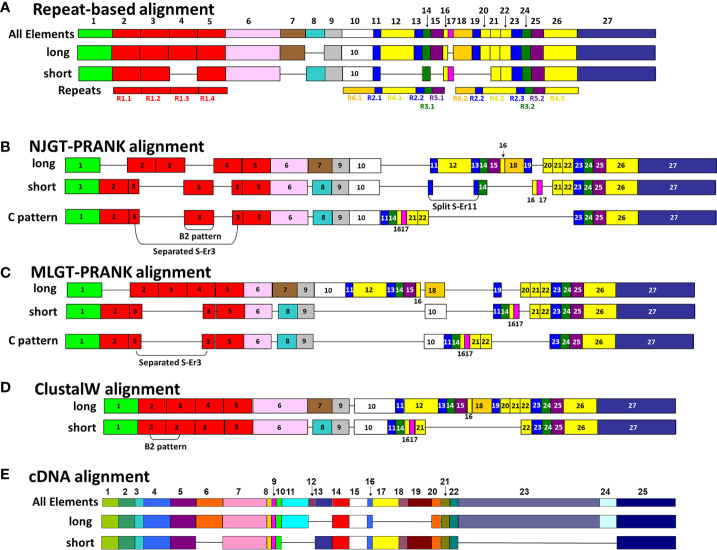
A representative map of sequence alignments based on multiple methods show the variations in final MSAs. **(A)** A graphical representation of the manual SpTrf repeat-based alignment is shown, as done in BioEdit according to Buckley and Smith (22). All elements (Er) encoded in the second exon are numbered at the top of **(A, E)**. The Er numbering in **(A)** applies to alignments in **(B**–**D)**. For each sequence alignment, representative element patterns for L proteins and S proteins are shown. The elements (colored boxes) and gaps (horizontal lines) are indicated for each representative sequence. Below the alignment in **(A)**, the repeats are numbered and indicated by rectangles of identical coloring as the elements in which they are located. **(B)** A graphical representation is shown of the NJGT-PRANK alignment for representative L and S protein sequences. **(C)** A graphical representation is shown of the MLGT-PRANK alignment for the representative L and S protein sequences. **(D)** A graphical representation of the ClustalW alignment as done in BioEdit is shown for the representative L and S protein sequences. **(E)** A graphical representation of the manual cDNA alignment for the representative L and S protein sequences is shown for comparison purposes.

The imperfect, tandem Type 1 repeats positioned at the N terminal end of the mature SpTrf proteins (29) are designated as R1.1 to R1.4 and make up elements Er2 to Er5 in the repeat-based alignment (**Figures 2A**, **3A**). The Type 1 repeat region includes five simple repeats of glycines that are the basis for the glycine-rich region of the proteins (29). These sequence characteristics make the Type 1 repeats particularly difficult to align including establishing the edges of the repeats. In the repeat-based alignment, the Type 1 repeats are generally 25 aa long with different deduced proteins having between 0 to 4 complete repeats [**Figures 2A**, **S1** (22, 27)]. The Type 1 repeats were aligned differently in NJGT-PRANK, compared to the repeat-based alignment, and were expanded into six elements of variable length rather than four repeats of equal length (**Figures 2B**, **S2**). The edges of the Type 1 repeats were also different from the manual repeat-based alignment and resulted in additional gaps and repeats that varied in length from 25 to 37 aa. One repeat was only present in one sequence, B2-10, which consisted of a combination of R1.1 and R1.2. The difference in this alignment may have been an outcome of the neighbor joining phylogenetic guide tree (**Figure 1A**, aqua box), which placed the B2-10 sequence on a different branch compared to its position in the maximum likelihood tree that resulted from the repeat-based alignment (**Figure 1B**, aqua box). The low bootstrap numbers in the region of the maximum likelihood tree that included B2-10 were the likely basis for the alignment difference (**Figure 1B**). To determine whether conflicting positions of B2-10 within the phylogenetic trees was a source of the differences in the alignment of the Type 1 repeats between the NJGT-PRANK alignment and the repeat-based alignment, a maximum likelihood phylogenetic tree was produced from the repeat-based alignment (**Figure 1B**). In the maximum likelihood phylogenetic tree, B2-10 was basal to a D1 clade (**Figure 1B**, blue box), whereas in the neighbor joining guide tree generated by webPRANK for the NJGT-PRANK alignment, B2-10 clustered with two B3 genes and was positioned within a B clade (**Figure 1A**, aqua box). The maximum likelihood phylogenetic tree generated from the repeat-based alignment was then used as a guide tree in PRANK that resulted in a new MLGT-PRANK alignment. Results showed that R1.1 and R1.2 in B2-10 aligned well with R1.1 and R1.2 in the other SpTrf protein sequences (**Figures 2C**; **S3**). However, in the MLGT-PRANK alignment, the edges of the Type 1 repeats as defined by the repeat-based alignment, were differently aligned resulting in five repeats of variable length rather than four repeats of 25 aa (**Figures 2A–C**). This was due to L-R1.1 (in the A and G element patterns of the L proteins) that did not align with the corresponding S-R1.1 in the S proteins that make up all the other element patterns in the MLGT-PRANK alignment (**Figures 2C**; **S3**). Similarly, L-R1.2 aligned separately from S-R1.2. Rather, the C terminal end of S-R1.2 aligned with the C terminal end of L-R1.3, and the N terminal end of S-R1.2 aligned with L-R1.1 and L-R1.2 (compare **Figure 2A** with **2B, C**; **Figures S2**, **S3**). This resulted in short aligned regions of two to four aa, a result that was also observed in the alignment from NJGT-PRANK. The control alignment was carried out in ClustalW because the PRANK algorithm used a guide tree generated in ClustalW2 to produce alignments (**Figure S4**). In the ClustalW output, the alignment of the Type 1 repeats was most similar to the repeat-based alignment and the sequences had fewer gaps than in the alignments from NJGT-PRANK and MLGT-PRANK (**Figure 2D**). Both ClustalW and the MLGT-PRANK alignment resulted in five Type 1 repeats but the edges and sizes of each repeat was different. The C terminal end of S-R1.1 aligned with the C terminal end of L-R1.2, and some of the S-R1.2 aligned with L-R1.3 (**Figure 2D**). For the case of the proteins with C3 and C5 element patterns that were missing S-R1.2, the N terminal end of S-R1.4 aligned with the N terminal end of S-R1.3 (**Figure 2D**). Interestingly, B2-10 was the only sequence with three Type 1 repeats where S-R1.2 aligned optimally with L-R1.2 in the ClustalW alignment. The other sequences with three Type 1 repeats all aligned S-R1.2 with L-R1.3 (**Figure S4**), which was different from the manual repeat-based alignment for the Type 1 repeats (29). Although these results suggested that the Type 1 repeats have alternative alignments, all outcomes resulted in longer alignments with more gaps compared to the repeat-based alignment, which was particularly evident for PRANK that employed the structures of the guide trees to inform the alignments.

#### L-Er7 and S-Er8 align together consistently

Elements Er6 through Er10 as defined by the repeat-based alignment are positioned in the histidine rich region of the deduced proteins (**Figures 2E**, **3A**). They do not contain internal short repeats and were likely to align well by computational approaches. Er6 and Er9 in the repeat-based alignment were positioned similarly in the alignments from NJGT-PRANK, MLGT-PRANK, and ClustalW (**Figures 2E–H**, **S1-4**). The only difference was a single histidine in the NJGT-PRANK alignment that was shifted from Er9 into Er10 (**Figure 2F**, arrow). Alternatively, L-Er7 and S-Er8, which are not shared between the L and S proteins (22), were not distinguished as separate elements in any of the alignment approaches even though their sequences were dissimilar (**Figures 2E–H**). L-Er7 and S-Er8 were combined with many gaps, islands of two to four aa, or an overlap of non-matching sequences in the three computational alignment approaches (**Figures 2F–H**). Overall, these results indicated that the alignments of Er6 to Er10 were different for all of the computational approaches and included regions of both matched and completely mismatched sequences.

#### Er10 alignment by computational approaches

Er10 is a key element in most sequences and has been employed advantageously for naming each of the genes (*A* to *G* element patterns) because it has a highly variable number of histidines among the genes, and specific sequence variants of Er10 are associated with specific mosaics of elements (**Figure 2E**) (13). Although Er10 is highly variable, it aligned between Er9 and Er11 with distinct borders for all proteins in NJGT-PRANK (**Figures 2F**; **S2**) although it was longer with more gaps compared to the manual repeat-based alignment (**Figure 2E**). The alignment of the Er9 to Er11 region in MLGT-PRANK, specifically for the L proteins, was distinct because rather than aligning L-Er10 with S-Er10, a large gap of 86 aa was inserted into the S proteins to align S-Er10 with L-Er18 (**Figure S3**). The gap may have been inserted because both L-Er10 and S-Er18 encode Type 6 repeats (R6.1 and R6.2 respectively) and have very similar sequences (**Figure 2G**). This variation may be considered an alternative alignment to the alignment of this region in the repeat-based alignment. The ClustalW alignment for the Er9 to Er11 region, resulted in an alternative alignment that was short and without large gaps (**Figure 2H**). L-Er7 aligned with S-Er8, and the C terminal end of L-Er10 aligned with S-Er11 even though the sequences did not match. Overall, the computational alignments for Er10 produced by PRANK based approaches led to fewer regions of dissimilar sequence compared to ClustalW, in addition to generating a possible alternative alignment for Type 6 repeats in both S-Er10 and L-Er18.

### Interspersed repeats at the C terminal end of the SpTrf proteins align well using PRANK

Most of the elements in the C terminal region of the SpTrf proteins that span Er10 through Er27 (excluding Er16 and Er17 that do not have repeats) are a series of Type 2 to Type 6 repeats that make up a duplicated, interspersed element pattern of 6-2-4-2-3-5 (**Figure 3A**) (29). This region of the L proteins is difficult to align computationally because of the multiple copies of these repeats. Although the S proteins have fewer repeats, the repeats among these proteins show more sequence variability compared to the L proteins (29). To enable alignments for this region of the SpTrf sequences, large gaps have been inserted in different locations of the S proteins to align matching elements and repeats with the L proteins, which has resulted in the two different manual alignments (22). However, whether there are additional optimal alignments for this region based on strictly computational analyses, rather than manual alignments has not been addressed. When alignments were carried out using NJGT-PRANK or MLGT-PRANK approaches, the edges of the repeats were maintained with those of the elements (**Figures 3B, C**; **S2**, **S3**). However, some of the duplicated repeats in the L proteins were not always aligned with the corresponding repeats in the S proteins relative to the repeat-based alignment. In the NJGT-PRANK alignment, S-Er11 was split and aligned with both L-Er11 and L-Er13, which introduced a large gap at the location of L-Er12 (**Figures 3B**; **S2**). This was because both Er11 and Er13 include highly similar Type 2 repeats (R2.1 and R2.2). Furthermore, while both L-Er14 and S-Er14 were aligned, S-Er16 did not align with the corresponding L-Er16 element, but instead was positioned with a gap in the C terminal end of L-Er19 (**Figures 3B**; **S2**). Interestingly, the region of S-Er11 to S-Er22 specifically in the C element pattern proteins did not align with the corresponding elements in either the L proteins or the other S proteins, but instead were aligned with a large gap inserted in all of the other proteins. This outcome was likely due to the structure of the NJGT where the three C element pattern proteins clustered together with proteins of the B7, E6, and E3 element patterns (**Figure 1A**, green box). This association was surprising because the B7, E6, and E3 proteins only have S-Er11, S-Er14, and S-Er25 to S-Er27, whereas the C proteins have the additional elements S-Er16, S-Er17, and S-Er21 to S-Er24 (excluding the C5 pattern protein which does not include S-Er23 to S-Er25) (**Figure S1**). This difference in the numbers of elements between the C proteins and the B7, E6, and E3 proteins may explain why these elements in the C proteins aligned separately from the other S proteins. However, unlike the B2 pattern protein, when a guide tree was provided to the PRANK method (rather than webPRANK generating the guide tree) it did not change the alignment of the C element pattern proteins (**Figure 3B**), with a large gap inserted into the other proteins as in the NJGT-PRANK alignment, although the gap insertion was in a different location (**Figures 3C**, **S3**). This was likely due to the position of the C pattern proteins in the MLGT that was similar to their position in NJGT (**Figure 1**, green boxes).

Because S-Er10 was aligned with L-Er18 in the MLGT-PRANK alignment, the region including L-Er11 to L-Er16 located to the N terminal side of L-Er18 were aligned with a large gap in the S proteins (**Figures 3C**; **S3**). This also shifted the alignment of the elements in the S proteins such that S-Er11 aligned with L-Er19 (they contain R2.1 and R2.3, respectively), resulting in a large gap in the L proteins that corresponded with S-Er14, S-Er16, and S-Er17 (**Figure 3C**). The region of Er21 to Er27 in both S and L proteins did not show mismatched sequences in both types of PRANK approaches (**Figures 3B, C**). Although there were small differences in alignments from the PRANK methods, it was noteworthy that repeats of similar sequence were aligned or that a gap was inserted to prevent an alignment of non-similar repeats. This type of gap insertion was not observed in the ClustalW alignment in which regions of similar repeats were not always aligned and a single large gap was inserted in the S proteins to compensate for the length of the L proteins (**Figure 3D**). This meant not only that S-Er11 was aligned with the end of L-Er10, but that S-Er14, S-Er16, S-Er17, and S-Er20 were aligned with L-Er12 even though this was a mismatch with no sequence similarity (**Figure 3D**). However, the C terminal region of both S and L proteins including Er22 to Er27 were aligned with matching sequences. In general, these results indicated that there were other possible alignments of these proteins, and that the PRANK and repeat based alignments had a reduced number of dissimilar amino acids that were aligned together compared to the ClustalW alignment.

### GUIDANCE evaluation of PRANK alignments indicates robustness for SpTrf sequences

**Figure 4:**
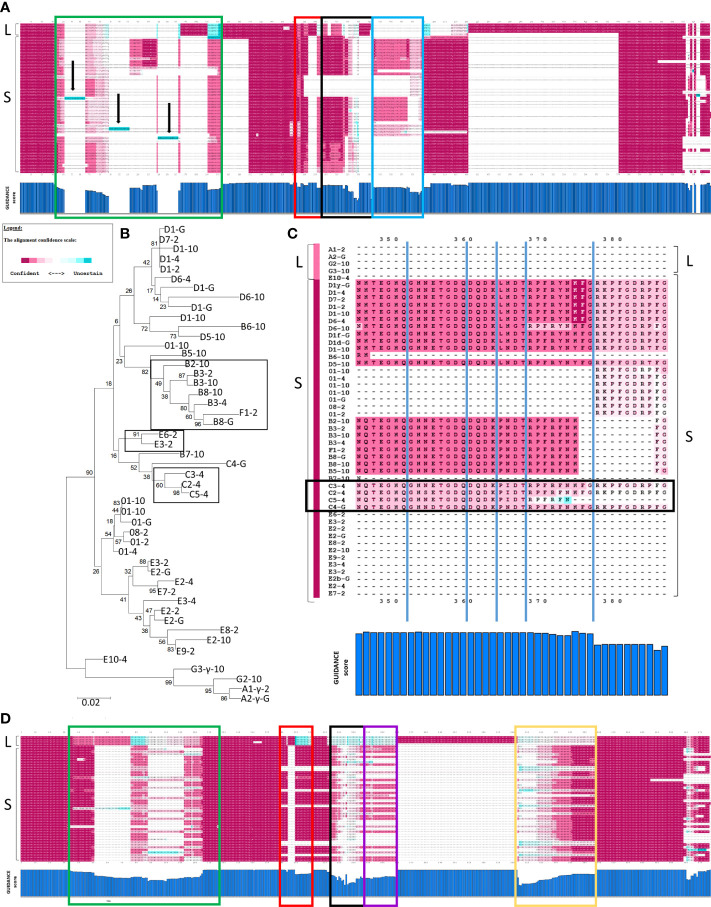
GUIDANCE scores indicate that PRANK generates a more robust alignment than ClustalW. **(A)** A full-length MSA is shown with GUIDANCE confidence scores calculated in GUIDANCE2-PRANK of representative SpTrf protein sequences. The top three sequences are the L proteins, and the rest are S proteins. The confidence score at each amino acid position is colored to correlate with confident (magenta) to moderate (white) to uncertain (aqua) (see legend). The X axis of the bar graph below the MSA illustrates the GUIDANCE scores for each column (aa position) in the MSA and the Y axis indicates a score from 0 (no bar) to 1 (full bar). Arrows indicate sequences (aqua highlights) in which there are gaps in all but one sequence resulting in a GUIDANCE score of 0. Boxes in green, red, black and blue indicate different regions of low (< 0.93) GUIDANCE scores. **(B)** A neighbor joining tree is shown as done in MEGAX and is based on the PRANK alignment in **(A)**. The boxes indicate clades of proteins that are described in the main text. Bootstrap values were generated from 500 iterations and are indicated for each node. The scale bar at the bottom of the tree indicates the branch length that is based on substitutions per site. **(C)** The alignment of elements Er11 to Er22 are expanded from the alignment in **(A)**. The confidence scores and colors for each aa position are indicated in the legend. The GUIDANCE scores are shown at the bottom of the alignment as a bar graph as in **(A)**. The names of the protein sequences are shown to the left and the color bar to the left of the name represents the GUIDANCE score for each sequence (see legend). Dashes (–) indicate gaps inserted into the alignment where the sequences do not match. The region in the black box indicates the C element pattern proteins. **(D)** A MSA is shown for a ClustalW alignment of representative SpTrf protein sequences. The GUIDANCE confidence scores for each amino acid position as calculated in GUIDANCE2 are indicated in colors (see legend). Colored boxes (green, red, black, purple, and yellow) indicate regions of low GUIDANCE scores. The GUIDANCE scores for each column or position in the alignment is shown in the bar graph below as in **(A)**.

GUIDANCE (http://guidance.tau.ac.il/ver2) is used to evaluate the robustness of the PRANK alignments based on the reliability of columns (positions in an alignment), sequences, and individual amino acids for each sequence in the alignment. GUIDANCE scores are defined as the confidence level for specific positions in an MSA. The GUIDANCE2 server evaluates MSAs as a tertiary PRANK alignment that uses GUIDANCE2 (GUIDANCE2-PRANK) to report and visualize the GUIDANCE scores as bar graphs for each column. It was noted that the GUIDANCE2-PRANK alignment was different from the alignment generated by NJGT-PRANK. This was not unexpected given that PRANK has been documented as resulting in slightly different alignments from different runs of the same set of sequences (http://wasabiapp.org/software/prank/#Methods). Furthermore, while the GUIDANCE2 server only provided the GUIDANCE scores for the base or standard MSA, defined as the first alignment generated upon which additional phylogenetic trees and alignments were generated to calculate the GUIDANCE scores, additional MSAs were available as a SuperMSA file that contained alternative alignments generated by the program as a concatenation of the top 20 alternative PRANK aligned MSAs (data not shown). The base alignment from GUIDANCE2-PRANK showed the same type of alignment variations as those from the NJGT-PRANK alignment, which were also evident with most of the SuperMSA alignments to various degrees. For example, for the Type 1 repeats, the B2-10, E6-2, and C2-4 element pattern proteins with variable numbers of repeats resulted in three different gap insertions in all of the other protein sequences (**Figure 4A**, highlighted in aqua and indicated with arrows). When a phylogenetic tree was constructed from the GUIDANCE2-PRANK alignment using the neighbor joining method, which was the same tree construction program employed in NJGT-PRANK, it showed that this altered alignment was based on the variable number of Type 1 repeats in the proteins (**Figure 4B**). As in the tree generated by NJGT-PRANK (**Figure 1A**), the B2-10 protein clustered with other B element pattern proteins, while the C2-4 protein clustered with the C5-4 protein, and the E6-2 protein clustered with the E3-2 protein (**Figure 4B**, boxed clades). The B2-10, C2-4, and E6-2 proteins all have three Type 1 repeats whereas the C5-4, E3-2, and the other B pattern proteins have two. Because the proteins with three Type 1 repeats clustered with proteins with two Type 1 repeats, R1.3 was not aligned with other Type 1 repeats and was the basis for the low GUIDANCE score (**Figure 4A**, blue bar graph) indicating low confidence for the placement of this repeat. It was noteworthy that while the GUIDANCE2-PRANK alignment did not align these three Type 1 repeats together, NJGT-PRANK only failed to align B2-10 even though the placement of these three proteins on the neighbor joining trees was identical (compare **Figures 1A**, **4B**). However, this variation in the Type 1 alignment was not observed in the alternative PRANK alignments reported in the SuperMSA data. Of the 20 alternative MSAs, there were three sequences for which all regions were aligned and the edges of R1.1 were properly identified, although this was not the case for R1.2 to R1.4 (data not shown). Although the alignments from these several approaches were not identical, the outcomes suggested agreement for the numbers and edges of Type 1 repeats relative to previous reports (22, 29) and that there may not be alternative alignments for Type 1 repeat region in the SpTrf proteins.

The repeat-based alignment is established by positions (or columns) that result in elements that are defined by gaps (22). Therefore, results from GUIDANCE analysis of the GUIDANCE2-PRANK alignment showed numerous regions where the scores were low (< 0.93). The exception was a few regions where the L proteins aligned with each other and where a gap was inserted into the S proteins (**Figure 4A**). In general, the average GUIDANCE score for the Type 1 repeat region showed low confidence of 0.86 (**Figure 4A**, green box). Other regions with low confidence included L-Er7 and S-Er8, which were aligned together as in the NJGT-PRANK alignments described above, with a GUIDANCE score of 0.92 (**Figure 4A**, red box). The alignment with low scores also included Er9 and Er10 (scores of 0.89 and 0.79, respectively) (**Figure 4A**, black box), and the region of Er11 to Er22 (score of 0.79) (**Figure 4A**, blue box). However, unlike the results for the C element pattern proteins (**Figure 3**), the GUIDANCE2-PRANK alignment of Er11 to Er22 corresponded with the same elements in the other S proteins resulting in a GUIDANCE score of 0.98 (**Figure 4C**, black box).

To determine whether there were other alternative alignments, GUIDANCE2 was also used to evaluate a ClustalW alignment (GUIDANCE2-ClustalW) and the confidence scores were compared to those for the GUIDANCE2-PRANK alignment. The GUIDANCE2-ClustalW alignment had no regions of no confidence but was composed of long regions of low or variable confidence (**Figure 4D**). This result was unlike the GUIDANCE2-PRANK alignment that had regions of low to no confidence but was generally composed of blocks of regions with high confidence (**Figure 4A**). The regions of low or variable confidence in the GUIDANCE2-ClustalW alignment was the Type 1 repeat region with an average GUIDANCE score of 0.75 (**Figure 4D**, green box), L-Er7 and S-Er8 (score of 0.92) (**Figure 4D**, red box), Er10 and Er11 (score of 0.62) (**Figure 4D**, black box), Er22 to Er25 (score of 0.64) (**Figure 4D**, yellow box), and a large region encompassing S-Er14, S-Er16, S-Er17, and S-Er21 that aligned with L-Er11 and L-Er12 (score of 0.71) (**Figure 4D**, purple box). Overall, the GUIDANCE2-ClustalW alignment had an overall score of 0.894 that included many columns of low scores and only about half with scores of high confidence (score of ≥ 0.93). In contrast, the GUIDANCE2-PRANK alignment had an overall score of 0.913 with about 70% of the columns with scores of ≥ 0.93, verifying that the PRANK alignment aligned more regions of similar sequences that required gap insertions. These two computational approaches resulted in SpTrf alignments that were quite different with variations in the level of GUIDANCE confidence scores for various regions and different columns (**Figures 4A, D**).

## Discussion

The *SpTrf* genes, which encode the protein sequences that are aligned and evaluated here, have a mosaic pattern of sequences with different types and numbers of internal repeats, have required manual alignments to compare and evaluate the full-length sequences. These manual alignments have been essential to identify elements and the element patterns of these proteins based on the positions of large gaps (11–13, 22). Repeat Types 1 to 5 were identified based on searches with Megalign [DNASTAR (11)] and the Type 6 repeat was identified by Buckley et al. (29). Some repeats are present at least twice in the SpTrf proteins and two types are present up to four times. Because of these characteristics, aligning these sequences using MSA algorithms without manual editing has been limited. SpTrf alignments have been used to classify new SpTrf sequences to element pattern, are key to further analysis of sequence comparisons among these proteins, have been used to understand the *SpTrf* gene family diversity and evolution (27), in addition to investigations of homology structuring, and a number of other genomic analyses [reviewed in (45)]. To determine whether additional optimal alignments of the SpTrf protein sequences can be identified based on computational approaches without manual adjustments, PRANK was employed for this task because it is an algorithm designed to align sequences that require large gap insertions. The resulting alignments from several approaches employing PRANK show mixed qualities within and between alignments. Certain regions of the SpTrf sequences appear to align well, such as Er10, whereas in other alignments, the order of some aligned repeats are shifted in which S-Er10 (R6.1) is aligned with L-Er18 (R6.2). Although the alignments from PRANK show more regions of matched amino acids compared to the ClustalW alignment, they are not as parsimonious as the manual repeat-based alignment.

A correspondence between the edges of the repeats and the edges of the elements is not always maintained, which is noteworthy for the tandem Type 1 repeats that are present in different numbers among the proteins (**Figures 2**, **S2**, **S3**) (29). However, the cDNA-based alignment (13) and a preliminary alignment of partial sequences (11) did not align edges of repeats and elements. Although the basis for producing the repeat-based alignment was to make the edges of repeats and elements correspond, the computational approaches evaluated here resulted in alternative alignments of the Type 1 repeats. This suggested that multiple alignments for this region are feasible and that one alignment approach should not be deemed as the best option. Previous studies have speculated that the Type 1 repeats in the L proteins may have undergone separate evolutionary histories from the Type 1 repeats in the S proteins (29), and the failure to align L-R1.1 with S-R1.1 computationally in PRANK appears to support this hypothesis. Similarly, the C element pattern proteins, in particular, show mismatches with all other proteins in the region of elements Er11 to Er22. This outcome may also be based on differences in the theoretical evolutionary history of the *SpTrf* family in which genes with different element patterns may have been derived from different intermediate ancestral genes [see Figure 9 in (27)].

The usage of guide trees of the SpTrf sequences for PRANK alignments can be a hindrance when the tree has many short branch lengths, many nodes with bootstrap values of well below 50, and clades composed of sequences with different element patterns (*e*.*g*., B2-10 in **Figure 1**). Consequently, optimal alignments require guide trees that are generated through computational approaches, which becomes a circular problem because a robust alignment is required to construct an accurate tree, and an accurate tree is required to construct a robust alignment. However, the PRANK algorithm with input from the SuperMSA of 20 concatenated alignments, as provided by GUIDANCE2, is a means to evaluate the output and identify optimal and suboptimal regions in an alignment. This approach is an improvement for computational alignments of the SpTrf sequences and is based on sufficient variation among the ~400 alignments in the SuperMSA that can be employed in addition to results from bootstrap iterations. This approach identifies edges of certain repeats that are in agreement with the sequences of the several types of repeats reported by Nair et al. (11) and Buckley et al. (29). However, while GUIDANCE scores alone cannot be used to determine which alignment is optimal, these scores in conjunction with a visual analysis of PRANK alignments is a preferable approach for analysis of the SpTrf sequences.

The manual cDNA-based alignment (13, 22) has not been generally employed for evaluating the SpTrf sequences compared to the repeat-based alignment. This is because it does not correlate the edges of the repeats with the edges of the elements and because of a large gap in the interspersed repeat region of the proteins (**Figure 3E**). However, all alignments reported here also show large gaps in the interspersed repeat region of the proteins that are different in both size and location (**Table 1**). The greatest effect on the length of the alignment is in the cDNA-based alignment and least effect in the repeat-based alignment, with the gap effect from computational approaches showing lengths that are in between. Although this simple analysis does not capture all benefits and drawbacks of computational approaches, it suggests that large gaps in the interspersed region may be an essential aspect of these alignments and that their location and size are unrelated to alignment quality.

**Table 1:** Different approaches to align the SpTrf proteins result in different alignments.

Alignment approach	Method	Position of large gaps^1^	Alignment length^2^	Figure reference
Repeat-based	Manual	None	507	**Figure S1**, based on (22)
ClustalW	Computational	257-345	473	**Figure S4**
NJGT-PRANK	Computational	354-392, 402-440	600	**Figure S2**
MLGT-PRANK	Computational	254-345	590	**Figure S3**
GUIDANCE2-PRANK	Computational	430-570	655	**Figure 4A**
GUIDANCE2-ClustalW, NJGT^3^	Computational	260-343	480	**Figure 4D**
cDNA alignment	Manual	390-528	595	**Figure S2** in (12)

^1^All large gaps are positioned in the Type 2 to Type 6 repeats that are interspersed in the C terminal half of the proteins.

^2^Alignment length is based on amino acid sequence.

^3^A neighbor joining guidance tree was provided for this analysis.

## Conclusion

The *SpTrf* gene family in the purple sea urchin is composed of small genes that are tightly clustered, have repeats in the coding region of the second of two exons, and are surrounded by short tandem repeats. This general structure has been proposed as imparting genomic instability for the regions of the sea urchin genome that harbor these genes (26, 27). This has been speculated to be a fitness benefit that generates sequence diversity among the *SpTrf* genes through deletions, duplications, and gene conversion. The improved species benefit of SpTrf protein sequence variability is based on their immune responsiveness that applies to the health and survival of sea urchins by providing protection from a wide variety of marine microbial pathogens (23, 25). An important aspect of the analysis of the encoded proteins is based on alignments to identify repeats, elements, and the element pattern. Our approach has been to determine whether these proteins with a mosaic of elements and multiple repeats of different types can be aligned in more than the two manual alignments that have been reported to date (13, 22). The manual repeat-based alignment appears to be the most parsimonious, however, the computational approaches suggest alternative alignment options for specific regions of these proteins.

## Data availability statement

The original contributions presented in the study are included in the article/**Supplementary Material**. Further inquiries can be directed to the corresponding author.

## Author contributions

MABH conceived the research, carried out the study and the data analyses, and wrote and edited the manuscript; LCS provided the funding for the study and edited the manuscript. Both authors approved the submitted manuscript.

## Funding

This work was supported by funding from the National Science Foundation (IOS-1146124, IOS-1550474, IOS-1855747) to LCS and awards from the Wilber V. Harlan Trust through the Department of Biological Sciences and a Dissertation Fellowship from the Columbian College of Arts and Sciences at GWU to MABH.

## Acknowledgments

The authors are grateful to Helen Dooley for recommending the PRANK program for use with the SpTrf sequences. Leon Grayfer and Damien O’Halloran provided helpful critiques to improve the manuscript.

## Conflict of interest

The authors declare that the research was conducted in the absence of any commercial or financial relationships that could be construed as a potential conflict of interest.

## Publisher’s note

All claims expressed in this article are solely those of the authors and do not necessarily represent those of their affiliated organizations, or those of the publisher, the editors and the reviewers. Any product that may be evaluated in this article, or claim that may be made by its manufacturer, is not guaranteed or endorsed by the publisher.

## Abbreviations

Er, elements defined by the repeat-based alignment; L-Er, elements in the long proteins; S-Er, elements in the short proteins; R, repeat, *e.g*., R1.2, repeat Type 1 and the second Type 1 repeat in the sequence; L-R, repeat in a long protein; S-R, repeat in a short protein.
