# Supplementary material for: The complex set of internal repeats in SpTransformer protein sequences result in multiple but limited alternative alignments
Source: Front Immunol. 2022 Oct 18;13:1000177. doi: 10.3389/fimmu.2022.1000177 (PMC9623053; doi:10.3389/fimmu.2022.1000177)
Supplement: Supplementary file 1 [file DataSheet_1.pdf]

Supplementary Figures

The repeats in SpTransformer sequences result in multiple but limited alternative alignments

Megan A. Barela Hudgell, L. Courtney Smith

Figure S1 | The repeat-based alignment illustrates the elements for a representative set of deduced SpTrf protein sequences.

Figure S2 | The NJGT-PRANK alignment of representative deduced SpTrf protein sequences increases the number of gaps in the alignment.

Figure S3 | The MLGT-PRANK alignment of representative SpTrf protein sequences with a provided maximum likelihood guide tree results in regions of misaligned sequence.

Figure S4 | The ClustalW alignment without manual correction includes a non-matching region of the deduced SpTrf proteins.

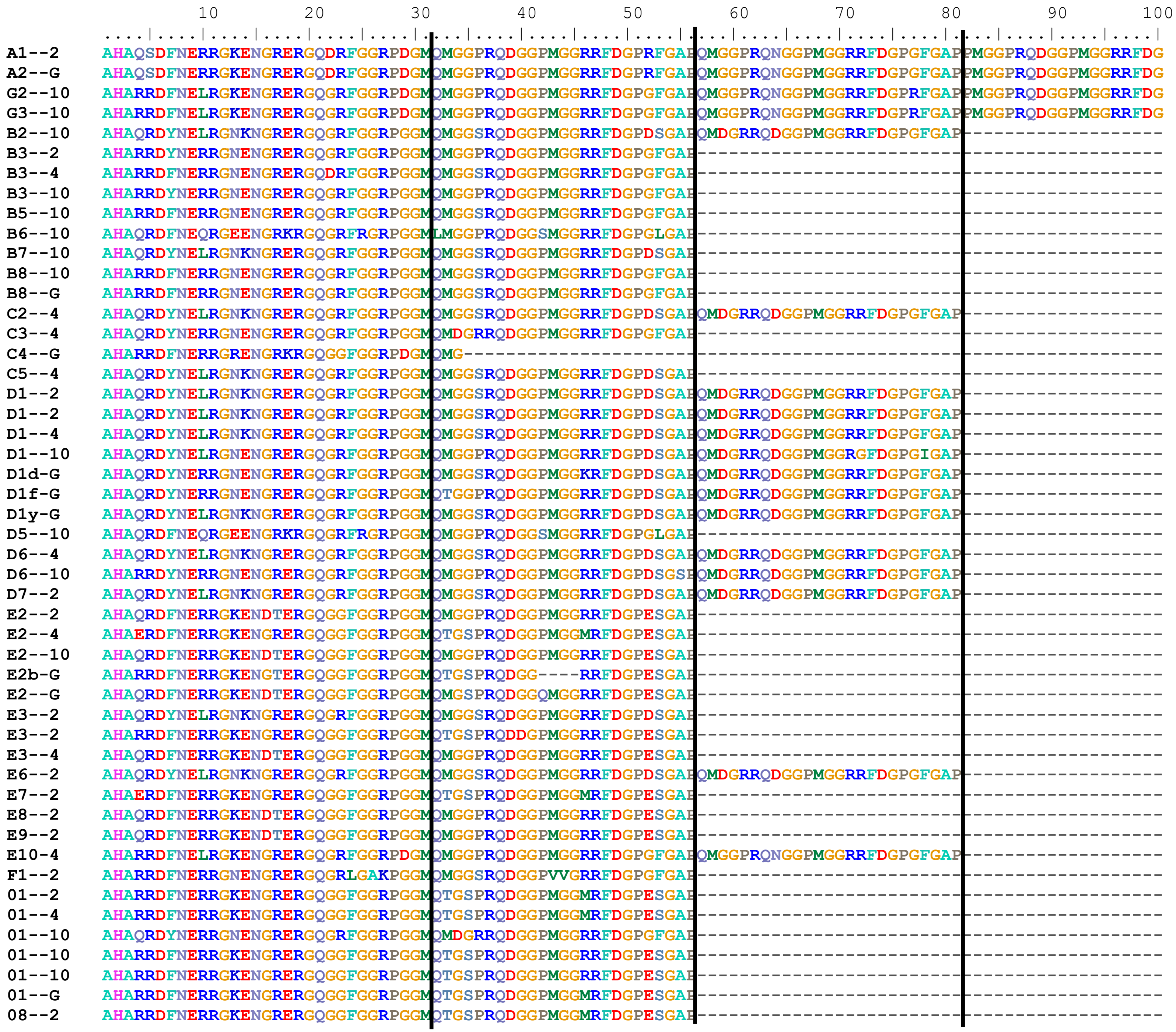
10 20 30 40 50 60 70 80 90 100
A1--2
A2--G
G2--10
G3--10
B2--10
B3--2
B3--4
B3--10
B5--10
B6--10
B7--10
B8--10
B8--G
C2--4
C3--4
C4--G
C5--4
D1--2
D1--2
D1--4
D1--10
D1d-G
D1f-G
D1y-G
D5--10
D6--4
D6--10
D7--2
E2--2
E2--4
E2--10
E2b-G
E2--G
E3--2
E3--2
E3--4
E6--2
E7--2
E8--2
E9--2
E10-4
F1--2
01--2
01--4
01--10
01--10
01--10
01--G
08--2

```
                 110       120       130       140       150       160       170       180       190       200
        ....|....|....|....|....|....|....|....|....|....|....|....|....|....|....|....|....|....|....|....|
A1--2   PGFGAEQMGGPRQNGGPMGGRRFDGPGFGGSRPDGAGGRPFFGEGGRRGDGEEETDAAQQIGDGLGGPGRCDGPGHGHYGHHQGAGRPFFGNPPPFNPEQ
A2--G   PGFGAEQMGGPRQNGGPMGGRRFDGPRFGGSRPDGAGGRPFFGEGGRRGDGEEETDAARQIGPGR-----FDGPGHGHYGHHQGAGRPFFGNPPPFNPEQ
G2--10  PGFGAEQMGGPRQNGGPMGGRRFDGPGFGGSRPDGAGGRPFFGEGSRRGDGEEETDAARQIGDGLGGPGRFDGPGHGHYGHHQGAGRPFFGNPPPFNPEQ
G3--10  PGFGAEQMGGPRQNGGPMGGRRFDGPGFGGSRPDGAGGRPFFGEGSRRGDGEEETDAARQIGDGLGGPGRFDGPGHGHYGHHQGAGRPFFGNPPPFNPEQ
B2--10  ------EMDGRRQNGGPMGGRRFDGPGFGGSRPDGAGGRPFFGQGGRRGDGEEETDAAQQIGDGLGGPGQFDGHGRRHHG--------------------
B3--2   ------QMDGRRQNGGPMGGRRFDGPRFGGSRPDGAGGRPFFGQGGRRGDGEEETDAAQQIGDGLGGRGQFDGHGRRHHG--------------------
B3--4   ------HMDGRRQNGGPMGGRRFDGPGFGGSRPDGAGGRPFFGQGGRRGDGEEETDAAQQIGDGLGGRGRFDGHGRRHHG--------------------
B3--10  ------QMDGRRQNGGPMGGRRFDGPRFGGSRPDGAGGRPFFGQGGRRGDGEEETDAAQQIGDGLGGRGQFDGHGRRHHG--------------------
B5--10  ------HMDGRRQNGGPMGGRRFDGPGFGGSRPDGAGGRPFFGQGGRRGDGEEETDAAQQIGDGLGGRGQFDGHGRRHHG--------------------
B6--10  ------QMDGRRQNGGPMGGRRFDGPGFGGSRPDGAGGRPFFGQGGRRGDGEEETDAAQQIGDGLGGPGQFDGHGRRRHG--------------------
B7--10  ------QMDGRRQNGGPMGGRRFDGPGFGGSRPDGAGGRPFFGQGGRRGDGEEETDAAQQIGDGLGGSDRFDGLRRGHHG--------------------
B8--10  ------HMDGRRQNGGPMGGRRFDGPGFGGSRPDGAGGRPFFGQGGRRGDGEEETDAAQQIGDGLGGRGQFDGHGRRHHG--------------------
B8--G   ------HMDGRRQNGGPMGGRRFDGPGFGGSRPDGAGGRPFFGQGGRRGDGEEETDASQQIGDGLGGRGQFDGHGRRHHG--------------------
C2--4   ------EMDGRRQNGGPMGGRRFDGPGFGGSRPDGAGGRPFFGQGGRRGDGEEETDAAQQIGDGLGGSDRFDGPRRGHHG--------------------
C3--4   ------QMDGRRQNGGPMGGRRFDGPGFGGSRPDGAGGRPFFGQGGRRGDGEEETDAAQQIGDGLGGSDRFDGPRRGHHG--------------------
C4--G   -------------------GRRFDGPGFGGSRPDGAG-RPFFGQGGKRGDGEEETDAAQQIGDGLGGSDRFDGPRRGHHG--------------------
C5--4   ------QMDGRRQNGGPMGGRRFDGPGFGGSRPDGAGGRPFFGQGGRRGDGEEETDAAQQIGDGLGGSDRFDGPRRGHHG--------------------
D1--2   ------EMDGRRQNGGPMGGRRFDGPGFGGSRPDGAGGRPFFGQGGRRGDGEEETDAAQQIGDGLGGPGQFDGPGRRHHG--------------------
D1--2   ------EMDGRRQNGGPMGGRRFDGPGFGGSRPVGAGGRLFFGQGGRRGDEEEETDAAQQIGDGLGGPGQFDGPGRRHHG--------------------
D1--4   ------EMDGRRQNGGPMGGRRFDGPGFGGSRPDGAGGRPFFGQGGRRGDGEEETDAAQQIGDGLGGPGQFDGPGRRHHG--------------------
D1--10  ------EMDGTEKNGGPMGGRELDGPGFGGSRPDGGGGRPFFGQGGRRGDGEEETDAAQQIGDGLGGPGQFDGPGRRHHG--------------------
D1d-G   ------EMDGRRQNGGPMGGRRFDGPGFGGSRPVGAGGRPFFGQGGRRGDGEEETDAAQQIGDGLGGPGQFDGPGRRHHG--------------------
D1f-G   ------EMDGRRQNGGPMGGRRFDGPGFGGSRPVGAGGRPVFGQGGRRGDGEEETDAAQQIGDGLGGPGQFDGPGRRHHG--------------------
D1y-G   ------EMDGRRQNGGPMGGRRFDGPGFGGSRPDGAGGRPFFGQGGRRGDGEEETDAAQQIGDGLGGPGQFDGPGRRHHG--------------------
D5--10  ------QMDGRRQNGGPMGGRRFDGPGFGGSRPDGAGGRPFFGQGGRRGDGEEETDAAQQIGDGLGGPGQFDGPGRRHHG--------------------
D6--4   ------EMDGRRQNGGPMGGRRFDGPGFGGSRPDGAGGRPFFGQGGRRGDGEEETDAAQQIGDGLGGPGQFDGPGRRHHG--------------------
D6--10  ------EMDGRRQNGGPMGGRRFDGPGFGGSRPDGAGGRPFFGQGGRRGDGEEETDAAQQIGDGLGGPGQFDGPGRRHHG--------------------
D7--2   ------EMDGRRQNGGPMGGRRFDGPGFGGSRPDGAGGRPFFGQGGRRGDGEEETDAAQQIGDGLGGPGQFDGPGRRHHG--------------------
E2--2   ------QMEGRRQNGGPMGGRRFDGPRFGGSRPDGAGGRPFFGQGGRRGDGEEETDAAQQIGDGLGGRDQFDGHGRGHHG--------------------
E2--4   ------QMDGRRQNGGPMGGRRFDGPRFGGSRPDGAGGRPFFGQGGRRGDGKEETDAAPLIGDGLGGRGQFDGHGRGHHG--------------------
E2--10  ------QMEGRRQNGGPMGGRRFDGPRFGGSRPDGAGGRPFFGQGGRRGDGEEETDDDQKMGDGPGGRGQFDGHGRGHHG--------------------
E2b-G   ------QMDGRRQNGGPMGGRRFDGPRFGGSRPDGAGGRPFFGQGGRRGDGEEETDAAQQIGDGLGGSGQFDGPRRGHHG--------------------
E2--G   ------QMEGRRQNGGPMGGRRFDGPRFGGSRPDGAGGRPFFGQGGRRGDGEEETDAAQQIGDGLGGRGQFDGHGRGHHG--------------------
E3--2   ------QMDGRRQDGGPMGGRRFDGPGFGGSRPDGAGGRPFFGQGGRRGDGEEETDAAQQMGDGLGGRGQFDGPRRGHHG--------------------
E3--2   ------QMDGRRQNGGPMGGRRFDGPRFGGSRPDGAGGRPFFGQGGRRGDGEEETDAAQQIGDGLGGSGQFDGPRRGHHG--------------------
E3--4   ------QMEGRRQNGGPMGGRRFDGPGFGGSRPDGAGGRPFFGPGGRRGDGEEETDAALLIGDVLGGRGQFDGPRRGHHG--------------------
E6--2   ------EMDGRRQNGGPMGGRRFDGPGFGGSRPVGAGGRPFFGQGGRRGDGEEETDAAQQIGDGLGGRGQFDGHGRGHHG--------------------
E7--2   ------QMDGRRQNGGPMGGRRFDGPRFGGSRPDGAGGRPFFGQGGRRGDGKEETDAAQQIGDGLGGRGQFDGHGRGHHG--------------------
E8--2   ------QMDGRRQNGGPMGGRRFDGPRFGGSRPDGAGGRPFFGQGGRRGDGEEETDAAQQIGDGPGGRGQFDGHGRGHHG--------------------
E9--2   ------QMDGRRQNGGPMGGRRFDGPRFGGSRPDGAGGRPFFGQGGRRGDGEEETDAAQQIGDGPGGRGQFDGHGRGHHG--------------------
E10-4   ------QMGGPRQNGGPMGGRRFDGPGFGGSRPDCAGGRPFFGEGGRRGDGEEETDAARQIGDGLGGPGQFDGPRRGHHG--------------------
F1--2   ------HMDGRRQNGGPMGGRRFDGPGFGGSRPDGAGGRPFFGQGGRRGDGEEETDAAQQIGDGLGGRGQFDGHGRRHHG--------------------
01--2   ------QMDGRRQNGGPMGGRRFDGPRFGGSRPDGAGGRPFFGQGGRRGDGEEETDAAQQIGDGLGGPGQFDGPGRRHHG--------------------
01--4   ------QMDGRRQNGGPMGGRRFDGPGFGGSRPDGAGGRPFFGQGGRRGDGEEETDAAQQIGDGLGGPGQFDGPGRRHHG--------------------
01--10  ------QMDGRRQNGGPMGGRRFDGPGFGGSRPDGAGGRPFFGQGGRRGDGEEETDAAQQIGDGLGGPGQFDGPGRRHHG--------------------
01--10  ------QMDGRRQNGGPMGGRRFDGPGFGGSRPDGAGGRPFFGQGGRRGDGEEETDAAQQIGDGLGGPGQFDGPGRRHHG--------------------
01--10  ------QMDGRRQNGGPMGGRRFDGPRFGGSRPDGAGGRPFFGQGGRRDDGEEETDAAQQIGDGLGGPGQFDGPGRRHHG--------------------
01--G   ------QMDGRRQNGGPMGGRRFDGPGFGGSRPDGAGGRPFFGQGGRRGDGEEETDAAQQIGDGLGGPGQFDGPGRRHHG--------------------
08--2   ------QMDGRRQNGGPMGGRRFDGPRFGGSRPDGAGGRPFFGQGGRRGDGEEETDAAQQIGDGLGGPGQFDGPGRRHHG--------------------
```

```
                210       220       230       240       250       260       270       280       290       300
        ....|....|....|....|....|....|....|....|....|....|....|....|....|....|....|....|....|....|....|....|
A1--2   EP-----------------RNDNSEEDGRHHRHH---DRHHAHHGHHGHHEHHHQ-HH----NHTEGHQDHDRPMFEMRPFRFNPLGRKPFGDHPFGRRN
A2--G   EP-----------------RNDSSEEDGRHHRHH---DRHHAHHGHHGHHEHHHQ-HH----NHTEGHQDHDRPMFEMRPFRFNPLGRKPFGDHPFGRRN
G2--10  EP-----------------RNDSSEEDGRHHRHH---DRHHAHHGHHGHHEHH-N-HH----NHTEGHQDHDRPMFEMRPFRFNPLGRKPFGDHPFGRRN
G3--10  EP-----------------RNDSSEEDGRHHRHH---DRHHAHHGHHGHHEHH-N-HH----NHTEGHQ-------------------------------
B2--10  --HRQGPPQDRPEEQPFGQRNYSNEEDGRPHPHH---HGHHGHHGH--HH--R---HH----NQTEGHQ-------------------------------
B3--2   --HRQGPPQDRPEEQPFGQRNYSNEEDGRPHPHH---HGHHGHHGH--HH--R---HH----NQTEGHQ-------------------------------
B3--4   --HRQGPPQDRPEEQPFGQRNERNEEDGRPHPHH---HGHHGHHGH--HH--R---HH----NQTEGHQ-------------------------------
B3--10  --HRQGPPQDRPEEQPFGQRNYSNEEDGRPHPHH---HGHHGHHGH--HH--R---HH----NQTEGHQ-------------------------------
B5--10  --HRQGPPQDRPEEQPFGQRNERNEEDSRPHPHH---HRHHGHHDH--HH--R---HH----NQTEGHQ-------------------------------
B6--10  --HRQGPPQDRSEEQPFGQRNESSEEDGRPHPHH---HRHHDHHD--RHH--R---HH----NHTEGHQ-------------------------------
B7--10  --HRQGPPQDRPEEQPFGQRNESSEEDGRPHPHH---HRHHDHHD--RHH--R---HH----NHTEGHQ-------------------------------
B8--10  --HRQGPPQDRPEEQPFGQRNERNEEDSRPHPHH---HRHHGHHDH--HH--R---HH----NQTEGHQ-------------------------------
B8--G   --HRQGPPQDRPEEQPFGQRNERNEEDGRPHPHH------HGHHGHQGHHH----RHH----NQTEGHQ-------------------------------
C2--4   --HRQGPPQDRPEEQPFGQRNYSSEEDGRPHPHH---HRHHGHHRH--H---H---HH----NQTEGHQ-------------------------------
C3--4   --HRQGPPQDRPEEQPFGQRNYSSEEDGRPHPHH---HRHHGHHRH--H---H---HH----NQTEGHQ-------------------------------
C4--G   --HRQGPPQDRPEEQPFGQRNESSEEDGRPHPHHHGHHRHH-------RHHHHH--------NQTEGHQ-------------------------------
C5--4   --HRQGPPQDRPEEQPFGQRNYSSEEDGRPHPHH---HRHHGHHRH--H---H---HH----NQTEGHQ-------------------------------
D1--2   --HRQGHPQDQAEEQPFGQRNESSEEDGRPHPHH------HRHHGH--HH--R---HH----NHTEGHQ-------------------------------
D1--2   --HRQGHPQDQAEEQPFGQRNESSEEDGRPHPHH------HRHHGH--HH--R---HH----NHTEGHQ-------------------------------
D1--4   --HRQGHPQDQAEEQPFGQRNESSEEDGRPHPHH------HRHHGH--HH--R---HH----NHTEGHQ-------------------------------
D1--10  --HRRGHPQDQAEEQPFGQRNESSEEDGRPHPHH------HRHHGH--HH--R---HH----NHTEGHQ-------------------------------
D1d-G   --HRQGHPQDQAEEQPFGQRNKSSEEDGRPHP---------HHHRDHGHHH----RHH----NHTEGHQ-------------------------------
D1f-G   --HRQGHPQDQAEEQPFGQRNESSEEDGRPHPHH---HRHH------GHHH----RHH----NHTEGHQ-------------------------------
D1y-G   --HRQGHPQDQAEEQPFGQRNESSEEDGRPHP---------HHHRHHGHHHR----HH----NHTEGHQ-------------------------------
D5--10  --HRRGHPQDQAEEQPFGQRNESSEEDGRPHPHH------HRHHGH--HH--R---HH----NHTEGHQ-------------------------------
D6--4   --HRQGHPQDQAEEQPFGQRNESSEEDGRPHPHH------HRHHGH--HH--R---HH----NHTEGHQ-------------------------------
D6--10  --HRQGHPQDQAEEQPFGQRNESSEEDGRPHPHH------HRHHGH--HH--P---HH----NHTEGHQ-------------------------------
D7--2   --HRQGHPQDQAEEQPFGQRNESSEEDGRPHPHH------HRHHGH--HH--R---HH----NHTEGHQ-------------------------------
E2--2   --HRQGPPQDRPEEQPFGQRNESSDEDGRPHPRH------HG--RH--HQ--H---HHR---NHTEGHQ-------------------------------
E2--4   --HRQGPPQDRPEEQPFGQRNESSDEDGRPHPRH------HG--RH--HQ--H---HHR---NHTEGHQ-------------------------------
E2--10  --HRQGPPQDRPEEQPFGQRNESSDEDGRPHPRH------HG--RH--HQ-H----HHR---NRTEGHQ-------------------------------
E2b-G   --HRQGPPQDRPEEQPFGQRNESSDEDGRPHP------------RHHGRHHQH---HHR---NHTEGHQ-------------------------------
E2--G   --HRQGPPQDRPEEQPFGQRNESSDEDGRPHP------------RHHGRHHQ---HHHR---NHTEGHQ-------------------------------
E3--2   --HRQGPPQDRPEEQPFGQRNESSDEDGRPHPRH------HG--RH--HQ--H---HHR---NHTEGHQ-------------------------------
E3--2   --HRQGPPQDRPEEQPFGQRNESSDEDGRPHPRH------HG--RH--HQ--H---HHR---NHTEGHQ-------------------------------
E3--4   --HRQGPPQDRPEEQPFGQRNESSDEDGRPHPRH------HG--RH--HQ--H---HHR---NHTEGHQ-------------------------------
E6--2   --HRQGPPQDRPEEQPFGQRNESSDEDGRPHPRH------HG--RH--HQ--H---HHR---NHTEGHQ-------------------------------
E7--2   --HRQGPPQDRPEEQPFGQRNESSDEDGRPHPRH------HG--RH--HQ--H---HHR---NHTEGHQ-------------------------------
E8--2   --HRQGPPQDRPEEQPFGQRNESSDEDGRPHPRH------HG--RH--HQ-H----HHR---NRTEGHQ-------------------------------
E9--2   --HRQGPPQDRPEEQPFGQRNESSDEDGRPHPRH------HG--RH--HQ-H----HHR---NRTEGHQ-------------------------------
E10-4   --HRQGPPQDRPEEQPFGQRNESSDEDGRPHPRH------HG--RH--HQ--H---HHR---NHTEGHQ-------------------------------
F1--2   --HRQGPPQDRPEEQPFGQRNERNEEDGRPHPHH------HG---H--HH--R---HH----NQTEGHQ-------------------------------
01--2   ----------------------------------------------------------------------------------------------------
01--4   ----------------------------------------------------------------------------------------------------
01--10  ----------------------------------------------------------------------------------------------------
01--10  ----------------------------------------------------------------------------------------------------
01--10  ----------------------------------------------------------------------------------------------------
01--G   ----------------------------------------------------------------------------------------------------
08--2   ----------------------------------------------------------------------------------------------------
```

```
               310       320       330       340       350       360       370       380       390       400
A1--2    HTEGHQGHNETGDHPHRHHSKTVDGDQDT---GHHGHHGHHEHHHHQHDHREGHQDHDRPMFGMRPFRFNPFGRKPFGDHPFGRRNHTEGHQGHNETG
A2--G    HTEGHQGHNETGDHPHRHHSKNVDGDQDT---GHHGHHGHHEHHHHQHDHREGHQDHDRPMFEMRPFRFNPLGRKPFGDHPFGRRNHTEGHQGHNETG
G2--10   HTEGHQGHNETGDHPNRHHSKTVDGDQDT---GHHGHHGHHEHHHHQHNHREGHQDHDRPMFGMRPFRFNPFGRKPFGDHPFGRRNHTEGHQGHNETG
G3--10   --------------------------------------------------------------------------------------------------
B2--10   ------GHNETGDQ---------DQDKPNDT---------------------------------RPFRFNHFG----------RRNHTEGHQGHNETG
B3--2    ------GHNETGDQ---------DQDKPNDT---------------------------------RPFRFNHFG----------RRNHTEGHQGHNETG
B3--4    ------GHNETGDQ---------DQDKPNDT---------------------------------RPFRFNHFG----------RRNHTEGHQGHNETG
B3--10   ------GHNETGDQ---------DQDKPNDT---------------------------------RPFRFNHFG----------RRNHTEGHQGHNETG
B5--10   ------GHNETGDQ---------DQDKPNDT---------------------------------RPFRFNHFG----------RRNHTEGHQGHNETG
B6--10   ------GHNETGDH------------------------------------------------------------------------------------
B7--10   ------GHNETGDH------------------------------------------------------------------------------------
B8--10   ------GHNETGDQ---------DQDKPNDT---------------------------------RPFRFNHFG----------RRNHTEGHQGHNETG
B8--G    ------GHNETGDQ---------DQDKPNDT-------------------------------------------RPFRFNHFGRRNHTEGHQGHNETG
C2--4    ------GHSETGDQ---------DQDKPIDT---------------------------------RPFRFNHFGRKPFGDRPFGRRNHTEGHQGHNETG
C3--4    ------GHNETGDQ---------DQDKPIDT---------------------------------RPFRFNHFGRKPFGDRPFGRRNHTEGHQGHNETG
C4--G    ------GHNDTGDQ---------DQDKPNDT---------------------------------RPFRFNHFGRKPFGDRPFGRRNHTEGHQGHNETG
C5--4    ------GHNETGDQ---------DQDKPIDT-------------------------------------------------------------------
D1--2    ------GHNETGDQ---------DQDKLHDT---------------------------------RPFRYNHFGRKPFGDRPFGRRNHTEGHQGHNETG
D1--2    ------GHNETGDQ---------DQDKLHDT---------------------------------RPFRYNHFGRKPFGDRPFGRRNHTEGHQGHNETG
D1--4    ------GHNETGDQ---------DQDKLHDT---------------------------------RPFRYNHFGRKPFGDRPFGRRNHTEGHQGHNETG
D1--10   ------GHNETGDQ---------DQDKLNDT---------------------------------RPFRYNYFGRKPFGDRTFGRRNHTEGHQGHNETG
D1d-G    ------GHNETGDQ---------DQDKLHDT---------------------------------RPFRYNHFGRKPFGDRPFGRRNHTEGHQGHNETG
D1f-G    ------GHNETGDQ---------DQDKLHDT---------------------------------RPFRYNHFGRKPFGDRPFGRRNHTEGHQGHNETG
D1y-G    ------GHNETGDQ---------DQDKLHDT---------------------------------RPFRYNHFGRKPFGDRPFGRRNHTEGHQGHNETG
D5--10   ------GHNETGDQ---------DQDKLNDT---------------------------------RPFRYNYFGRKPFGDRTFGRRNHTEGHQGHNETG
D6--4    ------GHNETGDQ---------DQDKLHDT---------------------------------RPFRYNHFGRKPFGDRPFGRRNHTEGHQGHNETG
D6--10   ------GHNETGDQ---------DQDKLHGT---------------------------------RPFRYNHFGRKPFGDRPFGRRNHTEGHQGHNETG
D7--2    ------GHNETGDQ---------DQDKLHDT---------------------------------RPFRYNHFGRKPFGDRPFGRRNHTEGHQGHNETG
E2--2    ------GHNETGDH------------------------------------------------------------------------------------
E2--4    ------GHNETGDH------------------------------------------------------------------------------------
E2--10   ------GHNETGDH------------------------------------------------------------------------------------
E2b-G    ------GHNETGDH------------------------------------------------------------------------------------
E2--G    ------GHNETGDH------------------------------------------------------------------------------------
E3--2    ------GHNETGDH------------------------------------------------------------------------------------
E3--2    ------GHNETGDH------------------------------------------------------------------------------------
E3--4    ------GHNETGDH------------------------------------------------------------------------------------
E6--2    ------GHNETGDH------------------------------------------------------------------------------------
E7--2    ------GHNETGDH------------------------------------------------------------------------------------
E8--2    ------GHNETGDH------------------------------------------------------------------------------------
E9--2    ------GHNETGDH------------------------------------------------------------------------------------
E10-4    ------GHNETGDH------------------------------------------------------------------------------------
F1--2    ------GHNETGDQ---------DQDKPNDT---------------------------------RPFRFNHFG----------RRNHTEGHQGHNETG
01--2    -------------------------------------------------------------------------RKPFGDRPFGRRNHTEGHQGHNETG
01--4    -------------------------------------------------------------------------RKPFGDRPFGRRNHTEGHQGHNETG
01--10   -------------------------------------------------------------------------RKPFGDRPFGRRNHTEGHQGHNETG
01--10   -------------------------------------------------------------------------RKPFGDRPFGRRNHTEGHQGHNETG
01--10   -------------------------------------------------------------------------RKPFGDRPFGRRNHTEGNQGHNETG
01--G    -------------------------------------------------------------------------RKPFGDRPFGRRNHTEGHQGHNETG
08--2    -------------------------------------------------------------------------RKPFGDRPFGRRNHTEGHQGHNETG
```

```
                   410       420       430       440       450       460       470       480       490       500
         ....|....|....|....|....|....|....|....|....|....|....|....|....|....|....|....|....|....|....|....|
A1--2    DHPHRHHSKTGDGDQDRPMFETRPFWVNPFGRKPFGDRPFDRRNGTEEGSPRRDGHPHPHGNRGRWGENESEEKEHPTTESVTTSSPLKVIEIAINEVDT
A2--G    DHPHRHHSKTGDGDQDRPMFETRPFWVNPFGRKPFGDRPFDRRNGTEEGSPRRDGHPHPHGNRGRWGENESEEKEHPTTESVTTSSPLKVIEIAINEVDT
G3--10   -------------DHDRPMFEMRPFRFNPFGRKPFGDRPFGRRNGTEEGSPRRDGQRRPYGNRGRWGENESEEKEHPTTESVTTSSPPEVVEIAVNEEDV
G2--10   DHPHRHHSKTGDGDQDRPMFETRPFWVNPFGRKPFGDRPFDRRNGTEEGSPRRDGHPHPHGNRRRWGENESEEKEHPTTESVTTSSPLKVIEIAINEVDT
B2--10   DHPHRHHNKTGDGDQDRPMFEMRPFWVNPFGRKPFGDRPFGRRNGTEEGSPRRDGHRRPYGNRGRWGENESEEKEHPTTESVTTSSPPEVV--AINEEDI
B3--2    DHPHRHHNKTGDGDQDRPMFETRPFWVNPFGRKPFGDRPFGRRNGTEEGSPRRDGHRRPHGNRGRWGENESEEKEHPTTESVTTSSPPEVVEIAI-----
B3--4    DHPHRHHNKTGDGDQDRPMFETRPFWVNPFGRKPFGDRPFGRRNGTEEGSPRRDGHRRPYGNRGRWGENESEEKEHPTTESVTTSSPPEVVEIAVNEEDV
B3--10   DHPHRHHNKTGDGDQDRPMFETRPFWVNPFGRKPFGDRPFGRRNGTEEGSPRRDGHRRPYGNRGRWGENESEEKEHPTTESVTTSSPPEVVEIAVNEEDV
B5--10   DHPHRHHNKTRDGDQDRPMFEMRPFRFNPFGRKPFGGRPFDRRNGTEEGSPRRDGHRRPYGNRGRWGENESEEKEHPTTESVTTSSPPEVV--AINEEDI
B6--10   --PHRHHNKTRDGDQDRPMFEMRPFRFNPFGRKPFGGRPFDRRNGTEEGSPRRDGHRRPYGNRGRWGENESEEKERPTTESVTTSSPPEVVEIAFNEEDV
B7--10   --PHRHHNKTRDGDQDRPMFEMRPFRFNPFGRKPFGGRPFDRRNGTEEGSPRRDGHRRPYGNRGRWGENESEEKERPTTESVTTSSPPEVVEIAFNEEDV
B8--10   DHPHRHHNKTGDGDQDRPMFEMRPFWVNPFGRKPFGDRPFGRRNGTEEGSPRRDGHRHPYGNRGRWGENESEEKEHPTTESVTTSSPPEVVEIAVNEEDV
B8--G    DHPHRHHNKTGDGDQDRPMFEMRPFWVNPFGRKPFGDRPFGRRNRTEEGSPRRDGHRHPYGNRGRWGENESEEKEHPTTESVTTSSPPEVVEIAVNEEDV
C2--4    DHPHRHHNKTGDGDQDRPMFESRPFRFNPFGRKPFGDRPFGRRNGTEEGSPRRDGHRRPYGNRGRWGENESEEKEHPTTESVTTSSPPEVVEIAVNEEDV
C3--4    DHPHRHHNKTGDGDQDRPMFESRPFRFNPFGRKPFGDRPFGRRNGTEEGSPRRDGHRRPYGNRGRWGENESEEKEHPTTESVTTSSPPEVVEIAFNEEDV
C4--G    DHPHRHHNQTGDGDQDRPMFEMRPFRFNPLGRKPFGDRPFGRRNGTEEGSLRRDGHRRPYGNRGRWGENESEEKEHPTTESVTTSSPPDVVEIAVNEEDV
C5--4    ----------------------RPFRFNPFGRKPFGDRPFGRRNGTEEGSPRRDGHRRPYGNRGRWGENESEEKEHPTTESVTTSSPPEVVEIAVNEEDV
D1--2    DHPHRHHNKTRDGDQDRPMFEMRPFRFNPFGRKPFGGRPFDRRNGTEEGSPRRDGHRRPYGNRGRWGENESEEKEHPTTESVTTSSPPEVV--AINEEDI
D1--2    DHPHRHHNKTRDGDQDRPMFEMRPFRFNPFGRKPFGGRPFDRRNGTEEGSPRRDGHRRPYGNRGRWGENESEEKEHPTTESVTTSSPPEVV--AINEEDI
D1--4    DHPHRHHNKTRDGDQDRPMFEMRPFRFNPFGRKPFGGRPFDRRNGTEEGSPRRDGHRRPYGNRGRWGENESEEKEHPTTESVTTSSPPEVV--AINEEDI
D1--10   DHPHRHHNKTRDGDQDRPMFEMRPFRFNPFGRKPFGGRPFDRRNGTEEGSPRRDGHRRPYGNRGRWGENESEEKEHPTTESVTTSSPPEVV--AINEEDI
D1d-G    DHPHRHHNKTRDGDQDRPMFESRPFRFNPFGRKPFGDRLFGRRNGTEEGSSRRDGHRRPYGNRGRWGENESEEKEHPTTESVTTSSPPEVVAINEEDINV
D1f-G    DHPHRHHNKTRDGDQDRPMFEMRPFRFNPLGRKPFGDRPFGRRNGTEEGSPRRDGQRRPYGNRGR*----------------------------------
D1y-G    DHPHRHHNKTRDGDQDRPMFEMRPFRFNPFRRKPFGGRPFDRRNGTEEGSPRRDGHRRPYGNRGRWGENESEEKEHPTTESVTTSSPPEVVAINEEDINV
D5--10   DHPHRHHNKTRDGDQDRPMFEMRPFRFNPFGRKPFGGRPFDRRNGTEEGSPRRDGHRRPYGNRGRWGENESEEKEHPTTESVTTSSPPEVV--AINEEDI
D6--4    DHPHRHHNKTRDGDQDRPMFEMRPFWVNPFGRKPFGDRPFGRRNGTEEGSPRRDGHRRPYGNRGRWGENESEEKEHPTTESVTTSSPPEVVEIAVNEEDV
D6--10   DHPHRHHSKTGDGDQDRPMFETRPFWVNPFGRKPFGDRPFDRRNGTEEGSPRRDGHPHPHGNRGRWGENESEEKEHPTTESVTTSSPLKVIEIAINEVDT
D7--2    DHPHRHHNKTRDGDQDRPMFEMRPFRFNPFGRKPFGGRPFDRRNGTEEGSPRRDGHRRPYGNRGRWGENESEEKEHPTTESVTTSSPPEVV--AINEEDI
E2--2    --PHRHHNKTGDGDQDRPMFEMRPFRFNPFGRKPFGDRPFGRRNGTEEGSPRRDGQRRPYGNRGRWGENESEEKEHPTTESVTTSSPP*-----------
E2--4    --PHRHHNKTGDGDQDRPMFEMRPFRFNPFGRKPFGDRPFGRRNGTEEGSPRRDGQRRPYGNRGRWGENESEEKEYPTTESVTTSSPP*-----------
E2--10   --PHRHHNKTGDGDQDRPMFEMRPFRFNPFGRKPFGDRPFGRRNGTEEGSPRRDGQRRPHGNRGRWGENESEEKEHPTTESVTTYSPP*-----------
E2b-G    --PHRHHNKTGDGDQDRPMFEMRPFRFNPFGRKPFGDRPFGRRNGTEEGSPRRDGQRRPYGNRGRWGENESEEKEHPTTESVTTSSPP*-----------
E2--G    --PHRHHNKTGDGDQDRPMFEMRPFRFNPFGRKPFGDRPFGRRNGTEEGSPRRDGQRRPYGNRGRWGENESEEKEHPTTESVTTSSPP*-----------
E3--2    --PHRHHNKTGDGDQDRPMFEMRPFRFNPFGRKPFGDRPFGRRNGTEEGSPRRDGQRRPYGNRGRWGENESEEKEHPTTESVTTSSPP*-----------
E3--2    --PHRHHNKTGDGDQDRPMFEMRPFRFNPFGRKPFGDRPFGRRNGTEEGSPRRDGQRRPYGNRGRWGENESEEKEHPTTESVTTSSPP*-----------
E3--4    --PHRHHNKTGDGDQDRPMFEMRPFRFNPFGRKPFGDRPFGRRNGTEEGSPRRDGQRRPYGNRGRWGENESEEKEHPTTESVTTSSPP*-----------
E6--2    --PHRHHNKTGDGDQDRPMFEMRPFRFNPFGRKPFGDRPFGRRNGTEEGSPRRDGQRRPYGNRGRWGENESEEKEYPTTESVTTSSPP*-----------
E7--2    --PHRHHNKTGDGDQDRPMFEMRPFRFNPFGRKPFGDRPFGRRNGTEEGSPRRDGQRRPYGNRGRWGENESEEKEHPTTESVTTSSPPEVV--AINEEDI
E8--2    --PHRHHSKTGDGDQDRPMFETRPFWVNPFGRKPFGDRPFDRRNGTEEGSPRRDGHPHPHGNRGRWGENESEEKEHPTTESVTTSSPLKVIEIAINEVDT
E9--2    --PHRHHNKTGDGDQDRPMFEMRPFRFNPFGRKPFGDRPFGRRNGTEEGSPRRDGQRRPHGNRGRWGENESEEKEHPTTESVTTYSPP*-----------
E10-4    --PHRHHNKTGDGDQDRPMFEMRPFRFNPFGRKPFGDRPFGRRNGTEEGSPRRDGQRRPYGNRGRWGENESEEKEHPTTESVTTSSPP*-----------
F1--2    DHPHRHHNKTGDRDQDRPMFEMRPFWVNPFGRKPFGDRPFGRRNGTEEGSPRRDGHRHPYGNRGRWGENESEEKEHPTTESITTSSPPEVVEIAVNEEDV
01--2    DHPHRHHNKTRDGDQDRPMFEMRPFRFNPFGRKPFGDRPFGRRNGTEEGSPRRDGQRRPYGNRGRWGENESEEKEHPTMESVTTSSPPEVVEIAINEVDT
01--4    DHPHRHHNKTRDGDQDRPMFEMRPFRFNPFGRKPFGDRPFGRRNGTEEGSPRRDGHRRPYGNRGRWGENESEEKEHPTTESVTTSSPPEVVEIAIND---
01--10   DHPHRHHNKTRDGDQDRPMFEMRPFRFNPFGRKPFGDRPFGRRNGTEEGSPRRDGQRRPHGNRGRWGENESEEKEHPTTESVTTSSPPEVVEIAIND---
01--10   DHPHRHHNKTRDGDQDRPMFEMRPFRFNPFGRKPFGDRPFGRRNGTEEGSPRRDGQRRPHGNRGRWGENESEEKEHPTTESVTTSSPPEVVEIAIND---
01--10   DHPHRHHNKTRDGDQDRPMFEMRPFRFNPFGRKPFGDRPFGRRNGTEEGSPRRDGQRRPHGNRGRWGENESEEKEHPTTESVTTSSPPEVVEIAIND---
01--G    DHPHRHHNKTRDGDQDRPMFEMRPFRFNPFGRKPFGDRPFGRRNGTEEGSPRRDGQRRPHGNRGRWGENESEEKEHPTTESVTTSSPPDVVEIAI-----
08--2    DHPHRHHNKTRDGDQDRPMFEMRPFRFNPFGRKPFGDRPFGRRNGTEEGSPRRDGQRRPYGNRGRWGENESEEKEYPTTESVTTSSPP*-----------
```

```
        ....|..
A1--2   NVVAEV*
A2--G   NVVAEV*
G3--10  NVVAEV*
G2--10  NVVAEV*
B2--10  NVVAEV*
B3--2   NDVAEV*
B3--4   NVVAEV*
B3--10  NVVAEV*
B5--10  NVVAEV*
B6--10  HVVAEV*
B7--10  NVVAEV*
B8--10  NVVAEVL
B8--G   NVVAEVY
C2--4   NVVAEV*
C3--4   NVVAEV*
C4--G   NVVAEV*
C5--4   NVVAEV*
D1--2   NVVAEV*
D1--2   NVVAEV*
D1--4   NVVAEV*
D1--10  NVVAEV*
D1d-G   VAEV*--
D1f-G   -------
D1y-G   VAEV*--
D5--10  NVVAEV*
D6--4   NVVAEV*
D6--10  NVVAEV*
D7--2   NVVAEVK
E2--2   -------
E2--4   -------
E2--10  -------
E2b-G   -------
E2--G   -------
E3--2   -------
E3--2   -------
E3--4   -------
E6--2   --VAEV*
E7--2   NVVAEV*
E8--2   NVVAEV*
E9--2   -------
E10-4   -------
F1--2   NVVAEV*
01--2   NGVAEV*
01--4   --VAEV*
01--10  --VAEV*
01--10  --VAEV*
01--10  --VAEV*
01--G   NDVAEV*
08--2   -------
```

Figure S1. The repeat-based alignment illustrates the elements for a representative set of deduced SpTrf protein sequences. The deduced amino acid sequences encoded in the second exon of the *SpTrf* genes are shown. The alignment was done manually in BioEdit (ver 7.2.5) based on a set of pre-aligned deduced sequences according to Buckley and Smith (2007). The mature SpTrf proteins were deduced from the second exon of genes sequenced from animals 2, 4, and 10 to which was added the SpTrf protein sequences from the sea urchin genome (animal G) (1–3). The protein names are located to the left and indicate the element pattern (A-G) and the sea urchin number or letter from which the sequence was obtained (2, 4, 10, G). The ruler above each alignment indicates the aa position in the alignment. Dashes (-) indicate the insertion of artificial gaps in the alignment that optimize matching sequences. Asterisks (*) indicate a stop codon. Element boarders are identified by vertical black lines. The sequences are arranged in the alignment with the L proteins at the top, the 0 proteins at the bottom, and the remaining S proteins in alphabetical order.

```
                 10        20        30        40        50        60        70        80        90       100
         ....|....|....|....|....|....|....|....|....|....|....|....|....|....|....|....|....|....|....|....|
A1--2    AHAQSDFNERRGKENGRERGQDRFGGRPDGMQMGGPR-------------------------QDGGPMGGRRFDGPRFGAPQMGGPRQNGGP--------
A2--G    AHAQSDFNERRGKENGRERGQDRFGGRPDGMQMGGPR-------------------------QDGGPMGGRRFDGPRFGAPQMGGPRQNGGP--------
G2--10   AHARRDFNELRGKENGRERGQGRFGGRPDGMQMGGPR-------------------------QDGGPMGGRRFDGPGFGAPQMGGPRQNGGP--------
G3--10   AHARRDFNELRGKENGRERGQGRFGGRPDGMQMGGPR-------------------------QDGGPMGGRRFDGPGFGAPQMGGPRQNGGP--------
B2--10   AHAQRDYNELRGNKNGRERGQGRFGGRPGGMQMGGSR-------------------------QD-------------------------GGPMGGRRFDG
B3--2    AHARRDYNERRGNENGRERGQGRFGGRPGGMQMGGPR-------------------------QD-------------------------GGP--------
B3--4    AHARRDFNERRGNENGRERGQDRFGGRPGGMQMGGSR-------------------------QD-------------------------GGP--------
B3--10   AHARRDYNERRGNENGRERGQGRFGGRPGGMQMGGPR-------------------------QD-------------------------GGP--------
B5--10   AHARRDFNERRGNENGRERGQGRFGGRPGGMQMGGSR-------------------------QD-------------------------GGP--------
B6--10   AHAQRDFNEQRGEENGRKRGQGRFRGRPGGMLMGGPR-------------------------QD-------------------------GGS--------
B7--10   AHAQRDYNELRGNKNGRERGQGRFGGRPGGMQMGGSRQDGGPMGGRRFDGPDSGAP--------------------------------------------
B8--10   AHARRDFNERRGNENGRERGQGRFGGRPGGMQMGGSR-------------------------QD-------------------------GGP--------
B8--G    AHARRDFNERRGNENGRERGQGRFGGRPGGMQMGGSR-------------------------QD-------------------------GGP--------
C2--4    AHAQRDYNELRGNKNGRERGQGRFGGRPGGMQMGGSRQDGGPMGGRRFDGPDSGAPQMDGRRQD-------------------------GGP--------
C3--4    AHAQRDYNERRGNENGRERGQGRFGGRPGGMQM-------------------------DGRRQD-------------------------GGP--------
C4--G    AHARRDFNERRGRENGRKRGQGGFGGRPDGMQ--------------------------------------------------------------------
C5--4    AHAQRDYNELRGNKNGRERGQGRFGGRPGGMQMGGSRQDGGPMGGRRFDGPDSGAPQMDGRRQ-------------------------------------
D1--2    AHAQRDYNELRGNKNGRERGQGRFGGRPGGMQMGGSRQDGGPMGGRRFDGPDSGAPQMDGRRQD-------------------------GGP--------
D1--4    AHAQRDYNELRGNKNGRERGQGRFGGRPGGMQMGGSRQDGGPMGGRRFDGPDSGAPQMDGRRQD-------------------------GGP--------
D1--10   AHAQRDYNELRGNENGRERGQGRFGGRPGGMQMGGPRQDGGPMGGRRFDGPDSGAPQMDGRRQD-------------------------GGP--------
D1--10   AHAQRDYNELRGNKNGRERGQGRFGGRPGGMQMGGSRQDGGPMGGRRFDGPDSGAPQMDGRRQD-------------------------GGP--------
D1d-G    AHAQRDYNERRGNENGRERGQGRFGGRPGGMQMGGSRQDGGPMGGKRFDGPDSGAPQMDGRRQD-------------------------GGP--------
D1f-G    AHAQRDYNERRGNENGRERGQGRFGGRPGGMQTGGPRQDGGPMGGRRFDGPDSGAPQMDGRRQD-------------------------GGP--------
D1y-G    AHAQRDYNELRGNKNGRERGQGRFGGRPGGMQMGGSRQDGGPMGGRRFDGPDSGAPQMDGRRQD-------------------------GGP--------
D5--10   AHAQRDFNEQRGEENGRKRGQGRFRGRPGGMQMGGPR-------------------------QD-------------------------GGS--------
D6--4    AHAQRDYNELRGNKNGRERGQGRFGGRPGGMQMGGSRQDGGPMGGRRFDGPDSGAPQMDGRRQD-------------------------GGP--------
D6--10   AHARRDYNERRGNENGRERGQGRFGGRPGGMQMGGPRQDGGPMGGRRFDGPDSGSPQMDGRRQD-------------------------GGP--------
D7--2    AHAQRDYNELRGNKNGRERGQGRFGGRPGGMQMGGSRQDGGPMGGRRFDGPDSGAPQMDGRRQD-------------------------GGP--------
E2--2    AHAQRDFNERRGKENDTERGQGGFGGRPGGMQMGGPR-------------------------QD-------------------------GGP--------
E2--4    AHAERDFNERRGKENGRERGQGGFGGRPGGMQTGSPR-------------------------QD-------------------------GGP--------
E2--10   AHAQRDFNERRGKENDTERGQGGFGGRPGGMQMGGPR-------------------------QD-------------------------GGP--------
E2b-G    AHARRDFNERRGKENGTERGQGGFGGRPGGMQTGSPR-------------------------QD------------------------------------
E2--G    AHAQRDFNERRGKENDTERGQGGFGGRPGGMQMGSPR-------------------------QD-------------------------GGQ--------
E3--2    AHAQRDYNELRGNKNGRERGQGRFGGRPGGMQMGGSRQDGGPMGGRRFDGPDSGAPQMDGRRQD-------------------------GGP--------
E3--2    AHARRDFNERRGKENGRERGQGGFGGRPGGMQTGSPR-------------------------QD-------------------------DGP--------
E3--4    AHAQRDFNERRGKENDTERGQGGFGGRPGGMQMGGPR-------------------------QD-------------------------GGP--------
E6--2    AHAQRDYNELRGNKNGRERGQGRFGGRPGGMQMGGSRQDGGPMGGRRFDGPDSGAPQMDGRRQD-------------------------GGP--------
E7--2    AHAERDFNERRGKENGRERGQGGFGGRPGGMQTGSPR-------------------------QD-------------------------GGP--------
E8--2    AHAQRDFNERRGKENDTERGQGGFGGRPGGMQMGGPR-------------------------QD-------------------------GGP--------
E9--2    AHAQRDFNERRGKENDTERGQGGFGGRPGGMQMGGPR-------------------------QD-------------------------GGP--------
E10-4    AHARRDFNELRGKENGRERGQGRFGGRPDGMQMGGPR-------------------------QDGGPMGGRRFDGPGFGAPQMGGPRQNGGP--------
F1--2    AHARRDFNERRGNENGRERGQGRLGAKPGGMQMGGSR-------------------------QD-------------------------GGP--------
01--2    AHARRDFNERRGKENGRERGQGGFGGRPGGMQTGSPR-------------------------QD-------------------------GGP--------
01--4    AHARRDFNERRGKENGRERGQGGFGGRPGGMQTGSPR-------------------------QD-------------------------GGP--------
01--10   AHAQRDYNERRGNENGRERGQGRFGGRPGGMQ-------------------------MDGRRQD-------------------------GGP--------
01--10   AHARRDFNERRGKENGRERGQGGFGGRPGGMQTGSPR-------------------------QD-------------------------GGP--------
01--10   AHARRDFNERRGKENGRERGQGGFGGRPGGMQTGSPR-------------------------QD-------------------------GGP--------
01--G    AHARRDFNERRGKENGRERGQGGFGGRPGGMQTGSPR-------------------------QD-------------------------GGP--------
08--2    AHARRDFNERRGKENGRERGQGGFGGRPGGMQTGSPR-------------------------QD-------------------------GGP--------
```

```
                 110       120       130       140       150       160       170       180       190       200
        ....|....|....|....|....|....|....|....|....|....|....|....|....|....|....|....|....|....|....|....|
A1--2   -----------------MGGRRFDGPGFGAPPMGGPRQDGGPMGGRRFDGPGFGAPQMGGPRQNGGPMGGRRFDGPGFGGSRPDGAGGRPFFGEGGRRGD
A2--G   -----------------MGGRRFDGPGFGAPPMGGPRQDGGPMGGRRFDGPGFGAPQMGGPRQNGGPMGGRRFDGPRFGGSRPDGAGGRPFFGEGGRRGD
G2--10  -----------------MGGRRFDGPRFGAPPMGGPRQDGGPMGGRRFDGPGFGAPQMGGPRQNGGPMGGRRFDGPGFGGSRPDGAGGRPFFGEGSRRGD
G3--10  -----------------MGGRRFDGPRFGAPPMGGPRQDGGPMGGRRFDGPGFGAPQMGGPRQNGGPMGGRRFDGPGFGGSRPDGAGGRPFFGEGSRRGD
B2--10  PDSGAPQMDGRRQDGGPMG-------------------------GRRFDGPGFGAPEMDGRRQNGGPMGGRRFDGPGFGGSRPDGAGGRPFFGQGGRRGD
B3--2   -----------------MG-------------------------GRRFDGPGFGAPQMDGRRQNGGPMGGRRFDGPRFGGSRPDGAGGRPFFGQGGRRGD
B3--4   -----------------MG-------------------------GRRFDGPGFGAPHMDGRRQNGGPMGGRRFDGPGFGGSRPDGAGGRPFFGQGGRRGD
B3--10  -----------------MG-------------------------GRRFDGPGFGAPQMDGRRQNGGPMGGRRFDGPRFGGSRPDGAGGRPFFGQGGRRGD
B5--10  -----------------MG-------------------------GRRFDGPGFGAPHMDGRRQNGGPMGGRRFDGPGFGGSRPDGAGGRPFFGQGGRRGD
B6--10  -----------------MG-------------------------GRRFDGPGLGAPQMDGRRQNGGPMGGRRFDGPGFGGSRPDGAGGRPFFGQGGRRGD
B7--10  --------------------------------------------------------QMDGRRQNGGPMGGRRFDGPGFGGSRPDGAGGRPFFGQGGRRGD
B8--10  -----------------MG-------------------------GRRFDGPGFGAPHMDGRRQNGGPMGGRRFDGPGFGGSRPDGAGGRPFFGQGGRRGD
B8--G   -----------------MG-------------------------GRRFDGPGFGAPHMDGRRQNGGPMGGRRFDGPGFGGSRPDGAGGRPFFGQGGRRGD
C2--4   -----------------MG-------------------------GRRFDGPGFGAPEMDGRRQNGGPMGGRRFDGPGFGGSRPDGAGGRPFFGQGGRRGD
C3--4   -----------------MG-------------------------GRRFDGPGFGAPQMDGRRQNGGPMGGRRFDGPGFGGSRPDGAGGRPFFGQGGRRGD
C4--G   -------------------------------------------------------------------MGGRRFDGPGFGGSRPDGAG-RPFFGQGGKRGD
C5--4   ---------------------------------------------------------------NGGPMGGRRFDGPGFGGSRPDGAGGRPFFGQGGRRGD
D1--2   -----------------MG-------------------------GRRFDGPGFGAPEMDGRRQNGGPMGGRRFDGPGFGGSRPDGAGGRPFFGQGGRRGD
D1--4   -----------------MG-------------------------GRRFDGPGFGAPEMDGRRQNGGPMGGRRFDGPGFGGSRPDGAGGRPFFGQGGRRGD
D1--10  -----------------MG-------------------------GRGFDGPGIGAPEMDGTEKNGGPMGGRELDGPGFGGSRPDGGGGRPFFGQGGRRGD
D1--10  -----------------MG-------------------------GRRFDGPGFGAPEMDGRRQNGGPMGGRRFDGPGFGGSRPVGAGGRLFFGQGGRRGD
D1d-G   -----------------MG-------------------------GRRFDGPGFGAPEMDGRRQNGGPMGGRRFDGPGFGGSRPVGAGGRPFFGQGGRRGD
D1f-G   -----------------MG-------------------------GRRFDGPGFGAPEMDGRRQNGGPMGGRRFDGPGFGGSRPVGAGGRPVFGQGGRRGD
D1y-G   -----------------MG-------------------------GRRFDGPGFGAPEMDGRRQNGGPMGGRRFDGPGFGGSRPDGAGGRPFFGQGGRRGD
D5--10  -----------------MG-------------------------GRRFDGPGLGAPQMDGRRQNGGPMGGRRFDGPGFGGSRPDGAGGRPFFGQGGRRGD
D6--4   -----------------MG-------------------------GRRFDGPGFGAPEMDGRRQNGGPMGGRRFDGPGFGGSRPDGAGGRPFFGQGGRRGD
D6--10  -----------------MG-------------------------GRRFDGPGFGAPEMDGRRQNGGPMGGRRFDGPGFGGSRPDGAGGRPFFGQGGRRGD
D7--2   -----------------MG-------------------------GRRFDGPGFGAPEMDGRRQNGGPMGGRRFDGPGFGGSRPDGAGGRPFFGQGGRRGD
E2--2   -----------------MG-------------------------GRRFDGPESGAPQMEGRRQNGGPMGGRRFDGPRFGGSRPDGAGGRPFFGQGGRRGD
E2--4   -----------------MG-------------------------GMRFDGPESGAPQMDGRRQNGGPMGGRRFDGPRFGGSRPDGAGGRPFFGQGGRRGD
E2--10  -----------------MG-------------------------GRRFDGPESGAPQMEGRRQNGGPMGGRRFDGPRFGGSRPDGAGGRPFFGQGGRRGD
E2b-G   ------------------G-------------------------GRRFDGPESGAPQMDGRRQNGGPMGGRRFDGPRFGGSRPDGAGGRPFFGQGGRRGD
E2--G   -----------------MG-------------------------GRRFDGPESGAPQMEGRRQNGGPMGGRRFDGPRFGGSRPDGAGGRPFFGQGGRRGD
E3--2   -----------------MG-------------------------GRRFD-------------------------GPGFGGSRPDGAGGRPFFGQGGRRGD
E3--2   -----------------MG-------------------------GRRFDGPESGAPQMDGRRQNGGPMGGRRFDGPRFGGSRPDGAGGRPFFGQGGRRGD
E3--4   -----------------MG-------------------------GRRFDGPESGAPQMEGRRQNGGPMGGRRFDGPGFGGSRPDGAGGRPFFGPGGRRGD
E6--2   -----------------MG-------------------------GRRFDGPGFGAPEMDGRRQNGGPMGGRRFDGPGFGGSRPVGAGGRPFFGQGGRRGD
E7--2   -----------------MG-------------------------GMRFDGPESGAPQMDGRRQNGGPMGGRRFDGPRFGGSRPDGAGGRPFFGQGGRRGD
E8--2   -----------------MG-------------------------GRRFDGPESGAPQMDGRRQNGGPMGGRRFDGPRFGGSRPDGAGGRPFFGQGGRRGD
E9--2   -----------------MG-------------------------GRRFDGPESGAPQMDGRRQNGGPMGGRRFDGPRFGGSRPDGAGGRPFFGQGGRRGD
E10-4   -----------------MG-------------------------GRRFDGPGFGAPQMGGPRQNGGPMGGRRFDGPGFGGSRPDCAGGRPFFGEGGRRGD
F1--2   -----------------VV-------------------------GRRFDGPGFGAPHMDGRRQNGGPMGGRRFDGPGFGGSRPDGAGGRPFFGQGGRRGD
01--2   -----------------MG-------------------------GMRFDGPESGAPQMDGRRQNGGPMGGRRFDGPRFGGSRPDGAGGRPFFGQGGRRGD
01--4   -----------------MG-------------------------GMRFDGPESGAPQMDGRRQNGGPMGGRRFDGPGFGGSRPDGAGGRPFFGQGGRRGD
01--10  -----------------MG-------------------------GRRFDGPESGAPQMDGRRQNGGPMGGRRFDGPGFGGSRPDGAGGRPFFGQGGRRGD
01--10  -----------------MG-------------------------GRRFDGPGFGAPQMDGRRQNGGPMGGRRFDGPRFGGSRPDGAGGRPFFGQGGRRDD
01--10  -----------------MG-------------------------GRRFDGPESGAPQMDGRRQNGGPMGGRRFDGPGFGGSRPDGAGGRPFFGQGGRRGD
01--G   -----------------MG-------------------------GMRFDGPESGAPQMDGRRQNGGPMGGRRFDGPGFGGSRPDGAGGRPFFGQGGRRGD
08--2   -----------------MG-------------------------GMRFDGPESGAPQMDGRRQNGGPMGGRRFDGPRFGGSRPDGAGGRPFFGQGGRRGD
```

```
                 210       220       230       240       250       260       270       280       290       300
       ....|....|....|....|....|....|....|....|....|....|....|....|....|....|....|....|....|....|....|....|
A1--2  GEEETDAAQQIGDGLGGPGRCDGPGHGHYGHHQGAGRPFFGNPPPFNPEQ-----EP---RNDNSEEDGRHHRHHDRHHAHHGHHG------H---HEHH
A2--G  GEEETDAARQI-----GPGRFDGPGHGHYGHHQGAGRPFFGNPPPFNPEQ-----EP---RNDSSEEDGRHHRHHDRHHAHHGHHG------H---HEHH
G2--10 GEEETDAARQIGDGLGGPGRFDGPGHGHYGHHQGAGRPFFGNPPPFNPEQ-----EP---RNDSSEEDGRHHRHHDRHHAHHGHHG------H---HEHH
G3--10 GEEETDAARQIGDGLGGPGRFDGPGHGHYGHHQGAGRPFFGNPPPFNPEQ-----EP---RNDSSEEDGRHHRHHDRHHAHHGHHG------H---HEHH
B2--10 GEEETDAAQQIGDGLGGPGQFDGHGRRHHGHRQG--------PP-----QDRPEEQPFGQRNYSNEEDGRPHPH------HHGHHG---HHGH---H--H
B3--2  GEEETDAAQQIGDGLGGRGQFDGHGRRHHGHRQG--------PP-----QDRPEEQPFGQRNYSNEEDGRPHPH------HHGHHG---HHGH---H--H
B3--4  GEEETDAAQQIGDGLGGRGRFDGHGRRHHGHRQG--------PP-----QDRPEEQPFGQRNERNEEDGRPHPH------HHGHHG---HHGH---H--H
B3--10 GEEETDAAQQIGDGLGGRGQFDGHGRRHHGHRQG--------PP-----QDRPEEQPFGQRNYSNEEDGRPHPH------HHGHHG---HHGH---H--H
B5--10 GEEETDAAQQIGDGLGGRGQFDGHGRRHHGHRQG--------PP-----QDRPEEQPFGQRNERNEEDSRPHPH------HHRHHG---HHDH---H--H
B6--10 GEEETDAAQQIGDGLGGPGQFDGHGRRRHGHRQG--------PP-----QDRSEEQPFGQRNESSEEDGRPHPH------HHRHHD------HHDRH--H
B7--10 GEEETDAAQQIGDGLGGSDRFDGLRRGHHGHRQG--------PP-----QDRPEEQPFGQRNESSEEDGRPHPH------HHRHHDHHD---R---H--H
B8--10 GEEETDAAQQIGDGLGGRGQFDGHGRRHHGHRQG--------PP-----QDRPEEQPFGQRNERNEEDSRPHPH------HHRHHG---HHDH---H--H
B8--G  GEEETDASQQIGDGLGGRGQFDGHGRRHHGHRQG--------PP-----QDRPEEQPFGQRNERNEEDGRPHPH------HHGHHG---HQGH---H--H
C2--4  GEEETDAAQQIGDGLGGSDRFDGPRRGHHGHRQG--------PP-----QDRPEEQPFGQRNYSSEEDGRPHPH------HHRHHGHH----R---H--H
C3--4  GEEETDAAQQIGDGLGGSDRFDGPRRGHHGHRQG--------PP-----QDRPEEQPFGQRNYSSEEDGRPHPH------HHRHHGHH----R---H--H
C4--G  GEEETDAAQQIGDGLGGSDRFDGPRRGHHGHRQG--------PP-----QDRPEEQPFGQRNESSEEDGRPHPHH---HGHHRHHR------H---H--H
C5--4  GEEETDAAQQIGDGLGGSDRFDGPRRGHHGHRQG--------PP-----QDRPEEQPFGQRNYSSEEDGRPHPH------HHRHHGHH----R---H--H
D1--2  GEEETDAAQQIGDGLGGPGQFDGPGRRHHGHRQG--------HP-----QDQAEEQPFGQRNESSEEDGRPHPH------HHRHHG------H---H--H
D1--4  GEEETDAAQQIGDGLGGPGQFDGPGRRHHGHRQG--------HP-----QDQAEEQPFGQRNESSEEDGRPHPH------HHRHHG------H---H--H
D1--10 EEEETDAAQQIGDGLGGPGQFDGPGRRHHGHRQG--------HP-----QDQAEEQPFGQRNESSEEDGRPHPH------HHRHHG------H---H--H
D1--10 GEEETDAAQQIGDGLGGPGQFDGPGRRHHGHRRG--------HP-----QDQAEEQPFGQRNESSEEDGRPHPH------HHRHHG------H---H--H
D1d-G  GEEETDAAQQIGDGLGGPGQFDGPGRRHHGHRQG--------HP-----QDQAEEQPFGQRNKSSEEDGRPHPH------HHRDHG------H---H--H
D1f-G  GEEETDAAQQIGDGLGGPGQFDGPGRRHHGHRQG--------HP-----QDQAEEQPFGQRNESSEEDGRPHPH------HHRHHG------H---H--H
D1y-G  GEEETDAAQQIGDGLGGPGQFDGPGRRHHGHRQG--------HP-----QDQAEEQPFGQRNESSEEDGRPHPH------HHRHHG------H---H--H
D5--10 GEEETDAAQQIGDGLGGPGQFDGPGRRHHGHRRG--------HP-----QDQAEEQPFGQRNESSEEDGRPHPH------HHRHHG------H---H--H
D6--4  GEEETDAAQQIGDGLGGPGQFDGPGRRHHGHRQG--------HP-----QDQAEEQPFGQRNESSEEDGRPHPH------HHRHHG------H---H--H
D6--10 GEEETDAAQQIGDGLGGPGQFDGPGRRHHGHRQG--------HP-----QDQAEEQPFGQRNESSEEDGRPHPH------HHRHHG------H---H--H
D7--2  GEEETDAAQQIGDGLGGPGQFDGPGRRHHGHRQG--------HP-----QDQAEEQPFGQRNESSEEDGRPHPH------HHRHHG------H---H--H
E2--10 GEEETDDDQKMGDGPGGRGQFDGHGRGHHGHRQG--------PP-----QDRPEEQPFGQRNESSDEDGRPHP---------RHHG------R---H--H
E2--2  GEEETDAAQQIGDGLGGRDQFDGHGRGHHGHRQG--------PP-----QDRPEEQPFGQRNESSDEDGRPHP---------RHHG------R---H--H
E2--4  GKEETDAAPLIGDGLGGRGQFDGHGRGHHGHRQG--------PP-----QDRPEEQPFGQRNESSDEDGRPHP---------RHHG------R---H--H
E2b-G  GEEETDAAQQIGDGLGGSGQFDGPRRGHHGHRQG--------PP-----QDRPEEQPFGQRNESSDEDGRPHP---------RHHG------R---H--H
E2--G  GEEETDAAQQIGDGLGGRGQFDGHGRGHHGHRQG--------PP-----QDRPEEQPFGQRNESSDEDGRPHP---------RHHG------R---H--H
E3--2  GEEETDAAQQIGDGLGGSGQFDGPRRGHHGHRQG--------PP-----QDRPEEQPFGQRNESSDEDGRPHP---------RHHG------R---H--H
E3--2  GEEETDAAQQMGDGLGGRGQFDGPRRGHHGHRQG--------PP-----QDRPEEQPFGQRNESSDEDGRPHP---------RHHG------R---H--H
E3--4  GEEETDAALLIGDVLGGRGQFDGPRRGHHGHRQG--------PP-----QDRPEEQPFGQRNESSDEDGRPHP---------RHHG------R---H--H
E6--2  GEEETDAAQQIGDGLGGRGQFDGHGRGHHGHRQG--------PP-----QDRPEEQPFGQRNESSDEDGRPHP---------RHHG------R---H--H
E7--2  GKEETDAAQQIGDGLGGRGQFDGHGRGHHGHRQG--------PP-----QDRPEEQPFGQRNESSDEDGRPHP---------RHHG------R---H--H
E8--2  GEEETDAAQQIGDGPGGRGQFDGHGRGHHGHRQG--------PP-----QDRPEEQPFGQRNESSDEDGRPHP---------RHHG------R---H--H
E9--2  GEEETDAAQQIGDGPGGRGQFDGHGRGHHGHRQG--------PP-----QDRPEEQPFGQRNESSDEDGRPHP---------RHHG------R---H--H
E10-4  GEEETDAARQIGDGLGGPGQFDGPRRGHHGHRQG--------PP-----QDRPEEQPFGQRNESSDEDGRPHPRH-------------------------
F1--2  GEEETDAAQQIGDGLGGRGQFDGHGRRHHGHRQG--------PP-----QDRPEEQPFGQRNERNEEDGRPHPH------HH---------GH---H--H
01--2  GEEETDAAQQIGDGLGGPGQFDGPGRRHHG----------------------------------------------------------------------
01--4  GEEETDAAQQIGDGLGGPGQFDGPGRRHHG----------------------------------------------------------------------
01--10 GEEETDAAQQIGDGLGGPGQFDGPGRRHHG----------------------------------------------------------------------
01--10 GEEETDAAQQIGDGLGGPGQFDGPGRRHHG----------------------------------------------------------------------
01--10 GEEETDAAQQIGDGLGGPGQFDGPGRRHH-----------------------------------------------------------------------
01--G  GEEETDAAQQIGDGLGGPGQFDGPGRRHHG----------------------------------------------------------------------
08--2  GEEETDAAQQIGDGLGGPGQFDGPGRRHHG----------------------------------------------------------------------
```

```
                   310       320       330       340       350       360       370       380       390       400
        ....|....|....|....|....|....|....|....|....|....|....|....|....|....|....|....|....|....|....|....|
A1--2   HQHH--N--------------------------------------------HTEGHQDHDRPMFEMRPFRFNPLGRKPFGDHPFGRRNHTEGHQGHNETG
A2--G   HQHH--N--------------------------------------------HTEGHQDHDRPMFEMRPFRFNPLGRKPFGDHPFGRRNHTEGHQGHNETG
G2--10  N-HH--N--------------------------------------------HTEGHQDHDRPMFEMRPFRFNPLGRKPFGDHPFGRRNHTEGHQGHNETG
G3--10  N-HH--N--------------------------------------------HTEGHQDHDRPMFEMRPFRFNP---------------------------
B2--10  R-HH--N--------------------------------------------QTE-------------------------------------GHQGHNETG
B3--2   R-HH--N--------------------------------------------QTE-------------------------------------GHQGHNETG
B3--4   R-HH--N--------------------------------------------QTE-------------------------------------GHQGHNETG
B3--10  R-HH--N--------------------------------------------QTE-------------------------------------GHQGHNETG
B5--10  R-HH--N--------------------------------------------QTE-------------------------------------GHQGHNETG
B6--10  R-HH--N--------------------------------------------HTE-------------------------------------GHQGHNETG
B7--10  R-HH--N--------------------------------------------HTE-------------------------------------GHQGHNETG
B8--10  R-HH--N--------------------------------------------QTE-------------------------------------GHQGHNETG
B8--G   R-HH--N--------------------------------------------QTE-------------------------------------GHQGHNETG
C2--4   H-HH--NQTEGHQGHSETGDQDQDKPIDTRPFRFNHFGRKPFGDRPFGRRNHTE-------------------------------------GHQGHNETG
C3--4   H-HH--NQTEGHQGHNETGDQDQDKPIDTRPFRFNHFGRKPFGDRPFGRRNHTE-------------------------------------GHQGHNETG
C4--G   --HH--N--------------------------------------------QTE-------------------------------------GHQGHNDTG
C5--4   H-HH--NQTEGHQGHNETGDQDQDKPIDTRPF--------------------------------------------------------------------
D1--2   R-HH--N--------------------------------------------HTE-------------------------------------GHQGHNETG
D1--4   R-HH--N--------------------------------------------HTE-------------------------------------GHQGHNETG
D1--10  R-HH--N--------------------------------------------HTE-------------------------------------GHQGHNETG
D1--10  R-HH--N--------------------------------------------HTE-------------------------------------GHQGHNETG
D1d-G   R-HH--N--------------------------------------------HTE-------------------------------------GHQGHNETG
D1f-G   R-HH--N--------------------------------------------HTE-------------------------------------GHQGHNETG
D1y-G   R-HH--N--------------------------------------------HTE-------------------------------------GHQGHNETG
D5--10  R-HH--N--------------------------------------------HTE-------------------------------------GHQGHNETG
D6--4   R-HH--N--------------------------------------------HTE-------------------------------------GHQGHNETG
D6--10  P-HH--N--------------------------------------------HTE-------------------------------------GHQGHNETG
D7--2   R-HH--N--------------------------------------------HTE-------------------------------------GHQGHNETG
E2--2   Q-HH-------------------------------------------------------------------------------------------H----
E2--4   Q-HH-------------------------------------------------------------------------------------------H----
E2--10  Q-HH-------------------------------------------------------------------------------------------H----
E2b-G   Q-HH-------------------------------------------------------------------------------------------H----
E2--G   Q-HH-------------------------------------------------------------------------------------------H----
E3--2   Q-HH-------------------------------------------------------------------------------------------H----
E3--2   Q-HHHRN--------------------------------------------HTE-------------------------------------GHQGHNETG
E3--4   Q-HH-------------------------------------------------------------------------------------------H----
E6--2   Q-HHHRN--------------------------------------------HTE-------------------------------------GHQGHNETG
E7--2   Q-HH-------------------------------------------------------------------------------------------H----
E8--2   Q-HH-------------------------------------------------------------------------------------------H----
E9--2   Q-HH-------------------------------------------------------------------------------------------H----
E10-4   ----------------------------------------------------------------------------------------------------
F1--2   R-HH--N--------------------------------------------QTE-------------------------------------GHQGHNETG
01--2   ----------------------------------------------------------------------------------------------------
01--4   ----------------------------------------------------------------------------------------------------
01--10  ----------------------------------------------------------------------------------------------------
01--10  ----------------------------------------------------------------------------------------------------
01--10  ----------------------------------------------------------------------------------------------------
01--G   ----------------------------------------------------------------------------------------------------
08--2   ----------------------------------------------------------------------------------------------------
```

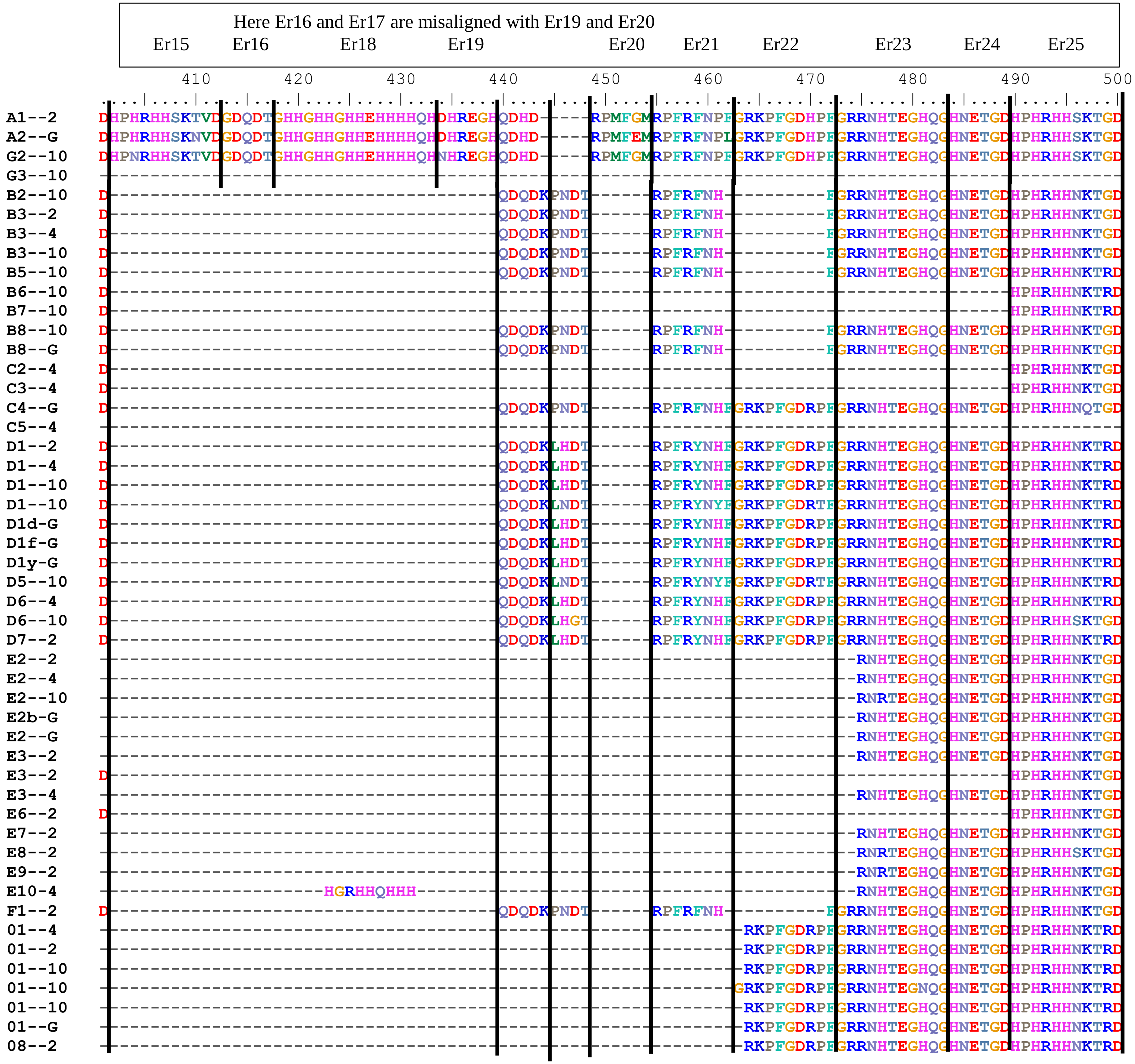
Here Er16 and Er17 are misaligned with Er19 and Er20
Er15 Er16 Er18 Er19 Er20 Er21 Er22 Er23 Er24 Er25
410 420 430 440 450 460 470 480 490 500

```
                    510       520       530       540       550       560       570       580       590       600
           ....|....|....|....|....|....|....|....|....|....|....|....|....|....|....|....|....|....|....|....|
A1--2      GDQDRPMFETRPFWVNPFGRKPFGDRPFDRRNGTEEGSPRRDGHPHPHGNRGRWGENESEEKEHPTTESVTTSSPLKVIEI--AINE-----VDTNVVAEV*
A2--G      GDQDRPMFETRPFWVNPFGRKPFGDRPFDRRNGTEEGSPRRDGHPHPHGNRGRWGENESEEKEHPTTESVTTSSPLKVIEI--AINE-----VDTNVVAEV*
G2--10     GDQDRPMFETRPFWVNPFGRKPFGDRPFDRRNGTEEGSPRRDGHPHPHGNRRRWGENESEEKEHPTTESVTTSSPLKVIEI--AINE-----VDTNVVAEV*
G3--10     -----------------FGRKPFGDRPFGRRNGTEEGSPRRDGQRRPYGNRGRWGENESEEKEHPTTESVTTSSPPEVVEI--AVNE-----EDVNVVAEV*
B2--10     GDQDRPMFEMRPFWVNPFGRKPFGDRPFGRRNGTEEGSPRRDGHRRPYGNRGRWGENESEEKEHPTTESVTTSSPPEVV----AINE-----EDINVVAEV*
B3--2      GDQDRPMFETRPFWVNPFGRKPFGDRPFGRRNGTEEGSPRRDGHRRPHGNRGRWGENESEEKEHPTTESVTTSSPPEVVEI--AIND----------VAEV*
B3--4      GDQDRPMFETRPFWVNPFGRKPFGDRPFGRRNGTEEGSPRRDGHRRPYGNRGRWGENESEEKEHPTTESVTTSSPPEVVEI--AVNE-----EDVNVVAEV*
B3--10     GDQDRPMFETRPFWVNPFGRKPFGDRPFGRRNGTEEGSPRRDGHRRPYGNRGRWGENESEEKEHPTTESVTTSSPPEVVEI--AVNE-----EDVNVVAEV*
B5--10     GDQDRPMFEMRPFRFNPFGRKPFGGRPFDRRNGTEEGSPRRDGHRRPYGNRGRWGENESEEKEHPTTESVTTSSPPEVV----AINE-----EDINVVAEV*
B6--10     GDQDRPMFEMRPFRFNPFGRKPFGGRPFDRRNGTEEGSPRRDGHRRPYGNRGRWGENESEEKERPTTESVTTSSPPEVVEI--AFNE-----EDVHVVAEV*
B7--10     GDQDRPMFEMRPFRFNPFGRKPFGGRPFDRRNGTEEGSPRRDGHRRPYGNRGRWGENESEEKERPTTESVTTSSPPEVVEI--AFNE-----EDVNVVAEV*
B8--10     GDQDRPMFEMRPFWVNPFGRKPFGDRPFGRRNGTEEGSPRRDGHRHPYGNRGRWGENESEEKEHPTTESVTTSSPPEVVEI--AVNE-----EDVNVVAEVL
B8--G      GDQDRPMFEMRPFWVNPFGRKPFGDRPFGRRNRTEEGSPRRDGHRHPYGNRGRWGENESEEKEHPTTESVTTSSPPEVVEI--AVNE-----EDVNVVAEVY
C2--4      GDQDRPMFESRPFRFNPFGRKPFGDRPFGRRNGTEEGSPRRDGHRRPYGNRGRWGENESEEKEHPTTESVTTSSPPEVVEI--AVNE-----EDVNVVAEV*
C3--4      GDQDRPMFESRPFRFNPFGRKPFGDRPFGRRNGTEEGSPRRDGHRRPYGNRGRWGENESEEKEHPTTESVTTSSPPEVVEI--AFNE-----EDVNVVAEV*
C4--G      GDQDRPMFEMRPFRFNPLGRKPFGDRPFGRRNGTEEGSLRRDGHRRPYGNRGRWGENESEEKEHPTTESVTTSSPPDVVEI--AVNE-----EDVNVVAEV*
C5--4      -------------RFNPFGRKPFGDRPFGRRNGTEEGSPRRDGHRRPYGNRGRWGENESEEKEHPTTESVTTSSPPEVVEI--AVNE-----EDVNVVAEV*
D1--2      GDQDRPMFEMRPFRFNPFGRKPFGGRPFDRRNGTEEGSPRRDGHRRPYGNRGRWGENESEEKEHPTTESVTTSSPPEV--V--AINE-----EDINVVAEV*
D1--4      GDQDRPMFEMRPFRFNPFGRKPFGGRPFDRRNGTEEGSPRRDGHRRPYGNRGRWGENESEEKEHPTTESVTTSSPPEV--V--AINE-----EDINVVAEV*
D1--10     GDQDRPMFEMRPFRFNPFGRKPFGGRPFDRRNGTEEGSPRRDGHRRPYGNRGRWGENESEEKEHPTTESVTTSSPPEV--V--AINE-----EDINVVAEV*
D1--10     GDQDRPMFEMRPFRFNPFGRKPFGGRPFDRRNGTEEGSPRRDGHRRPYGNRGRWGENESEEKEHPTTESVTTSSPPEV--V--AINE-----EDINVVAEV*
D1d-G      GDQDRPMFESRPFRFNPFGRKPFGDRLFGRRNGTEEGSSRRDGHRRPYGNRGRWGENESEEKEHPTTESVTTSSPPEV--V--AINE-----EDINVVAEV*
D1f-G      GDQDRPMFEMRPFRFNPLGRKPFGDRPFGRRNGTEEGSPRRDGQRRPYGNRGR*GENESEEKEHPTTESVTTSSPPEV--V--AINE-----EDINVVAEV*
D1y-G      GDQDRPMFEMRPFRFNPFRRKPFGGRPFDRRNGTEEGSPRRDGHRRPYGNRGRWGENESEEKEHPTTESVTTSSPPEV--V--AINE-----EDINVVAEV*
D5--10     GDQDRPMFEMRPFRFNPFGRKPFGGRPFDRRNGTEEGSPRRDGHRRPYGNRGRWGENESEEKEHPTTESVTTSSPPEVV----AINE-----EDINVVAEV*
D6--4      GDQDRPMFEMRPFWVNPFGRKPFGDRPFGRRNGTEEGSPRRDGHRRPYGNRGRWGENESEEKEHPTTESVTTSSPPEV--VEIAVNE-----EDVNVVAEV*
D6--10     GDQDRPMFETRPFWVNPFGRKPFGDRPFDRRNGTEEGSPRRDGHPHPHGNRGRWGENESEEKEHPTTESVTTSSPLKVIEI--AINE-----VDTNVVAEV*
D7--2      GDQDRPMFEMRPFRFNPFGRKPFGGRPFDRRNGTEEGSPRRDGHRRPYGNRGRWGENESEEKEHPTTESVTTSSPPEV--V--AINE-----EDINVVAEV*
E2--2      GDQDRPMFEMRPFRFNPFGRKPFGDRPFGRRNGTEEGSPRRDGQRRPYGNRGRWGENESEEKEHPTTESVTTSSPP*------------------------
E2--4      GDQDRPMFEMRPFRFNPFGRKPFGDRPFGRRNGTEEGSPRRDGQRRPYGNRGRWGENESEEKEYPTTESVTTSSPP*------------------------
E2--10     GDQDRPMFEMRPFRFNPFGRKPFGDRPFGRRNGTEEGSPRRDGQRRPHGNRGRWGENESEEKEHPTTESVTTYSPP*------------------------
E2b-G      GDQDRPMFEMRPFRFNPFGRKPFGDRPFGRRNGTEEGSPRRDGQRRPYGNRGRWGENESEEKEHPTTESVTTSSPP*------------------------
E2--G      GDQDRPMFEMRPFRFNPFGRKPFGDRPFGRRNGTEEGSPRRDGQRRPYGNRGRWGENESEEKEHPTTESVTTSSPP*------------------------
E3--2      GDQDRPMFEMRPFRFNPFGRKPFGDRPFGRRNGTEEGSPRRDGQRRPYGNRGRWGENESEEKEHPTTESVTTSSPP*------------------------
E3--2      GDQDRPMFEMRPFRFNPFGRKPFGDRPFGRRNGTEEGSPRRDGQRRPYGNRGRWGENESEEKEHPTTESVTTSSPP*------------------------
E3--4      GDQDRPMFEMRPFRFNPFGRKPFGDRPFGRRNGTEEGSPRRDGQRRPYGNRGRWGENESEEKEHPTTESVTTSSPP*------------------------
E6--2      GDQDRPMFEMRPFRFNPFGRKPFGDRPFGRRNGTEEGSPRRDGQRRPYGNRGRWGENESEEKEYPTTESVTTSSPP*------------------------
E7--2      GDQDRPMFEMRPFRFNPFGRKPFGDRPFGRRNGTEEGSPRRDGQRRPYGNRGRWGENESEEKEHPTTESVTTSSPPEVV----AINE-----EDINVVAEV*
E8--2      GDQDRPMFETRPFWVNPFGRKPFGDRPFDRRNGTEEGSPRRDGHPHPHGNRGRWGENESEEKEHPTTESVTTSSPLKVIEI--AINE-----VDTNVVAEV*
E9--2      GDQDRPMFEMRPFRFNPFGRKPFGDRPFGRRNGTEEGSPRRDGQRRPHGNRGRWGENESEEKEHPTTESVTTYSPP*------------------------
E10-4      GDQDRPMFEMRPFRFNPFGRKPFGDRPFGRRNGTEEGSPRRDGQRRPYGNRGRWGENESEEKEHPTTESVTTSSPP*------------------------
F1--2      RDQDRPMFEMRPFWVNPFGRKPFGDRPFGRRNGTEEGSPRRDGHRHPYGNRGRWGENESEEKEHPTTESITTSSPPEVVEI--AVNE-----EDVNVVAEV*
01--4      GDQDRPMFEMRPFRFNPFGRKPFGDRPFGRRNGTEEGSPRRDGHRRPYGNRGRWGENESEEKEHPTTESVTTSSPPEVVEI--AIND----------VAEV*
01--2      GDQDRPMFEMRPFRFNPFGRKPFGDRPFGRRNGTEEGSPRRDGQRRPYGNRGRWGENESEEKEHPTMESVTTSSPPEVVEI--AINEVDTNG-----VAEV*
01--10     GDQDRPMFEMRPFRFNPFGRKPFGDRPFGRRNGTEEGSPRRDGQRRPHGNRGRWGENESEEKEHPTTESVTTSSPPEVVEI--AIND----------VAEV*
01--10     GDQDRPMFEMRPFRFNPFGRKPFGDRPFGRRNGTEEGSPRRDGQRRPHGNRGRWGENESEEKEHPTTESVTTSSPPEVVEI--AIND----------VAEV*
01--10     GDQDRPMFEMRPFRFNPFGRKPFGDRPFGRRNGTEEGSPRRDGQRRPHGNRGRWGENESEEKEHPTTESVTTSSPPEVVEI--AIND----------VAEV*
01--G      GDQDRPMFEMRPFRFNPFGRKPFGDRPFGRRNGTEEGSPRRDGQRRPHGNRGRWGENESEEKEHPTTESVTTSSPPDVVEI--AIND----------VAEV*
08--2      GDQDRPMFEMRPFRFNPFGRKPFGDRPFGRRNGTEEGSPRRDGQRRPYGNRGRWGENESEEKEYPTTESVTTSSPP*------------------------
```

Figure S2. The NJGT-PRANK alignment of representative deduced SpTrf protein sequences increases the number of gaps in the alignment. The NJGT-PRANK alignment was done using webPRANK with the deduced SpTrf protein sequences from animals as in Figure S1. The deduced amino acid sequences of the second exon of the *SpTrf* genes are shown. Standard parameters were used in webPRANK (gap rate, 0.05; gap length, 5; K, 2.0) and the sequences were set to align by translated codons and to trust insertions (+F). Substitution scoring was set to relaxed and the neighbor joining guide tree generation was done in webPRANK using ClustalW2. Explanations of protein names, dashes, and asterisks are the same as in Figure S1. Element boarders are based on the repeat-based alignment and are indicated by vertical black lines. Horizontal black lines indicate regions where non-matching elements overlap. Notes on the alignment and elements of interest are shown above those regions. Er is the abbreviation for elements in the repeat-based alignment. The sequences are arranged with the L proteins at the top, the 0 proteins at the bottom, and the remaining S proteins in alphabetical order

```
               10        20        30        40        50        60        70        80        90       100
         ....|....|....|....|....|....|....|....|....|....|....|....|....|....|....|....|....|....|....|....|
A1--2    AHAQSDFNERRGKENGRERGQDRFGGRPDGMQMGG-------------------------PRQDGGPMGGRRFDGPRFGAPQMGGPRQNGGPMGGRRFDG
A2--G    AHAQSDFNERRGKENGRERGQDRFGGRPDGMQMGG-------------------------PRQDGGPMGGRRFDGPRFGAPQMGGPRQNGGPMGGRRFDG
G2--10   AHARRDFNELRGKENGRERGQGRFGGRPDGMQMGG-------------------------PRQDGGPMGGRRFDGPGFGAPQMGGPRQNGGPMGGRRFDG
G3--10   AHARRDFNELRGKENGRERGQGRFGGRPDGMQMGG-------------------------PRQDGGPMGGRRFDGPGFGAPQMGGPRQNGGPMGGRRFDG
B2--10   AHAQRDYNELRGNKNGRERGQGRFGGRPGGMQMGGSRQDGGPMGGRRFDGPDSGAPQMDGRRQD-------------------------GGPM-------
B3--2    AHARRDYNERRGNENGRERGQGRFGGRPGGMQMGG-------------------------PRQD-------------------------GGPM-------
B3--4    AHARRDFNERRGNENGRERGQDRFGGRPGGMQMGG-------------------------SRQD-------------------------GGPM-------
B3--10   AHARRDYNERRGNENGRERGQGRFGGRPGGMQMGG-------------------------PRQD-------------------------GGPM-------
B5--10   AHARRDFNERRGNENGRERGQGRFGGRPGGMQMGG-------------------------SRQD-------------------------GGPM-------
B6--10   AHAQRDFNEQRGEENGRKRGQGRFRGRPGGMLMGG-------------------------PRQD-------------------------GGSM-------
B7--10   AHAQRDYNELRGNKNGRERGQGRFGGRPGGMQMGGSRQDGGPMGGRRFDGPDSGAP--------------------------------------------
B8--10   AHARRDFNERRGNENGRERGQGRFGGRPGGMQMGG-------------------------SRQD-------------------------GGPM-------
B8--G    AHARRDFNERRGNENGRERGQGRFGGRPGGMQMGG-------------------------SRQD-------------------------GGPM-------
C2--4    AHAQRDYNELRGNKNGRERGQGRFGGRPGGMQMGGSRQDGGPMGGRRFDGPDSGAPQMDGRRQD-------------------------GGPM-------
C3--4    AHAQRDYNERRGNENGRERGQGRFGGRPGGMQM-------------------------DGRRQD-------------------------GGPM-------
C4--G    AHARRDFNERRGRENGRKRGQGGFGGRPDGMQ--------------------------------------------------------------------
C5--4    AHAQRDYNELRGNKNGRERGQGRFGGRPGGMQMGGSRQDGGPMGGRRFDGPDSGAPQMDGRRQ-------------------------------------
D1--2    AHAQRDYNELRGNKNGRERGQGRFGGRPGGMQMGGSRQDGGPMGGRRFDGPDSGAPQMDGRRQD-------------------------GGPM-------
D1--4    AHAQRDYNELRGNKNGRERGQGRFGGRPGGMQMGGSRQDGGPMGGRRFDGPDSGAPQMDGRRQD-------------------------GGPM-------
D1--10   AHAQRDYNELRGNENGRERGQGRFGGRPGGMQMGGPRQDGGPMGGRRFDGPDSGAPQMDGRRQD-------------------------GGPM-------
D1--10   AHAQRDYNELRGNKNGRERGQGRFGGRPGGMQMGGSRQDGGPMGGRRFDGPDSGAPQMDGRRQD-------------------------GGPM-------
D1d-G    AHAQRDYNERRGNENGRERGQGRFGGRPGGMQMGGSRQDGGPMGGKRFDGPDSGAPQMDGRRQD-------------------------GGPM-------
D1f-G    AHAQRDYNERRGNENGRERGQGRFGGRPGGMQTGGPRQDGGPMGGRRFDGPDSGAPQMDGRRQD-------------------------GGPM-------
D1y-G    AHAQRDYNELRGNKNGRERGQGRFGGRPGGMQMGGSRQDGGPMGGRRFDGPDSGAPQMDGRRQD-------------------------GGPM-------
D5--10   AHAQRDFNEQRGEENGRKRGQGRFRGRPGGMQMGG-------------------------PRQD-------------------------GGSM-------
D6--4    AHAQRDYNELRGNKNGRERGQGRFGGRPGGMQMGGSRQDGGPMGGRRFDGPDSGAPQMDGRRQD-------------------------GGPM-------
D6--10   AHARRDYNERRGNENGRERGQGRFGGRPGGMQMGGPRQDGGPMGGRRFDGPDSGSPQMDGRRQD-------------------------GGPM-------
D7--2    AHAQRDYNELRGNKNGRERGQGRFGGRPGGMQMGGSRQDGGPMGGRRFDGPDSGAPQMDGRRQD-------------------------GGPM-------
E2--2    AHAQRDFNERRGKENDTERGQGGFGGRPGGMQMGG-------------------------PRQD-------------------------GGPM-------
E2--4    AHAERDFNERRGKENGRERGQGGFGGRPGGMQTGS-------------------------PRQD-------------------------GGPM-------
E2--10   AHAQRDFNERRGKENDTERGQGGFGGRPGGMQMGG-------------------------PRQD-------------------------GGPM-------
E2b-G    AHARRDFNERRGKENGTERGQGGFGGRPGGMQTGS-------------------------PRQD------------------------------------
E2--G    AHAQRDFNERRGKENDTERGQGGFGGRPGGMQMGS-------------------------PRQD-------------------------GGQM-------
E3--2    AHAQRDYNELRGNKNGRERGQGRFGGRPGGMQMGGSRQDGGPMGGRRFDGPDSGAPQMDGRRQD-------------------------GGPM-------
E3--2    AHARRDFNERRGKENGRERGQGGFGGRPGGMQTGS-------------------------PRQD-------------------------DGPM-------
E3--4    AHAQRDFNERRGKENDTERGQGGFGGRPGGMQMGG-------------------------PRQD-------------------------GGPM-------
E6--2    AHAQRDYNELRGNKNGRERGQGRFGGRPGGMQMGGSRQDGGPMGGRRFDGPDSGAPQMDGRRQD-------------------------GGPM-------
E7--2    AHAERDFNERRGKENGRERGQGGFGGRPGGMQTGS-------------------------PRQD-------------------------GGPM-------
E8--2    AHAQRDFNERRGKENDTERGQGGFGGRPGGMQMGG-------------------------PRQD-------------------------GGPM-------
E9--2    AHAQRDFNERRGKENDTERGQGGFGGRPGGMQMGG-------------------------PRQD-------------------------GGPM-------
E10-4    AHARRDFNELRGKENGRERGQGRFGGRPDGMQMGG-------------------------PRQDGGPMGGRRFDGPGFGAPQMGGPRQNGGPM-------
F1--2    AHARRDFNERRGNENGRERGQGRLGAKPGGMQMGG-------------------------SRQD-------------------------GGPV-------
01--2    AHARRDFNERRGKENGRERGQGGFGGRPGGMQTGS-------------------------PRQD-------------------------GGPM-------
01--4    AHARRDFNERRGKENGRERGQGGFGGRPGGMQTGS-------------------------PRQD-------------------------GGPM-------
01--10   AHARRDFNERRGKENGRERGQGGFGGRPGGMQTGS-------------------------PRQD-------------------------GGPM-------
01--10   AHARRDFNERRGKENGRERGQGGFGGRPGGMQTGS-------------------------PRQD-------------------------GGPM-------
01--10   AHAQRDYNERRGNENGRERGQGRFGGRPGGMQMDG-------------------------RRQD-------------------------GGPM-------
01--G    AHARRDFNERRGKENGRERGQGGFGGRPGGMQTGS-------------------------PRQD-------------------------GGPM-------
08--2    AHARRDFNERRGKENGRERGQGGFGGRPGGMQTGS-------------------------PRQD-------------------------GGPM-------
```

```
               110       120       130       140       150       160       170       180       190       200
        ....|....|....|....|....|....|....|....|....|....|....|....|....|....|....|....|....|....|....|....|
A1--2   PGFGAPPMGGPRQDGGPMGGRRFDGPGFGAPQMGGPRQNGGPMGGRRFDGPGFGGSRPDGAGGRPFFGEGGRRGDGEEETDAAQQIGDGLGGPGRCDGPG
A2--G   PGFGAPPMGGPRQDGGPMGGRRFDGPGFGAPQMGGPRQNGGPMGGRRFDGPRFGGSRPDGAGGRPFFGEGGRRGDGEEETDAARQI-----GPGRFDGPG
G2--10  PRFGAPPMGGPRQDGGPMGGRRFDGPGFGAPQMGGPRQNGGPMGGRRFDGPGFGGSRPDGAGGRPFFGEGSRRGDGEEETDAARQIGDGLGGPGRFDGPG
G3--10  PRFGAPPMGGPRQDGGPMGGRRFDGPGFGAPQMGGPRQNGGPMGGRRFDGPGFGGSRPDGAGGRPFFGEGSRRGDGEEETDAARQIGDGLGGPGRFDGPG
B2--10  ------------------GGRRFDGPGFGAPEMDGRRQNGGPMGGRRFDGPGFGGSRPDGAGGRPFFGQGGRRGDGEEETDAAQQIGDGLGGPGQFDGHG
B3--2   ------------------GGRRFDGPGFGAPQMDGRRQNGGPMGGRRFDGPRFGGSRPDGAGGRPFFGQGGRRGDGEEETDAAQQIGDGLGGRGQFDGHG
B3--4   ------------------GGRRFDGPGFGAPHMDGRRQNGGPMGGRRFDGPGFGGSRPDGAGGRPFFGQGGRRGDGEEETDAAQQIGDGLGGRGRFDGHG
B3--10  ------------------GGRRFDGPGFGAPQMDGRRQNGGPMGGRRFDGPRFGGSRPDGAGGRPFFGQGGRRGDGEEETDAAQQIGDGLGGRGQFDGHG
B5--10  ------------------GGRRFDGPGFGAPHMDGRRQNGGPMGGRRFDGPGFGGSRPDGAGGRPFFGQGGRRGDGEEETDAAQQIGDGLGGRGQFDGHG
B6--10  ------------------GGRRFDGPGLGAPQMDGRRQNGGPMGGRRFDGPGFGGSRPDGAGGRPFFGQGGRRGDGEEETDAAQQIGDGLGGPGQFDGHG
B7--10  -------------------------------QMDGRRQNGGPMGGRRFDGPGFGGSRPDGAGGRPFFGQGGRRGDGEEETDAAQQIGDGLGGSDRFDGLR
B8--10  ------------------GGRRFDGPGFGAPHMDGRRQNGGPMGGRRFDGPGFGGSRPDGAGGRPFFGQGGRRGDGEEETDAAQQIGDGLGGRGQFDGHG
B8--G   ------------------GGRRFDGPGFGAPHMDGRRQNGGPMGGRRFDGPGFGGSRPDGAGGRPFFGQGGRRGDGEEETDASQQIGDGLGGRGQFDGHG
C2--4   ------------------GGRRFDGPGFGAPEMDGRRQNGGPMGGRRFDGPGFGGSRPDGAGGRPFFGQGGRRGDGEEETDAAQQIGDGLGGSDRFDGPR
C3--4   ------------------GGRRFDGPGFGAPQMDGRRQNGGPMGGRRFDGPGFGGSRPDGAGGRPFFGQGGRRGDGEEETDAAQQIGDGLGGSDRFDGPR
C4--G   ------------------------------------------MGGRRFDGPGFGGSRPDGAG-RPFFGQGGKRGDGEEETDAAQQIGDGLGGSDRFDGPR
C5--4   --------------------------------------NGGPMGGRRFDGPGFGGSRPDGAGGRPFFGQGGRRGDGEEETDAAQQIGDGLGGSDRFDGPR
D1--2   ------------------GGRRFDGPGFGAPEMDGRRQNGGPMGGRRFDGPGFGGSRPDGAGGRPFFGQGGRRGDGEEETDAAQQIGDGLGGPGQFDGPG
D1--4   ------------------GGRRFDGPGFGAPEMDGRRQNGGPMGGRRFDGPGFGGSRPDGAGGRPFFGQGGRRGDGEEETDAAQQIGDGLGGPGQFDGPG
D1--10  ------------------GGRGFDGPGIGAPEMDGTEKNGGPMGGRELDGPGFGGSRPDGGGGRPFFGQGGRRGDGEEETDAAQQIGDGLGGPGQFDGPG
D1--10  ------------------GGRRFDGPGFGAPEMDGRRQNGGPMGGRRFDGPGFGGSRPVGAGGRLFFGQGGRRGDEEEETDAAQQIGDGLGGPGQFDGPG
D1d-G   ------------------GGRRFDGPGFGAPEMDGRRQNGGPMGGRRFDGPGFGGSRPVGAGGRPFFGQGGRRGDGEEETDAAQQIGDGLGGPGQFDGPG
D1f-G   ------------------GGRRFDGPGFGAPEMDGRRQNGGPMGGRRFDGPGFGGSRPVGAGGRPVFGQGGRRGDGEEETDAAQQIGDGLGGPGQFDGPG
D1y-G   ------------------GGRRFDGPGFGAPEMDGRRQNGGPMGGRRFDGPGFGGSRPDGAGGRPFFGQGGRRGDGEEETDAAQQIGDGLGGPGQFDGPG
D5--10  ------------------GGRRFDGPGLGAPQMDGRRQNGGPMGGRRFDGPGFGGSRPDGAGGRPFFGQGGRRGDGEEETDAAQQIGDGLGGPGQFDGPG
D6--4   ------------------GGRRFDGPGFGAPEMDGRRQNGGPMGGRRFDGPGFGGSRPDGAGGRPFFGQGGRRGDGEEETDAAQQIGDGLGGPGQFDGPG
D6--10  ------------------GGRRFDGPGFGAPEMDGRRQNGGPMGGRRFDGPGFGGSRPDGAGGRPFFGQGGRRGDGEEETDAAQQIGDGLGGPGQFDGPG
D7--2   ------------------GGRRFDGPGFGAPEMDGRRQNGGPMGGRRFDGPGFGGSRPDGAGGRPFFGQGGRRGDGEEETDAAQQIGDGLGGPGQFDGPG
E2--2   ------------------GGRRFDGPESGAPQMEGRRQNGGPMGGRRFDGPRFGGSRPDGAGGRPFFGQGGRRGDGEEETDAAQQIGDGLGGRDQFDGHG
E2--4   ------------------GGMRFDGPESGAPQMDGRRQNGGPMGGRRFDGPRFGGSRPDGAGGRPFFGQGGRRGDGKEETDAAPLIGDGLGGRGQFDGHG
E2--10  ------------------GGRRFDGPESGAPQMEGRRQNGGPMGGRRFDGPRFGGSRPDGAGGRPFFGQGGRRGDGEEETDDDQKMGDGPGGRGQFDGHG
E2b-G   ------------------GGRRFDGPESGAPQMDGRRQNGGPMGGRRFDGPRFGGSRPDGAGGRPFFGQGGRRGDGEEETDAAQQIGDGLGGSGQFDGPR
E2--G   ------------------GGRRFDGPESGAPQMEGRRQNGGPMGGRRFDGPRFGGSRPDGAGGRPFFGQGGRRGDGEEETDAAQQIGDGLGGRGQFDGHG
E3--2   ------------------GGRRFDGPESGAPQMDGRRQNGGPMGGRRFDGPRFGGSRPDGAGGRPFFGQGGRRGDGEEETDAAQQIGDGLGGSGQFDGPR
E3--2   ------------------GGRRFD-------------------------GPGFGGSRPDGAGGRPFFGQGGRRGDGEEETDAAQQMGDGLGGRGQFDGPR
E3--4   ------------------GGRRFDGPESGAPQMEGRRQNGGPMGGRRFDGPGFGGSRPDGAGGRPFFGPGGRRGDGEEETDAALLIGDVLGGRGQFDGPR
E6--2   ------------------GGRRFDGPGFGAPEMDGRRQNGGPMGGRRFDGPGFGGSRPVGAGGRPFFGQGGRRGDGEEETDAAQQIGDGLGGRGQFDGHG
E7--2   ------------------GGMRFDGPESGAPQMDGRRQNGGPMGGRRFDGPRFGGSRPDGAGGRPFFGQGGRRGDGKEETDAAQQIGDGLGGRGQFDGHG
E8--2   ------------------GGRRFDGPESGAPQMDGRRQNGGPMGGRRFDGPRFGGSRPDGAGGRPFFGQGGRRGDGEEETDAAQQIGDGPGGRGQFDGHG
E9--2   ------------------GGRRFDGPESGAPQMDGRRQNGGPMGGRRFDGPRFGGSRPDGAGGRPFFGQGGRRGDGEEETDAAQQIGDGPGGRGQFDGHG
E10-4   ------------------GGRRFDGPGFGAPQMGGPRQNGGPMGGRRFDGPGFGGSRPDCAGGRPFFGEGGRRGDGEEETDAARQIGDGLGGPGQFDGPR
F1--2   ------------------VGRRFDGPGFGAPHMDGRRQNGGPMGGRRFDGPGFGGSRPDGAGGRPFFGQGGRRGDGEEETDAAQQIGDGLGGRGQFDGHG
01--2   ------------------GGMRFDGPESGAPQMDGRRQNGGPMGGRRFDGPRFGGSRPDGAGGRPFFGQGGRRGDGEEETDAAQQIGDGLGGPGQFDGPG
01--4   ------------------GGMRFDGPESGAPQMDGRRQNGGPMGGRRFDGPGFGGSRPDGAGGRPFFGQGGRRGDGEEETDAAQQIGDGLGGPGQFDGPG
01--10  ------------------GGRRFDGPESGAPQMDGRRQNGGPMGGRRFDGPGFGGSRPDGAGGRPFFGQGGRRGDGEEETDAAQQIGDGLGGPGQFDGPG
01--10  ------------------GGRRFDGPGFGAPQMDGRRQNGGPMGGRRFDGPRFGGSRPDGAGGRPFFGQGGRRDDGEEETDAAQQIGDGLGGPGQFDGPG
01--10  ------------------GGRRFDGPESGAPQMDGRRQNGGPMGGRRFDGPGFGGSRPDGAGGRPFFGQGGRRGDGEEETDAAQQIGDGLGGPGQFDGPG
01--G   ------------------GGMRFDGPESGAPQMDGRRQNGGPMGGRRFDGPGFGGSRPDGAGGRPFFGQGGRRGDGEEETDAAQQIGDGLGGPGQFDGPG
08--2   ------------------GGMRFDGPESGAPQMDGRRQNGGPMGGRRFDGPRFGGSRPDGAGGRPFFGQGGRRGDGEEETDAAQQIGDGLGGPGQFDGPG
```

L-Er7 & S-Er8 aligned together

S-Er10 to SEr16 are aligned separately

```
               210       220       230       240       250       260       270       280       290       300
        ....|....|....|....|....|....|....|....|....|....|....|....|....|....|....|....|....|....|....|....|
A1--2   HGHYGHHQGAGRPFFGNPPPFNPEQEP-----------RNDNSEEDGRHHRHHDRHHAHHGHHGHHEHHHQHHNHTEGHQDHDRPMFEMRPFRFNPLGRK
A2--G   HGHYGHHQGAGRPFFGNPPPFNPEQEP-----------RNDSSEEDGRHHRHHDRHHAHHGHHGHHEHHHQHHNHTEGHQDHDRPMFEMRPFRFNPLGRK
G2--10  HGHYGHHQGAGRPFFGNPPPFNPEQEP-----------RNDSSEEDGRHHRHHDRHHAHHGHHGHHEHHN-HHNHTEGHQDHDRPMFEMRPFRFNPLGRK
G3--10  HGHYGHHQGAGRPFFGNPPPFNPEQEP-----------RNDSSEEDGRHHRHHDRHHAHHGHHGHHEHHN-HHNHTEGHQDHDRPMFEMRPFRFNP----
B2--10  RRHHGHRQG---------PP-------QDRPEEQPFGQRNYSNEEDGRPHPHH-----------------------------------------------
B3--10  RRHHGHRQG---------PP-------QDRPEEQPFGQRNYSNEEDGRPHPHH-----------------------------------------------
B3--2   RRHHGHRQG---------PP-------QDRPEEQPFGQRNYSNEEDGRPHPHH-----------------------------------------------
B3--4   RRHHGHRQG---------PP-------QDRPEEQPFGQRNERNEEDGRPHPHH-----------------------------------------------
B5--10  RRHHGHRQG---------PP-------QDRPEEQPFGQRNERNEEDSRPHPHH-----------------------------------------------
B6--10  RRRHGHRQG---------PP-------QDRSEEQPFGQRNESSEEDGRPHPHH-----------------------------------------------
B7--10  RGHHGHRQG---------PP-------QDRPEEQPFGQRNESSEEDGRPHPHH-----------------------------------------------
B8--10  RRHHGHRQG---------PP-------QDRPEEQPFGQRNERNEEDSRPHPHH-----------------------------------------------
B8--G   RRHHGHRQG---------PP-------QDRPEEQPFGQRNERNEEDGRPHPHH-----------------------------------------------
C2--4   RGHHGHRQG---------PP-------QDRPEEQPFGQRNYSSEEDGRPHPHH-----------------------------------------------
C3--4   RGHHGHRQG---------PP-------QDRPEEQPFGQRNYSSEEDGRPHPHH-----------------------------------------------
C4--G   RGHHGHRQG---------PP-------QDRPEEQPFGQRNESSEEDGRPHPHH-----------------------------------------------
C5--4   RGHHGHRQG---------PP-------QDRPEEQPFGQRNYSSEEDGRPHPHH-----------------------------------------------
D1--2   RRHHGHRQG---------HP-------QDQAEEQPFGQRNESSEEDGRPHPHH-----------------------------------------------
D1--4   RRHHGHRQG---------HP-------QDQAEEQPFGQRNESSEEDGRPHPHH-----------------------------------------------
D1--10  RRHHGHRQG---------HP-------QDQAEEQPFGQRNESSEEDGRPHPHH-----------------------------------------------
D1--10  RRHHGHRRG---------HP-------QDQAEEQPFGQRNESSEEDGRPHPHH-----------------------------------------------
D1d-G   RRHHGHRQG---------HP-------QDQAEEQPFGQRNKSSEEDGRPHPHH-----------------------------------------------
D1f-G   RRHHGHRQG---------HP-------QDQAEEQPFGQRNESSEEDGRPHPHH-----------------------------------------------
D1y-G   RRHHGHRQG---------HP-------QDQAEEQPFGQRNESSEEDGRPHPHH-----------------------------------------------
D5--10  RRHHGHRRG---------HP-------QDQAEEQPFGQRNESSEEDGRPHPHH-----------------------------------------------
D6--4   RRHHGHRQG---------HP-------QDQAEEQPFGQRNESSEEDGRPHPHH-----------------------------------------------
D6--10  RRHHGHRQG---------HP-------QDQAEEQPFGQRNESSEEDGRPHPHH-----------------------------------------------
D7--2   RRHHGHRQG---------HP-------QDQAEEQPFGQRNESSEEDGRPHPHH-----------------------------------------------
E2--2   RGHHGHRQG---------PP-------QDRPEEQPFGQRNESSDEDGRPHPRH-----------------------------------------------
E2--4   RGHHGHRQG---------PP-------QDRPEEQPFGQRNESSDEDGRPHPRH-----------------------------------------------
E2--10  RGHHGHRQG---------PP-------QDRPEEQPFGQRNESSDEDGRPHPRH-----------------------------------------------
E2b-G   RGHHGHRQG---------PP-------QDRPEEQPFGQRNESSDEDGRPHPRH-----------------------------------------------
E2--G   RGHHGHRQG---------PP-------QDRPEEQPFGQRNESSDEDGRPHPRH-----------------------------------------------
E3--2   RGHHGHRQG---------PP-------QDRPEEQPFGQRNESSDEDGRPHP-------------------------------------------------
E3--2   RGHHGHRQG---------PP-------QDRPEEQPFGQRNESSDEDGRPHPRH-----------------------------------------------
E3--4   RGHHGHRQG---------PP-------QDRPEEQPFGQRNESSDEDGRPHPRH-----------------------------------------------
E6--2   RGHHGHRQG---------PP-------QDRPEEQPFGQRNESSDEDGRPHP-------------------------------------------------
E7--2   RGHHGHRQG---------PP-------QDRPEEQPFGQRNESSDEDGRPHPRH-----------------------------------------------
E8--2   RGHHGHRQG---------PP-------QDRPEEQPFGQRNESSDEDGRPHPRH-----------------------------------------------
E9--2   RGHHGHRQG---------PP-------QDRPEEQPFGQRNESSDEDGRPHPRH-----------------------------------------------
E10-4   RGHHGHRQG---------PP-------QDRPEEQPFGQRNESSDEDGRPHPRH-----------------------------------------------
F1--2   RRHHGHRQG---------PP-------QDRPEEQPFGQRNERNEEDGRPHPHH-----------------------------------------------
01--2   RRHHG-----------------------------------------------------------------------------------------------
01--4   RRHHG-----------------------------------------------------------------------------------------------
01--10  RRHHG-----------------------------------------------------------------------------------------------
01--10  RRHHG-----------------------------------------------------------------------------------------------
01--10  RRHHG-----------------------------------------------------------------------------------------------
01--G   RRHHG-----------------------------------------------------------------------------------------------
08--2   RRHHG-----------------------------------------------------------------------------------------------
```

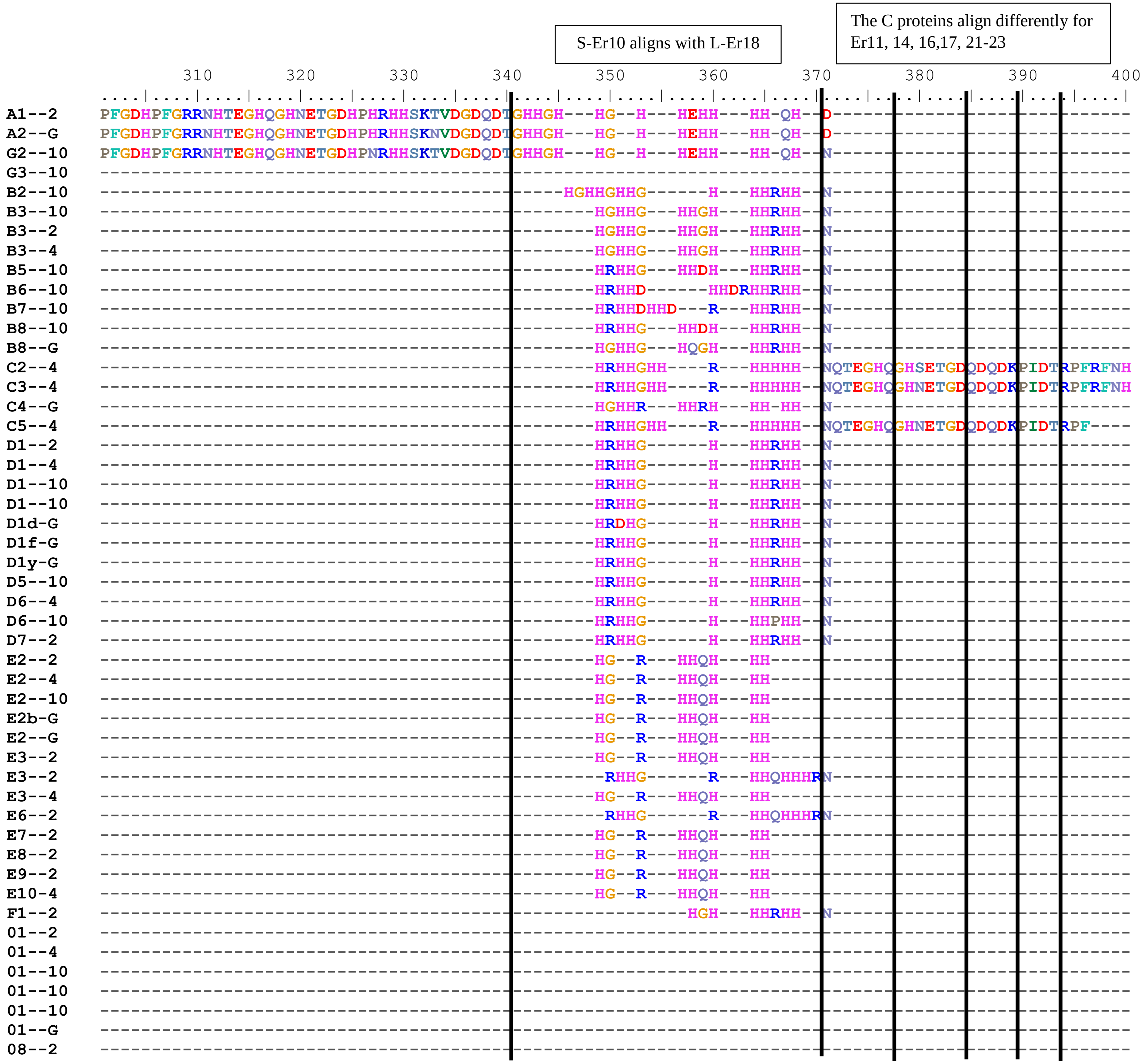
S-Er10 aligns with L-Er18
The C proteins align differently for Er11, 14, 16,17, 21-23
310
320
330
340
350
360
370
380
390
400
A1--2
A2--G
G2--10
G3--10
B2--10
B3--10
B3--2
B3--4
B5--10
B6--10
B7--10
B8--10
B8--G
C2--4
C3--4
C4--G
C5--4
D1--2
D1--4
D1--10
D1--10
D1d-G
D1f-G
D1y-G
D5--10
D6--4
D6--10
D7--2
E2--2
E2--4
E2--10
E2b-G
E2--G
E3--2
E3--2
E3--4
E6--2
E7--2
E8--2
E9--2
E10-4
F1--2
01--2
01--4
01--10
01--10
01--10
01--G
08--2

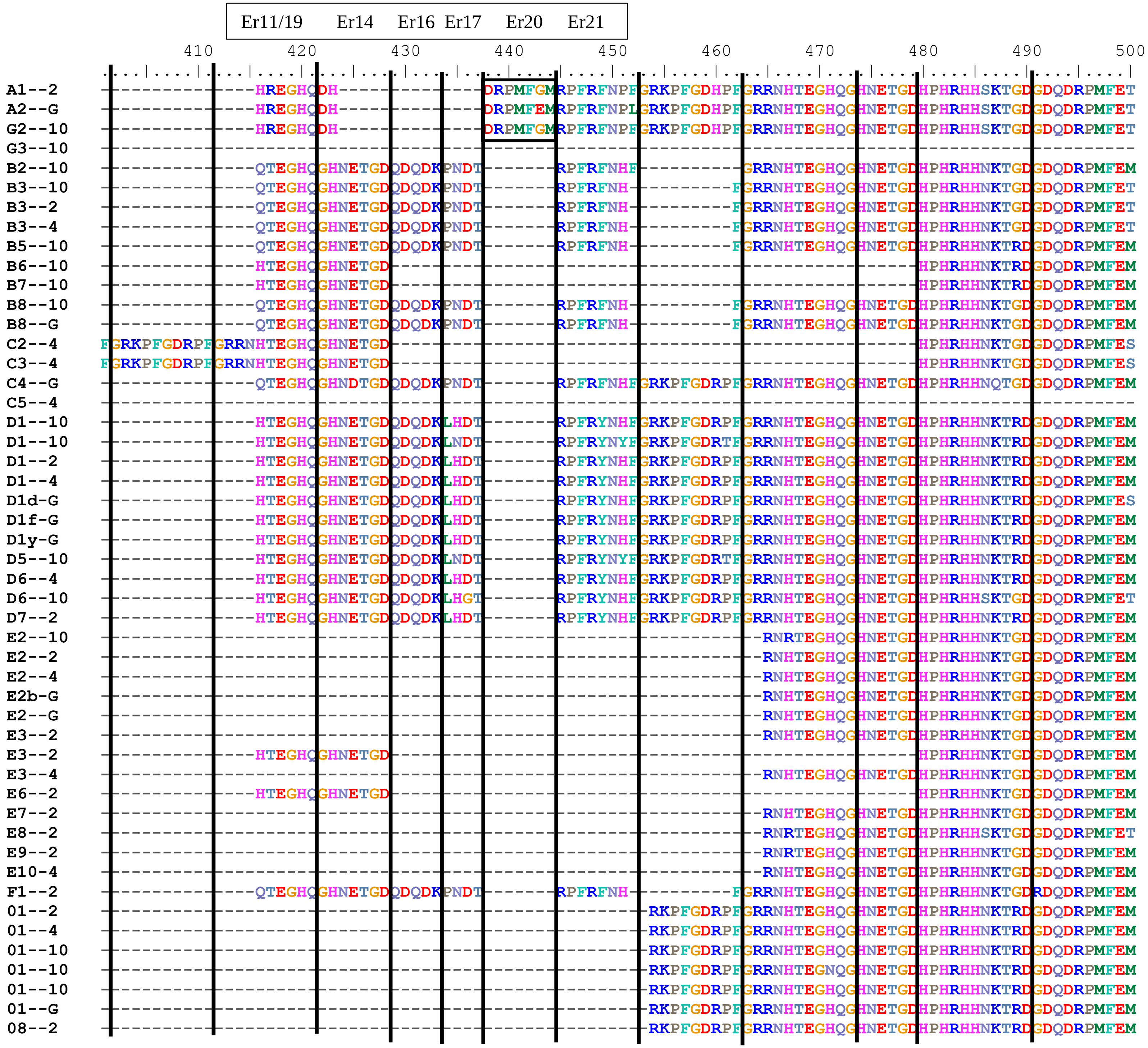

Er11/19 Er14 Er16 Er17 Er20 Er21
410 420 430 440 450 460 470 480 490 500
A1--2
A2--G
G2--10
G3--10
B2--10
B3--10
B3--2
B3--4
B5--10
B6--10
B7--10
B8--10
B8--G
C2--4
C3--4
C4--G
C5--4
D1--10
D1--10
D1--2
D1--4
D1d-G
D1f-G
D1y-G
D5--10
D6--4
D6--10
D7--2
E2--10
E2--2
E2--4
E2b-G
E2--G
E3--2
E3--2
E3--4
E6--2
E7--2
E8--2
E9--2
E10-4
F1--2
01--2
01--4
01--10
01--10
01--10
01--G
08--2

```
                   510       520       530       540       550       560       570       580       590
          ....|....|....|....|....|....|....|....|....|....|....|....|....|....|....|....|....|....|
A1---2    RPFWVNPFGRKPFGDRPFDRRNGTEEGSPRRDGHPHPHGNRGRWGENESEEKEHPTTESVTTSSPLKVIEIAINEVDTNV-----VAEV*
A2---G    RPFWVNPFGRKPFGDRPFDRRNGTEEGSPRRDGHPHPHGNRGRWGENESEEKEHPTTESVTTSSPLKVIEIAINEVDTNV-----VAEV*
G2---10   RPFWVNPFGRKPFGDRPFDRRNGTEEGSPRRDGHPHPHGNRRRWGENESEEKEHPTTESVTTSSPLKVIEIAINEVDTNV-----VAEV*
G3---10   -------FGRKPFGDRPFGRRNGTEEGSPRRDGQRRPYGNRGRWGENESEEKEHPTTESVTTSSPPEVVEIAVNEEDVNV-----VAEV*
B2---10   RPFWVNPFGRKPFGDRPFGRRNGTEEGSPRRDGHRRPYGNRGRWGENESEEKEHPTTESVTTSSPPEVV--AINEEDINV-----VAEV*
B3---10   RPFWVNPFGRKPFGDRPFGRRNGTEEGSPRRDGHRRPYGNRGRWGENESEEKEHPTTESVTTSSPPEVVEIAVNEEDVNV-----VAEV*
B3---2    RPFWVNPFGRKPFGDRPFGRRNGTEEGSPRRDGHRRPHGNRGRWGENESEEKEHPTTESVTTSSPPEVVEIAIND----------VAEV*
B3---4    RPFWVNPFGRKPFGDRPFGRRNGTEEGSPRRDGHRRPYGNRGRWGENESEEKEHPTTESVTTSSPPEVVEIAVNEEDVNV-----VAEV*
B5---10   RPFRFNPFGRKPFGGRPFDRRNGTEEGSPRRDGHRRPYGNRGRWGENESEEKEHPTTESVTTSSPPEVV--AINEEDINV-----VAEV*
B6---10   RPFRFNPFGRKPFGGRPFDRRNGTEEGSPRRDGHRRPYGNRGRWGENESEEKERPTTESVTTSSPPEVVEIAFNEEDVHV-----VAEV*
B7---10   RPFRFNPFGRKPFGGRPFDRRNGTEEGSPRRDGHRRPYGNRGRWGENESEEKERPTTESVTTSSPPEVVEIAFNEEDVNV-----VAEV*
B8---10   RPFWVNPFGRKPFGDRPFGRRNGTEEGSPRRDGHRHPYGNRGRWGENESEEKEHPTTESVTTSSPPEVVEIAVNEEDVNV-----VAEVL
B8---G    RPFWVNPFGRKPFGDRPFGRRNRTEEGSPRRDGHRHPYGNRGRWGENESEEKEHPTTESVTTSSPPEVVEIAVNEEDVNV-----VAEVY
C2---4    RPFRFNPFGRKPFGDRPFGRRNGTEEGSPRRDGHRRPYGNRGRWGENESEEKEHPTTESVTTSSPPEVVEIAVNEEDVNV-----VAEV*
C3---4    RPFRFNPFGRKPFGDRPFGRRNGTEEGSPRRDGHRRPYGNRGRWGENESEEKEHPTTESVTTSSPPEVVEIAFNEEDVNV-----VAEV*
C4---G    RPFRFNPLGRKPFGDRPFGRRNGTEEGSLRRDGHRRPYGNRGRWGENESEEKEHPTTESVTTSSPDVVEIAVNEEDVNV-----VAEV*
C5---4    ---RFNPFGRKPFGDRPFGRRNGTEEGSPRRDGHRRPYGNRGRWGENESEEKEHPTTESVTTSSPPEVVEIAVNEEDVNV-----VAEV*
D1---10   RPFRFNPFGRKPFGGRPFDRRNGTEEGSPRRDGHRRPYGNRGRWGENESEEKEHPTTESVTTSSPPEVV--AINEEDINV-----VAEV*
D1---10   RPFRFNPFGRKPFGGRPFDRRNGTEEGSPRRDGHRRPYGNRGRWGENESEEKEHPTTESVTTSSPPEVV--AINEEDINV-----VAEV*
D1---2    RPFRFNPFGRKPFGGRPFDRRNGTEEGSPRRDGHRRPYGNRGRWGENESEEKEHPTTESVTTSSPPEVV--AINEEDINV-----VAEV*
D1---4    RPFRFNPFGRKPFGGRPFDRRNGTEEGSPRRDGHRRPYGNRGRWGENESEEKEHPTTESVTTSSPPEVV--AINEEDINV-----VAEV*
D1d--G    RPFRFNPFGRKPFGDRLFGRRNGTEEGSSRRDGHRRPYGNRGRWGENESEEKEHPTTESVTTSSPPEVV--AINEEDINV-----VAEV*
D1f--G    RPFRFNPLGRKPFGDRPFGRRNGTEEGSPRRDGQRRPYGNRGR*----------------------------------------------
D1y--G    RPFRFNPFRRKPFGGRPFDRRNGTEEGSPRRDGHRRPYGNRGRWGENESEEKEHPTTESVTTSSPPEVV--AINEEDINV-----VAEV*
D5---10   RPFRFNPFGRKPFGGRPFDRRNGTEEGSPRRDGHRRPYGNRGRWGENESEEKEHPTTESVTTSSPPEVV--AINEEDINV-----VAEV*
D6---4    RPFWVNPFGRKPFGDRPFGRRNGTEEGSPRRDGHRRPYGNRGRWGENESEEKEHPTTESVTTSSPPEVVEIAVNEEDVNV-----VAEV*
D6---10   RPFWVNPFGRKPFGDRPFDRRNGTEEGSPRRDGHPHPHGNRGRWGENESEEKEHPTTESVTTSSPLKVIEIAINEVDTNV-----VAEV*
D7---2    RPFRFNPFGRKPFGGRPFDRRNGTEEGSPRRDGHRRPYGNRGRWGENESEEKEHPTTESVTTSSPPEVV--AINEEDINV-----VAEVK
E2---10   RPFRFNPFGRKPFGDRPFGRRNGTEEGSPRRDGQRRPHGNRGRWGENESEEKEHPTTESVTTYSPP*-----------------------
E2---2    RPFRFNPFGRKPFGDRPFGRRNGTEEGSPRRDGQRRPYGNRGRWGENESEEKEHPTTESVTTSSPP*-----------------------
E2---4    RPFRFNPFGRKPFGDRPFGRRNGTEEGSPRRDGQRRPYGNRGRWGENESEEKEYPTTESVTTSSPP*-----------------------
E2b--G    RPFRFNPFGRKPFGDRPFGRRNGTEEGSPRRDGQRRPYGNRGRWGENESEEKEHPTTESVTTSSPP*-----------------------
E2---G    RPFRFNPFGRKPFGDRPFGRRNGTEEGSPRRDGQRRPYGNRGRWGENESEEKEHPTTESVTTSSPP*-----------------------
E3---2    RPFRFNPFGRKPFGDRPFGRRNGTEEGSPRRDGQRRPYGNRGRWGENESEEKEHPTTESVTTSSPP*-----------------------
E3---2    RPFRFNPFGRKPFGDRPFGRRNGTEEGSPRRDGQRRPYGNRGRWGENESEEKEHPTTESVTTSSPP*-----------------------
E3---4    RPFRFNPFGRKPFGDRPFGRRNGTEEGSPRRDGQRRPYGNRGRWGENESEEKEHPTTESVTTSSPP*-----------------------
E6---2    RPFRFNPFGRKPFGDRPFGRRNGTEEGSPRRDGQRRPYGNRGRWGENESEEKEYPTTESVTTSSPP*-----------------------
E7---2    RPFRFNPFGRKPFGDRPFGRRNGTEEGSPRRDGQRRPYGNRGRWGENESEEKEHPTTESVTTSSPPEVVAI--NEEDINV-----VAEV*
E8---2    RPFWVNPFGRKPFGDRPFDRRNGTEEGSPRRDGHPHPHGNRGRWGENESEEKEHPTTESVTTSSPLKVIEIAINEVDTNV-----VAEV*
E9---2    RPFRFNPFGRKPFGDRPFGRRNGTEEGSPRRDGQRRPHGNRGRWGENESEEKEHPTTESVTTYSPP*-----------------------
E10--4    RPFRFNPFGRKPFGDRPFGRRNGTEEGSPRRDGQRRPYGNRGRWGENESEEKEHPTTESVTTSSPP*-----------------------
F1---2    RPFWVNPFGRKPFGDRPFGRRNGTEEGSPRRDGHRHPYGNRGRWGENESEEKEHPTTESITTSSPPEVVEIAVNEEDVNV-----VAEV*
01---2    RPFRFNPFGRKPFGDRPFGRRNGTEEGSPRRDGQRRPYGNRGRWGENESEEKEHPTMESVTTSSPPEVVEIA-----INEVDTNGVAEV*
01---4    RPFRFNPFGRKPFGDRPFGRRNGTEEGSPRRDGHRRPYGNRGRWGENESEEKEHPTTESVTTSSPPEVVEIA-----IND-----VAEV*
01---10   RPFRFNPFGRKPFGDRPFGRRNGTEEGSPRRDGQRRPHGNRGRWGENESEEKEHPTTESVTTSSPPEVVEIA-----IND-----VAEV*
01---10   RPFRFNPFGRKPFGDRPFGRRNGTEEGSPRRDGQRRPHGNRGRWGENESEEKEHPTTESVTTSSPPEVVEIAIND----------VAEV*
01---10   RPFRFNPFGRKPFGDRPFGRRNGTEEGSPRRDGQRRPHGNRGRWGENESEEKEHPTTESVTTSSPPEVVEIA-----IND-----VAEV*
01---G    RPFRFNPFGRKPFGDRPFGRRNGTEEGSPRRDGQRRPHGNRGRWGENESEEKEHPTTESVTTSSPDVVEIA-----IND-----VAEV*
08---2    RPFRFNPFGRKPFGDRPFGRRNGTEEGSPRRDGQRRPYGNRGRWGENESEEKEYPTTESVTTSSPP*-----------------------
```

Figure S3. The MLGT-PRANK alignment of representative SpTrf protein sequences with a provided maximum likelihood guide tree results in regions of misaligned sequence. The MLGT-PRANK alignment was done using webPRANK with the deduced protein sequences as described in Figure S1. Explanations of protein names, dashes, and asterisks are the same as in Figure S1. Element boarders based on the repeat-based alignment are indicated by vertical black lines. Horizontal black lines indicate regions where non-matching elements overlap. Notes in the alignment and elements of interest are labeled above those regions. Er is used as an abbreviation to indicate elements in the repeat-based alignment. The maximum likelihood tree generated from the manual repeat-based alignment (Figure 1B in the main paper) was provided as a guide tree for this PRANK alignment. Standard parameters were used in webPRANK (gap rate, 0.05; gap length, 5; K, 2.0) and the sequences were set to align by translated codons and to trust insertions (+F). Substitution scoring was set to relaxed.

```
                10        20        30        40        50        60        70        80        90       100
       ....|....|....|....|....|....|....|....|....|....|....|....|....|....|....|....|....|....|....|....|
A1--2  AHAQSDFNERRGKENGRERGQDRFGGRPDGMQMGGPRQDGGPMGGRRFDGPRFGAPQMGGPRQNGGPMGGRRFDGPGFGAPPMGGPRQDGGPMGGRRFDG
A2--G  AHAQSDFNERRGKENGRERGQDRFGGRPDGMQMGGPRQDGGPMGGRRFDGPRFGAPQMGGPRQNGGPMGGRRFDGPGFGAPPMGGPRQDGGPMGGRRFDG
G2--10 AHARRDFNELRGKENGRERGQGRFGGRPDGMQMGGPRQDGGPMGGRRFDGPGFGAPQMGGPRQNGGPMGGRRFDGPRFGAPPMGGPRQDGGPMGGRRFDG
G3--10 AHARRDFNELRGKENGRERGQGRFGGRPDGMQMGGPRQDGGPMGGRRFDGPGFGAPQMGGPRQNGGPMGGRRFDGPRFGAPPMGGPRQDGGPMGGRRFDG
B2--10 AHAQRDYNELRGNKNGRERGQGRFGGRPGGMQMGGSRQDGGPMGGRRFDGPDSGAPQMDGRRQDGGPMGGRRFDGPGFGAPEMDGRRQ------------
B3--10 AHARRDYNERRGNENGRERGQGRFGGRPGGMQMGGPRQDGGPMGGRRFDGP-------------------------GFGAPQMDGRRQ------------
B3--2  AHARRDYNERRGNENGRERGQGRFGGRPGGMQMGGPRQDGGPMGGRRFDGP-------------------------GFGAPQMDGRRQ------------
B3--4  AHARRDFNERRGNENGRERGQDRFGGRPGGMQMGGSRQDGGPMGGRRFDGP-------------------------GFGAPHMDGRRQ------------
B5--10 AHARRDFNERRGNENGRERGQGRFGGRPGGMQMGGSRQDGGPMGGRRFDGP-------------------------GFGAPHMDGRRQ------------
B6--10 AHAQRDFNEQRGEENGRKRGQGRFRGRPGGMLMGGPRQDGGSMGGRRFDGP-------------------------GLGAPQMDGRRQ------------
B7--10 AHAQRDYNELRGNKNGRERGQGRFGGRPGGMQMGGSRQDGGPMGGRRFDGP-------------------------DSGAPQMDGRRQ------------
B8--10 AHARRDFNERRGNENGRERGQGRFGGRPGGMQMGGSRQDGGPMGGRRFDGP-------------------------GFGAPHMDGRRQ------------
B8--G  AHARRDFNERRGNENGRERGQGRFGGRPGGMQMGGSRQDGGPMGGRRFDGP-------------------------GFGAPHMDGRRQ------------
C2--4  AHAQRDYNELRGNKNGRERGQGRFGGRPGGMQMGGSRQDGGPMGGRRFDGP-------------------------DSGAPQMDGRRQDGGPMGGRRFDG
C3--4  AHAQRDYNERRGNENGRERGQGRFGGRPGGMQMDGRRQDGGPMGGRRFDGP-------------------------GFGAPQMDGRRQ------------
C4--G  AHARRDFNERRGRENGRKRGQGGFGGRPDGMQM-------------------------------------------------------------------
C5--4  AHAQRDYNELRGNKNGRERGQGRFGGRPGGMQMGGSRQDGGPMGGRRFDGP-------------------------DSGAPQMDGRRQ------------
D1--10 AHAQRDYNELRGNENGRERGQGRFGGRPGGMQMGGPRQDGGPMGGRRFDGP-------------------------DSGAPQMDGRRQDGGPMGGRGFDG
D1--10 AHAQRDYNELRGNKNGRERGQGRFGGRPGGMQMGGSRQDGGPMGGRRFDGP-------------------------DSGAPQMDGRRQDGGPMGGRRFDG
D1--2  AHAQRDYNELRGNKNGRERGQGRFGGRPGGMQMGGSRQDGGPMGGRRFDGP-------------------------DSGAPQMDGRRQDGGPMGGRRFDG
D1--4  AHAQRDYNELRGNKNGRERGQGRFGGRPGGMQMGGSRQDGGPMGGRRFDGP-------------------------DSGAPQMDGRRQDGGPMGGRRFDG
D1d-G  AHAQRDYNERRGNENGRERGQGRFGGRPGGMQMGGSRQDGGPMGGKRFDGP-------------------------DSGAPQMDGRRQDGGPMGGRRFDG
D1f-G  AHAQRDYNERRGNENGRERGQGRFGGRPGGMQTGGPRQDGGPMGGRRFDGP-------------------------DSGAPQMDGRRQDGGPMGGRRFDG
D1y-G  AHAQRDYNELRGNKNGRERGQGRFGGRPGGMQMGGSRQDGGPMGGRRFDGP-------------------------DSGAPQMDGRRQDGGPMGGRRFDG
D5--10 AHAQRDFNEQRGEENGRKRGQGRFRGRPGGMQMGGPRQDGGSMGGRRFDGP-------------------------GLGAPQMDGRRQNGGPMGGRRFDG
D6--4  AHAQRDYNELRGNKNGRERGQGRFGGRPGGMQMGGSRQDGGPMGGRRFDGP-------------------------DSGAPQMDGRRQDGGPMGGRRFDG
D6--10 AHARRDYNERRGNENGRERGQGRFGGRPGGMQMGGPRQDGGPMGGRRFDGP-------------------------DSGSPQMDGRRQDGGPMGGRRFDG
D7--2  AHAQRDYNELRGNKNGRERGQGRFGGRPGGMQMGGSRQDGGPMGGRRFDGP-------------------------DSGAPQMDGRRQDGGPMGGRRFDG
E2--10 AHAQRDFNERRGKENDTERGQGGFGGRPGGMQMGGPRQDGGPMGGRRFDGP-------------------------ESGAPQMEGRRQ------------
E2--2  AHAQRDFNERRGKENDTERGQGGFGGRPGGMQMGGPRQDGGPMGGRRFDGP-------------------------ESGAPQMEGRRQ------------
E2--4  AHAERDFNERRGKENGRERGQGGFGGRPGGMQTGSPRQDGGPMGGMRFDGP-------------------------ESGAPQMDGRRQ------------
E2b-G  AHARRDFNERRGKENGTERGQGGFGGRPGGMQTGSPRQD----GGRRFDGP-------------------------ESGAPQMDGRRQ------------
E2--G  AHAQRDFNERRGKENDTERGQGGFGGRPGGMQMGSPRQDGGQMGGRRFDGP-------------------------ESGAPQMEGRRQ------------
E3--2  AHAQRDYNELRGNKNGRERGQGRFGGRPGGMQMGGSRQDGGPMGGRRFDGP-------------------------DSGAPQMDGRRQDGGPMGGRRFDG
E3--2  AHARRDFNERRGKENGRERGQGGFGGRPGGMQTGSPRQDDGPMGGRRFDGP-------------------------ESGAPQMDGRRQ------------
E3--4  AHAQRDFNERRGKENDTERGQGGFGGRPGGMQMGGPRQDGGPMGGRRFDGP-------------------------ESGAPQMEGRRQ------------
E6--2  AHAQRDYNELRGNKNGRERGQGRFGGRPGGMQMGGSRQDGGPMGGRRFDGP-------------------------DSGAPQMDGRRQDGGPMGGRRFDG
E7--2  AHAERDFNERRGKENGRERGQGGFGGRPGGMQTGSPRQDGGPMGGMRFDGP-------------------------ESGAPQMDGRRQ------------
E8--2  AHAQRDFNERRGKENDTERGQGGFGGRPGGMQMGGPRQDGGPMGGRRFDGP-------------------------ESGAPQMDGRRQ------------
E9--2  AHAQRDFNERRGKENDTERGQGGFGGRPGGMQMGGPRQDGGPMGGRRFDGP-------------------------ESGAPQMDGRRQ------------
E10-4  AHARRDFNELRGKENGRERGQGRFGGRPDGMQMGGPRQDGGPMGGRRFDGP-------------------------GFGAPQMGGPRQNGGPMGGRRFDG
F1--2  AHARRDFNERRGNENGRERGQGRLGAKPGGMQMGGSRQDGGPVVGRRFDGP-------------------------GFGAPHMDGRRQ------------
01--2  AHARRDFNERRGKENGRERGQGGFGGRPGGMQTGSPRQDGGPMGGMRFDGP-------------------------ESGAPQMDGRRQ------------
01--4  AHARRDFNERRGKENGRERGQGGFGGRPGGMQTGSPRQDGGPMGGMRFDGP-------------------------ESGAPQMDGRRQ------------
01--10 AHARRDFNERRGKENGRERGQGGFGGRPGGMQTGSPRQDGGPMGGRRFDGP-------------------------ESGAPQMDGRRQ------------
01--10 AHARRDFNERRGKENGRERGQGGFGGRPGGMQTGSPRQDGGPMGGRRFDGP-------------------------ESGAPQMDGRRQ------------
01--10 AHAQRDYNERRGNENGRERGQGRFGGRPGGMQMDGRRQDGGPMGGRRFDGP-------------------------GFGAPQMDGRRQ------------
01--G  AHARRDFNERRGKENGRERGQGGFGGRPGGMQTGSPRQDGGPMGGMRFDGP-------------------------ESGAPQMDGRRQ------------
08--2  AHARRDFNERRGKENGRERGQGGFGGRPGGMQTGSPRQDGGPMGGMRFDGP-------------------------ESGAPQMDGRRQ------------
```

L-Er7 & S-Er8

```
                 110       120       130       140       150       160       170       180       190       200
             ....|....|....|....|....|....|....|....|....|....|....|....|....|....|....|....|....|....|....|....|
A1--2        PGFGAPQMGGPRQNGGPMGGRRFDGPGFGGSRPDGAGGRPFFGEGGRRGDGEEETDAAQQIGDGLGGPGRCDGPGHGHYGHHQGAGRPFFGNPPPFNPEQ
A2--G        PGFGAPQMGGPRQNGGPMGGRRFDGPRFGGSRPDGAGGRPFFGEGGRRGDGEEETDAARQIG-----PGRFDGPGHGHYGHHQGAGRPFFGNPPPFNPEQ
G2--10       PGFGAPQMGGPRQNGGPMGGRRFDGPGFGGSRPDGAGGRPFFGEGSRRGDGEEETDAARQIGDGLGGPGRFDGPGHGHYGHHQGAGRPFFGNPPPFNPEQ
G3--10       PGFGAPQMGGPRQNGGPMGGRRFDGPGFGGSRPDGAGGRPFFGEGSRRGDGEEETDAARQIGDGLGGPGRFDGPGHGHYGHHQGAGRPFFGNPPPFNPEQ
B2--10       -------------NGGPMGGRRFDGPGFGGSRPDGAGGRPFFGQGGRRGDGEEETDAAQQIGDGLGGPGQFDGHGRRHHGHRQGP-----PQDRPEEQPF
B3--4        -------------NGGPMGGRRFDGPGFGGSRPDGAGGRPFFGQGGRRGDGEEETDAAQQIGDGLGGRGRFDGHGRRHHGHRQGP-----PQDRPEEQPF
B3--10       -------------NGGPMGGRRFDGPRFGGSRPDGAGGRPFFGQGGRRGDGEEETDAAQQIGDGLGGRGQFDGHGRRHHGHRQGP-----PQDRPEEQPF
B3--2        -------------NGGPMGGRRFDGPRFGGSRPDGAGGRPFFGQGGRRGDGEEETDAAQQIGDGLGGRGQFDGHGRRHHGHRQGP-----PQDRPEEQPF
B5--10       -------------NGGPMGGRRFDGPGFGGSRPDGAGGRPFFGQGGRRGDGEEETDAAQQIGDGLGGRGQFDGHGRRHHGHRQGP-----PQDRPEEQPF
B6--10       -------------NGGPMGGRRFDGPGFGGSRPDGAGGRPFFGQGGRRGDGEEETDAAQQIGDGLGGPGQFDGHGRRRHGHRQGP-----PQDRSEEQPF
B7--10       -------------NGGPMGGRRFDGPGFGGSRPDGAGGRPFFGQGGRRGDGEEETDAAQQIGDGLGGSDRFDGLRRGHHGHRQGP-----PQDRPEEQPF
B8--10       -------------NGGPMGGRRFDGPGFGGSRPDGAGGRPFFGQGGRRGDGEEETDAAQQIGDGLGGRGQFDGHGRRHHGHRQGP-----PQDRPEEQPF
B8--G        -------------NGGPMGGRRFDGPGFGGSRPDGAGGRPFFGQGGRRGDGEEETDASQQIGDGLGGRGQFDGHGRRHHGHRQGP-----PQDRPEEQPF
C2--4        PGFGAPEMDGRRQNGGPMGGRRFDGPGFGGSRPDGAGGRPFFGQGGRRGDGEEETDAAQQIGDGLGGSDRFDGPRRGHHGHRQGP-----PQDRPEEQPF
C3--4        -------------NGGPMGGRRFDGPGFGGSRPDGAGGRPFFGQGGRRGDGEEETDAAQQIGDGLGGSDRFDGPRRGHHGHRQGP-----PQDRPEEQPF
C4--G        ------------------GGRRFDGPGFGGSRPDGAG-RPFFGQGGKRGDGEEETDAAQQIGDGLGGSDRFDGPRRGHHGHRQGP-----PQDRPEEQPF
C5--4        -------------NGGPMGGRRFDGPGFGGSRPDGAGGRPFFGQGGRRGDGEEETDAAQQIGDGLGGSDRFDGPRRGHHGHRQGP-----PQDRPEEQPF
D1--10       PGFGAPEMDGRRQNGGPMGGRRFDGPGFGGSRPVGAGGRLFFGQGGRRGDEEEETDAAQQIGDGLGGPGQFDGPGRRHHGHRQGH-----PQDQAEEQPF
D1--10       PGIGAPEMDGTEKNGGPMGGRELDGPGFGGSRPDGGGGRPFFGQGGRRGDGEEETDAAQQIGDGLGGPGQFDGPGRRHHGHRRGH-----PQDQAEEQPF
D1--2        PGFGAPEMDGRRQNGGPMGGRRFDGPGFGGSRPDGAGGRPFFGQGGRRGDGEEETDAAQQIGDGLGGPGQFDGPGRRHHGHRQGH-----PQDQAEEQPF
D1--4        PGFGAPEMDGRRQNGGPMGGRRFDGPGFGGSRPDGAGGRPFFGQGGRRGDGEEETDAAQQIGDGLGGPGQFDGPGRRHHGHRQGH-----PQDQAEEQPF
D1d-G        PGFGAPEMDGRRQNGGPMGGRRFDGPGFGGSRPVGAGGRPFFGQGGRRGDGEEETDAAQQIGDGLGGPGQFDGPGRRHHGHRQGH-----PQDQAEEQPF
D1f-G        PGFGAPEMDGRRQNGGPMGGRRFDGPGFGGSRPVGAGGRPVFGQGGRRGDGEEETDAAQQIGDGLGGPGQFDGPGRRHHGHRQGH-----PQDQAEEQPF
D1y-G        PGFGAPEMDGRRQNGGPMGGRRFDGPGFGGSRPDGAGGRPFFGQGGRRGDGEEETDAAQQIGDGLGGPGQFDGPGRRHHGHRQGH-----PQDQAEEQPF
D5--10       P-------------------------GFGGSRPDGAGGRPFFGQGGRRGDGEEETDAAQQIGDGLGGPGQFDGPGRRHHGHRRGH-----PQDQAEEQPF
D6--4        PGFGAPEMDGRRQNGGPMGGRRFDGPGFGGSRPDGAGGRPFFGQGGRRGDGEEETDAAQQIGDGLGGPGQFDGPGRRHHGHRQGH-----PQDQAEEQPF
D6--10       PGFGAPEMDGRRQNGGPMGGRRFDGPGFGGSRPDGAGGRPFFGQGGRRGDGEEETDAAQQIGDGLGGPGQFDGPGRRHHGHRQGH-----PQDQAEEQPF
D7--2        PGFGAPEMDGRRQNGGPMGGRRFDGPGFGGSRPDGAGGRPFFGQGGRRGDGEEETDAAQQIGDGLGGPGQFDGPGRRHHGHRQGH-----PQDQAEEQPF
E10--4       PGFGAPQMGGPRQNGGPMGGRRFDGPGFGGSRPDCAGGRPFFGEGGRRGDGEEETDAARQIGDGLGGPGQFDGPRRGHHGHRQGP-----PQDRPEEQPF
E2--10       -------------NGGPMGGRRFDGPRFGGSRPDGAGGRPFFGQGGRRGDGEEETDDDQKMGDGPGGRGQFDGHGRGHHGHRQGP-----PQDRPEEQPF
E2--2        -------------NGGPMGGRRFDGPRFGGSRPDGAGGRPFFGQGGRRGDGEEETDAAQQIGDGLGGRDQFDGHGRGHHGHRQGP-----PQDRPEEQPF
E2--4        -------------NGGPMGGRRFDGPRFGGSRPDGAGGRPFFGQGGRRGDGKEETDAAPLIGDGLGGRGQFDGHGRGHHGHRQGP-----PQDRPEEQPF
E2b-G        -------------NGGPMGGRRFDGPRFGGSRPDGAGGRPFFGQGGRRGDGEEETDAAQQIGDGLGGSGQFDGPRRGHHGHRQGP-----PQDRPEEQPF
E3--2        -------------NGGPMGGRRFDGPRFGGSRPDGAGGRPFFGQGGRRGDGEEETDAAQQIGDGLGGSGQFDGPRRGHHGHRQGP-----PQDRPEEQPF
E3--4        -------------NGGPMGGRRFDGPGFGGSRPDGAGGRPFFGPGGRRGDGEEETDAALLIGDVLGGRGQFDGPRRGHHGHRQGP-----PQDRPEEQPF
E3--2        P-------------------------GFGGSRPDGAGGRPFFGQGGRRGDGEEETDAAQQMGDGLGGRGQFDGPRRGHHGHRQGP-----PQDRPEEQPF
E6--2        PGFGAPEMDGRRQNGGPMGGRRFDGPGFGGSRPVGAGGRPFFGQGGRRGDGEEETDAAQQIGDGLGGRGQFDGHGRGHHGHRQGP-----PQDRPEEQPF
E7--2        -------------NGGPMGGRRFDGPRFGGSRPDGAGGRPFFGQGGRRGDGKEETDAAQQIGDGLGGRGQFDGHGRGHHGHRQGP-----PQDRPEEQPF
E8--2        -------------NGGPMGGRRFDGPRFGGSRPDGAGGRPFFGQGGRRGDGEEETDAAQQIGDGPGGRGQFDGHGRGHHGHRQGP-----PQDRPEEQPF
E9--2        -------------NGGPMGGRRFDGPRFGGSRPDGAGGRPFFGQGGRRGDGEEETDAAQQIGDGPGGRGQFDGHGRGHHGHRQGP-----PQDRPEEQPF
F1--2        -------------NGGPMGGRRFDGPGFGGSRPDGAGGRPFFGQGGRRGDGEEETDAAQQIGDGLGGRGQFDGHGRRHHGHRQGP-----PQDRPEEQPF
E2--G        -------------NGGPMGGRRFDGPRFGGSRPDGAGGRPFFGQGGRRGDGEEETDAAQQIGDGLGGRGQFDGHGRGHHGHRQGP-----PQDRPEEQPF
01--2        -------------NGGPMGGRRFDGPRFGGSRPDGAGGRPFFGQGGRRGDGEEETDAAQQIGDGLGGPGQFDGPGRRHH---------------------
01--4        -------------NGGPMGGRRFDGPGFGGSRPDGAGGRPFFGQGGRRGDGEEETDAAQQIGDGLGGPGQFDGPGRRHH---------------------
01--10       -------------NGGPMGGRRFDGPRFGGSRPDGAGGRPFFGQGGRRDDGEEETDAAQQIGDGLGGPGQFDGPGRRHH---------------------
01--10       -------------NGGPMGGRRFDGPGFGGSRPDGAGGRPFFGQGGRRGDGEEETDAAQQIGDGLGGPGQFDGPGRRHH---------------------
01--10       -------------NGGPMGGRRFDGPGFGGSRPDGAGGRPFFGQGGRRGDGEEETDAAQQIGDGLGGPGQFDGPGRRHH---------------------
01--G        -------------NGGPMGGRRFDGPGFGGSRPDGAGGRPFFGQGGRRGDGEEETDAAQQIGDGLGGPGQFDGPGRRHH---------------------
08--2        -------------NGGPMGGRRFDGPRFGGSRPDGAGGRPFFGQGGRRGDGEEETDAAQQIGDGLGGPGQFDGPGRRHH---------------------
```

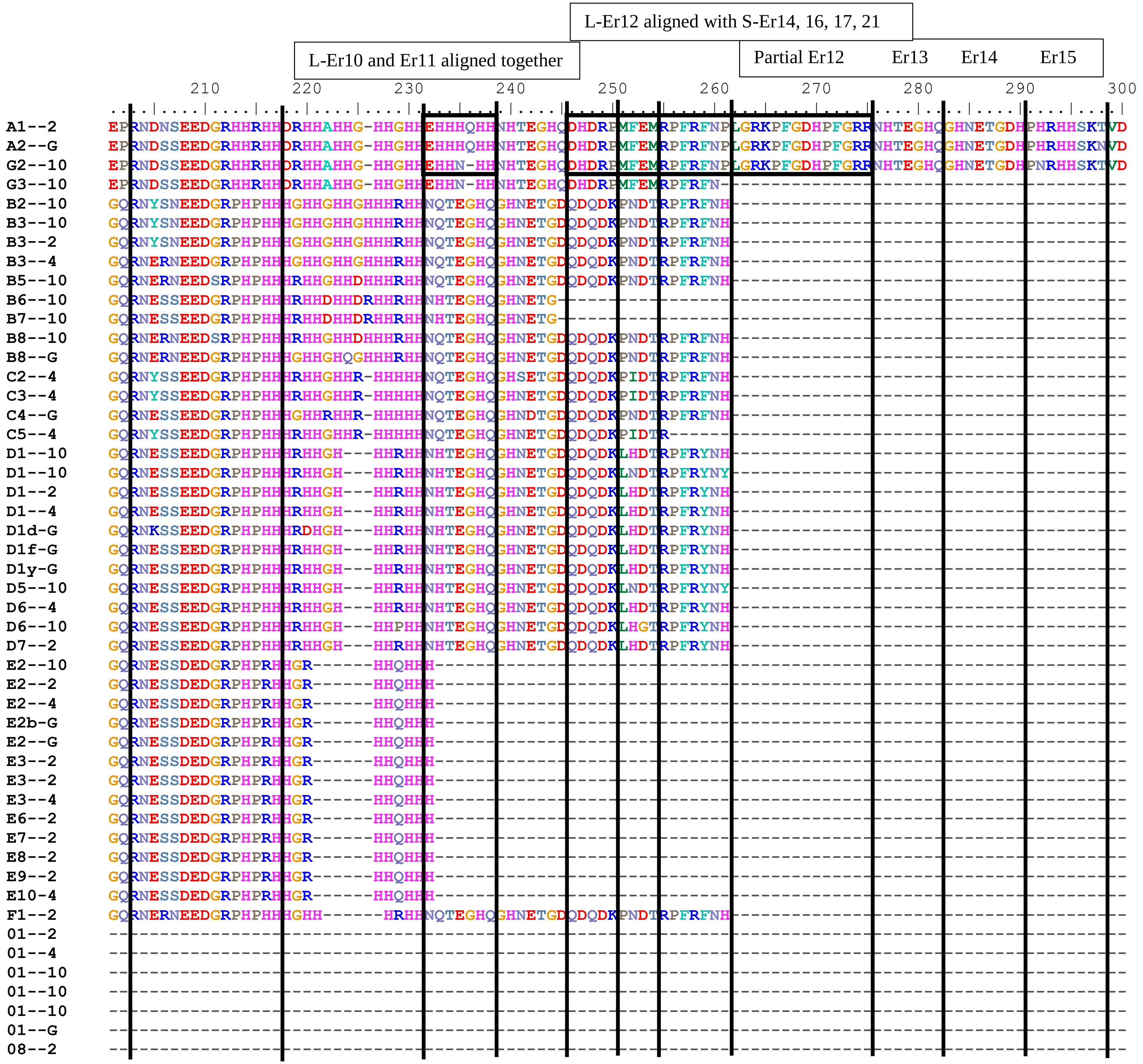
L-Er12 aligned with S-Er14, 16, 17, 21
L-Er10 and Er11 aligned together
Partial Er12
Er13
Er14
Er15
210 220 230 240 250 260 270 280 290 300

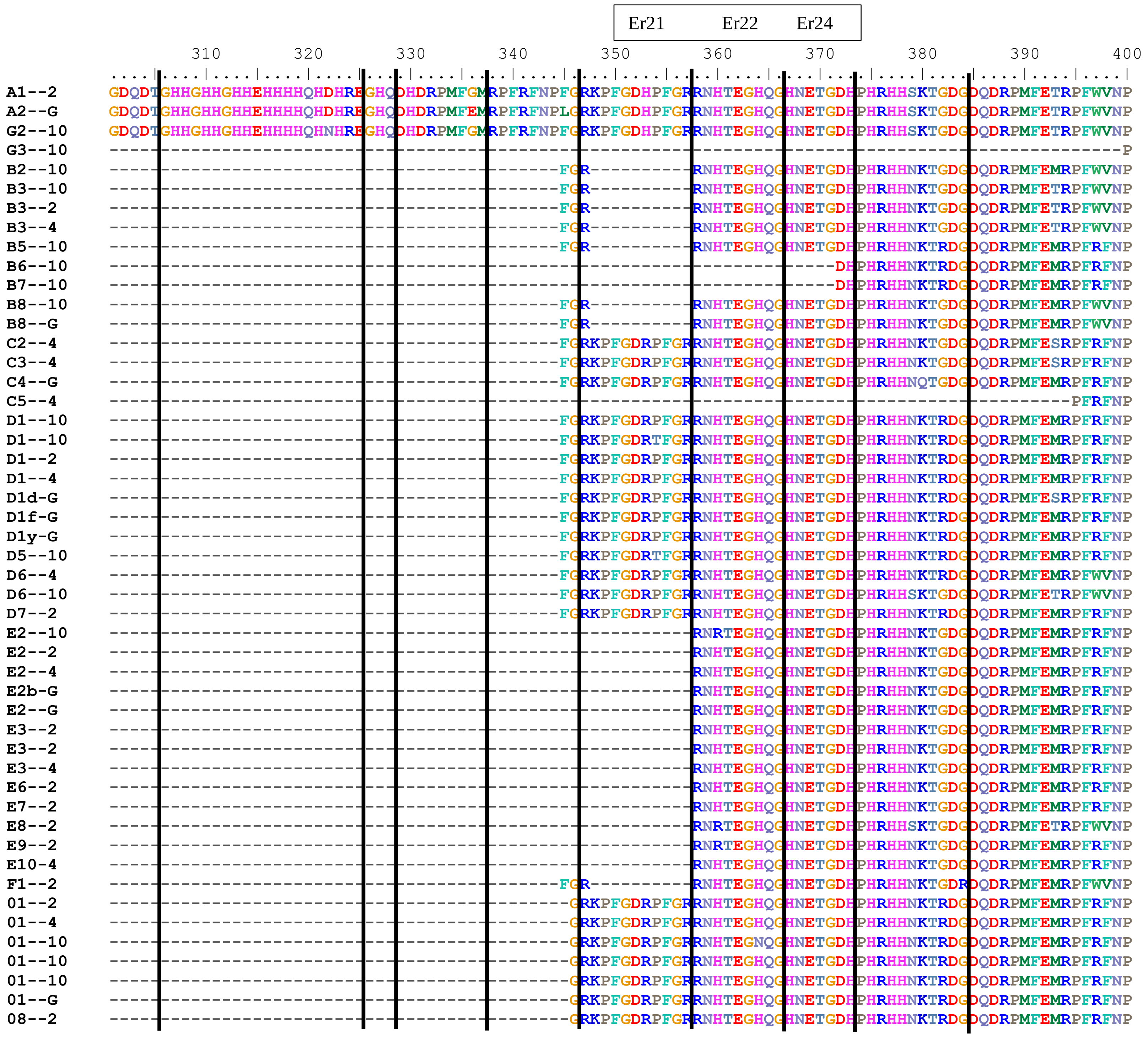

Er21
Er22
Er24
310
320
330
340
350
360
370
380
390
400

```
                         410       420       430       440       450       460       470
              ....|....|....|....|....|....|....|....|....|....|....|....|....|....|....|...
A1--2         FGRKPFGDRPFDRRNGTEEGSPRRDGHPHPHGNRGRWGENESEEKEHPTTESVTTSSPLKVIEIAINEVDTNVVAEV-
A2--G         FGRKPFGDRPFDRRNGTEEGSPRRDGHPHPHGNRGRWGENESEEKEHPTTESVTTSSPLKVIEIAINEVDTNVVAEV-
G2--10        FGRKPFGDRPFDRRNGTEEGSPRRDGHPHPHGNRRRWGENESEEKEHPTTESVTTSSPLKVIEIAINEVDTNVVAEV-
G3--10        FGRKPFGDRPFGRRNGTEEGSPRRDGQRRPYGNRGRWGENESEEKEHPTTESVTTSSPPEVVEIAVNEEDVNVVAEV-
B2--10        FGRKPFGDRPFGRRNGTEEGSPRRDGHRRPYGNRGRWGENESEEKEHPTTESVTTSSPPEVV--AINEEDINVVAEV-
B3--10        FGRKPFGDRPFGRRNGTEEGSPRRDGHRRPYGNRGRWGENESEEKEHPTTESVTTSSPPEVVEIAVNEEDVNVVAEV-
B3--2         FGRKPFGDRPFGRRNGTEEGSPRRDGHRRPHGNRGRWGENESEEKEHPTTESVTTSSPPEVVEIAIND-----VAEV-
B3--4         FGRKPFGDRPFGRRNGTEEGSPRRDGHRRPYGNRGRWGENESEEKEHPTTESVTTSSPPEVVEIAVNEEDVNVVAEV-
B5--10        FGRKPFGGRPFDRRNGTEEGSPRRDGHRRPYGNRGRWGENESEEKEHPTTESVTTSSPPEVV--AINEEDINVVAEV-
B6--10        FGRKPFGGRPFDRRNGTEEGSPRRDGHRRPYGNRGRWGENESEEKERPTTESVTTSSPPEVVEIAFNEEDVHVVAEV-
B7--10        FGRKPFGGRPFDRRNGTEEGSPRRDGHRRPYGNRGRWGENESEEKERPTTESVTTSSPPEVVEIAFNEEDVNVVAEV-
B8--10        FGRKPFGDRPFGRRNGTEEGSPRRDGHRHPYGNRGRWGENESEEKEHPTTESVTTSSPPEVVEIAVNEEDVNVVAEVL
B8--G         FGRKPFGDRPFGRRNRTEEGSPRRDGHRHPYGNRGRWGENESEEKEHPTTESVTTSSPPEVVEIAVNEEDVNVVAEVY
C2--4         FGRKPFGDRPFGRRNGTEEGSPRRDGHRRPYGNRGRWGENESEEKEHPTTESVTTSSPPEVVEIAVNEEDVNVVAEV-
C3--4         FGRKPFGDRPFGRRNGTEEGSPRRDGHRRPYGNRGRWGENESEEKEHPTTESVTTSSPPEVVEIAFNEEDVNVVAEV-
C4--G         LGRKPFGDRPFGRRNGTEEGSLRRDGHRRPYGNRGRWGENESEEKEHPTTESVTTSSPPDVVEIAVNEEDVNVVAEV-
C5--4         FGRKPFGDRPFGRRNGTEEGSPRRDGHRRPYGNRGRWGENESEEKEHPTTESVTTSSPPEVVEIAVNEEDVNVVAEV-
D1--10        FGRKPFGGRPFDRRNGTEEGSPRRDGHRRPYGNRGRWGENESEEKEHPTTESVTTSSPPEVV--AINEEDINVVAEV-
D1--10        FGRKPFGGRPFDRRNGTEEGSPRRDGHRRPYGNRGRWGENESEEKEHPTTESVTTSSPPEVV--AINEEDINVVAEV-
D1--2         FGRKPFGGRPFDRRNGTEEGSPRRDGHRRPYGNRGRWGENESEEKEHPTTESVTTSSPPEVV--AINEEDINVVAEV-
D1--4         FGRKPFGGRPFDRRNGTEEGSPRRDGHRRPYGNRGRWGENESEEKEHPTTESVTTSSPPEVV--AINEEDINVVAEV-
D1d-G         FGRKPFGDRLFGRRNGTEEGSSRRDGHRRPYGNRGRWGENESEEKEHPTTESVTTSSPPEVV--AINEEDINVVAEV-
D1f-G         LGRKPFGDRPFGRRNGTEEGSPRRDGQRRPYGNRGR*-----------------------------------------
D1y-G         FRRKPFGGRPFDRRNGTEEGSPRRDGHRRPYGNRGRWGENESEEKEHPTTESVTTSSPPEVV--AINEEDINVVAEV*
D5--10        FGRKPFGGRPFDRRNGTEEGSPRRDGHRRPYGNRGRWGENESEEKEHPTTESVTTSSPPEVV--AINEEDINVVAEV-
D6--4         FGRKPFGDRPFGRRNGTEEGSPRRDGHRRPYGNRGRWGENESEEKEHPTTESVTTSSPPEVVEIAVNEEDVNVVAEV-
D6--10        FGRKPFGDRPFDRRNGTEEGSPRRDGHPHPHGNRGRWGENESEEKEHPTTESVTTSSPLKVIEIAINEVDTNVVAEV-
D7--2         FGRKPFGGRPFDRRNGTEEGSPRRDGHRRPYGNRGRWGENESEEKEHPTTESVTTSSPPEVV--AINEEDINVVAEV-
E10-4         FGRKPFGDRPFGRRNGTEEGSPRRDGQRRPYGNRGRWGENESEEKEHPTTESVTTSSPP*------------------
E2--10        FGRKPFGDRPFGRRNGTEEGSPRRDGQRRPHGNRGRWGENESEEKEHPTTESVTTYSPP*------------------
E2--2         FGRKPFGDRPFGRRNGTEEGSPRRDGQRRPYGNRGRWGENESEEKEHPTTESVTTSSPP*------------------
E2--4         FGRKPFGDRPFGRRNGTEEGSPRRDGQRRPYGNRGRWGENESEEKEYPTTESVTTSSPP*------------------
E2b-G         FGRKPFGDRPFGRRNGTEEGSPRRDGQRRPYGNRGRWGENESEEKEHPTTESVTTSSPP*------------------
E2--G         FGRKPFGDRPFGRRNGTEEGSPRRDGQRRPYGNRGRWGENESEEKEHPTTESVTTSSPP*------------------
E3--2         FGRKPFGDRPFGRRNGTEEGSPRRDGQRRPYGNRGRWGENESEEKEHPTTESVTTSSPP*------------------
E3--2         FGRKPFGDRPFGRRNGTEEGSPRRDGQRRPYGNRGRWGENESEEKEHPTTESVTTSSPP*------------------
E3--4         FGRKPFGDRPFGRRNGTEEGSPRRDGQRRPYGNRGRWGENESEEKEHPTTESVTTSSPP*------------------
E6--2         FGRKPFGDRPFGRRNGTEEGSPRRDGQRRPYGNRGRWGENESEEKEYPTTESVTTSSPP*------------------
E7--2         FGRKPFGDRPFGRRNGTEEGSPRRDGQRRPYGNRGRWGENESEEKEHPTTESVTTSSPPEVVAINEEDINVVAEV---
E8--2         FGRKPFGDRPFDRRNGTEEGSPRRDGHPHPHGNRGRWGENESEEKEHPTTESVTTSSPLKVIEIAINEVDTNVVAEV-
E9--2         FGRKPFGDRPFGRRNGTEEGSPRRDGQRRPHGNRGRWGENESEEKEHPTTESVTTYSPP*------------------
F1--2         FGRKPFGDRPFGRRNGTEEGSPRRDGHRHPYGNRGRWGENESEEKEHPTTESITTSSPPEVVEIAVNEEDVNVVAEV-
01--10        FGRKPFGDRPFGRRNGTEEGSPRRDGQRRPHGNRGRWGENESEEKEHPTTESVTTSSPPEVVEIAINDV-----AEV-
01--10        FGRKPFGDRPFGRRNGTEEGSPRRDGQRRPHGNRGRWGENESEEKEHPTTESVTTSSPPEVVEIAINDV-----AEV-
01--10        FGRKPFGDRPFGRRNGTEEGSPRRDGQRRPHGNRGRWGENESEEKEHPTTESVTTSSPPEVVEIAINDV-----AEV-
01--2         FGRKPFGDRPFGRRNGTEEGSPRRDGQRRPYGNRGRWGENESEEKEHPTMESVTTSSPPEVVEIAINEVDTNGVAEV-
01--4         FGRKPFGDRPFGRRNGTEEGSPRRDGHRRPYGNRGRWGENESEEKEHPTTESVTTSSPPEVVEIAINDV-----AEV-
01--G         FGRKPFGDRPFGRRNGTEEGSPRRDGQRRPHGNRGRWGENESEEKEHPTTESVTTSSPPDVVEIAINDV-----AEV-
08--2         FGRKPFGDRPFGRRNGTEEGSPRRDGQRRPYGNRGRWGENESEEKEYPTTESVTTSSPP*------------------
```

Figure S4. The ClustalW alignment without manual correction includes a non-matching region of the deduced SpTrf proteins. The alignment was done using ClustalW in BioEdit with the deduced protein sequences from animals as in Figure S1. Standard parameters were used with full multiple alignment mode. Substitution scoring was set to relaxed. Explanations of protein names, dashes, and asterisks are the same as for Figure S1. Element boarders based on the repeat-based alignment are indicated by vertical black lines. Horizontal black lines indicate regions where non-matching elements overlap. Notes on the alignment and elements of interest are labeled above those regions. Er indicates elements in the repeat-based alignment.

References

1. Cameron RA, Mahairas G, Rast JP, Martinez P, Biondi TR, Swartzell S, et al. A sea urchin genome project: Sequence scan, virtual map, and additional resources. *Proc Natl Acad Sci USA*. (2000) 97(17):9514–8. doi:10.1073/pnas.160261897.
2. Oren M, Barela Hudgell MA, D'Allura B, Agronin J, Gross A, Podini D, et al. Short tandem repeats, segmental duplications, gene deletion, and genomic instability in a rapidly diversified immune gene family. *BMC Genomics*. (2016) 17(1):900. doi:10.1186/s12864-016-3241-x.
3. Barela Hudgell MA, Smith LC. Sequence diversity, locus structure, and evolutionary history of the *SpTransformer* genes in the sea urchin genome. *Front Immunol*. (2021) 12:744783. doi:10.3389/fimmu.2021.744783.
